# Proteoglycans contribute to the functional integrity of the glomerular endothelial cell surface layer and are regulated in diabetic kidney disease

**DOI:** 10.1038/s41598-021-87753-3

**Published:** 2021-04-19

**Authors:** Alina Khramova, Roberto Boi, Vincent Fridén, Anna Björnson Granqvist, Ulf Nilsson, Olav Tenstad, Eystein Oveland, Börje Haraldsson, Kerstin Ebefors, Jenny Nyström

**Affiliations:** 1 Institute of Neuroscience and Physiology, Department of Physiology, Sahlgrenska Academy, University of Gothenburg, Box 432, 40530 Gothenburg, Sweden; 2 Bioscience Renal, Research and Early Development, Cardiovascular, Renal and Metabolism, BioPharmaceuticals R&D, AstraZeneca, Gothenburg, Sweden; 3 Department of Biomedicine, University of Bergen, Bergen, Norway

**Keywords:** Physiology, Glomerulus

## Abstract

All capillary endothelia, including those of the glomeruli, have a luminal cell surface layer (ESL) consisting of glycoproteins, glycolipids, proteoglycans (PGs) and glycosaminoglycans. Previous results have demonstrated that an intact ESL is necessary for a normal filtration barrier and damage to the ESL coupled to proteinuria is seen for example in diabetic kidney disease (DKD). We used the principles of ion exchange chromatography in vivo to elute the highly negatively charged components of the ESL with a 1 M NaCl solution in rats. Ultrastructural morphology and renal function were analyzed and 17 PGs and hyaluronan were identified in the ESL. The high salt solution reduced the glomerular ESL thickness, led to albuminuria and reduced GFR. To assess the relevance of ESL in renal disease the expression of PGs in glomeruli from DKD patients in a next generation sequencing cohort was investigated. We found that seven of the homologues of the PGs identified in the ESL from rats were differently regulated in patients with DKD compared to healthy subjects. The results show that proteoglycans and glycosaminoglycans are essential components of the ESL, maintaining the permselective properties of the glomerular barrier and thus preventing proteinuria.

## Introduction

Albuminuria is the result of a dysfunctional glomerular filtration barrier, which in turn is a symptom common to most glomerulopathies. Damage to any part of the barrier may cause albuminuria and several studies have specifically provided experimental evidence for the requirement of an intact glomerular endothelial surface layer (ESL) to prevent albumin leakage^1–5^. The appearance of the glomerular ESL is that of a highly negatively charged gel with two components: the glycocalyx, which refers to membrane-bound proteoglycans (PG), and the endothelial cell coat that contains secreted PGs, negatively charged glycosaminoglycans (GAG), glycoproteins and soluble proteins (either plasma- or endothelium-derived). PGs are a family of proteins that have one or more GAG chains covalently attached to their core protein. The GAG chains are strongly negatively charged due to their sulphatation. PGs are not only important for being negatively charged building blocks for the ESL but also for their interaction with different extracellular ligands, influencing for example growth factor activity and cytokine release^6^. The glomerular ESL should therefore not be regarded as a static structure, but rather as a dynamic compartment with significant molecular turnover and flexibility^7^. Earlier work by us and other groups have identified some of the PGs produced by the glomerular endothelial cells and demonstrated their importance for glomerular function^1,8^. We have specifically shown that the content of GAGs and their charged moieties are important for normal glomerular function^9^.

Increasing evidence suggests that glomerular endothelial cell dysfunction is an early event in the development of diabetic kidney disease (DKD). An altered glycocalyx has been suggested to lead to proteinuria and loss of renal function^10–12^. Patients with diabetes mellitus have a reduced endothelial glycocalyx layer^13^ and loss of glomerular charge selectivity has been observed in diabetic patients with albuminuria^14^. Although the importance of the ESL has been highlighted in recent years, especially in DKD, the knowledge of its composition and function is still partially unknown and hence we aim to determine the role of the ESL in these conditions. Firstly, we eluted the ESL from rat glomeruli in vivo to examine its composition. Earlier studies by us demonstrated that perfusion with solutions containing different amounts of salt reduced the ESL and modulated the fractional clearance of albumin reversibly^15^. A short bolus of 1 M NaCl perfusate revealed that charge interactions between proteins in the ESL could be broken^2^. In the present study we used the same high salt concentration but with an extended perfusion time to facilitate release of large PGs. The eluate was analyzed using mass spectrometry and PGs were identified. Secondly, to confirm translatability and relevance in renal disease we explored the gene expression of PGs and PG related genes in glomeruli from renal biopsies comparing the profile in patients with DKD with healthy donors. Next, we confirmed the localization of the discovered PGs to the human glomerular endothelium. Finally, we studied the expression of the PGs in vitro in a diabetic milieu.

## Results

### Renal morphology

**Figure 1:**
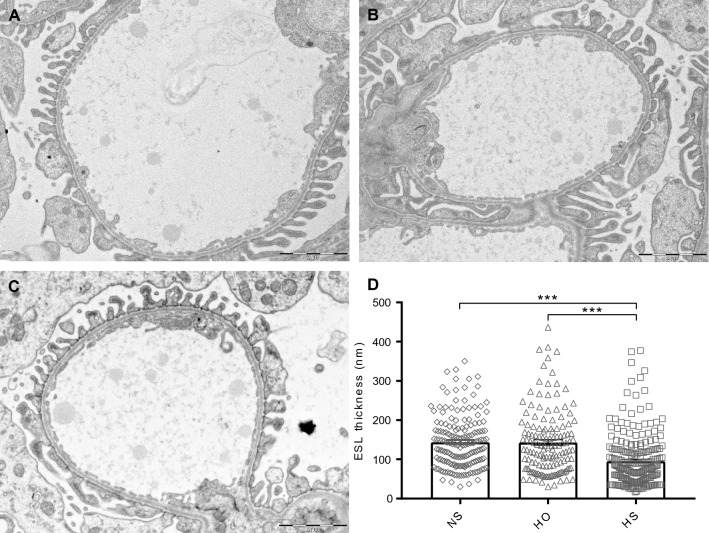
Glomerular morphology and endothelial surface layer thickness 10 min after perfusion. Representative pictures of glomerular capillaries from a rat perfused with physiological salt (NS) (**A**), high osmolarity (HO) (**B**) and high salt (HS) solution (**C**). The thickness of the ESL was estimated by measuring the distance between infused intralipid droplets and the endothelial cells. ESL thickness was reduced in the rats perfused with HS (**D**). Scale bar represents 2 µm. ***P < 0.001, error bars represent SEM.

We eluted the ESL from the renal endothelium using a high salt solution (HS) which breaks the anionic non-covalent bonds and elutes highly negatively charged molecules. High osmolality solution (HO) was used to distinguish between the charge component and osmotic forces, and perfusion with physiological salt solution (NS) was used as control. As our focus was to understand the role of the ESL specifically in the glomerulus we used transmission electron microscopy (TEM) to study the glomerular morphology and ESL thickness (Fig. 1A–C). Glomerular ESL thickness was determined by a method where Intralipid droplets were used as indirect markers to estimate the ESL thickness from electron micrographs^2,5,16^. ESL was significantly reduced (p < 0.001, Fig. 1D) in HS perfused rats compared to rat kidneys perfused with NS or HO solution, proving that the HS is the most efficient in eluting the components of the ESL. The same micrographs demonstrated that the glomerular basement membrane thickness was slightly, but significantly, increased in HS perfused rat kidneys compared with NS (P < 0.01) and decreased in HO compared to NS (P < 0.05) (Table 1). There was no difference in the width of podocyte foot processes or slit diaphragms between the groups (Table 1).

**Table 1:** Renal morphology measurements in rats after elution with normal salt (NS), high osmolarity (HO) or high salt (HS).

	NS	HO	HS
Glomerular basement membrane thickness (nm)	187.71 ± 1.66	183.09 ± 1.97*	195.94 ± 1.54**
Podocyte foot process width (nm)	342.54 ± 9.05	355.76 ± 10.61	351.88 ± 9.70
Podocyte filtration slit width (nm)	45.82 ± 0.96	44.78 ± 1.22	44.86 ± 1.23

Measurements from glomerular capillaries from 5 rats/elution (capillaries; NS n = 103, HS n = 143, and HO n = 133). * P<0.05, **P<0.01 compared to NS

### Analysis of GFR in response to loss of ESL

**Figure 2:**
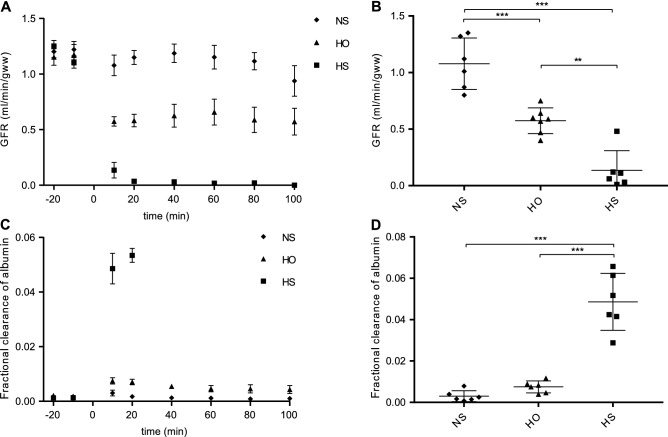
Glomerular filtration rate (GFR) and fractional clearance of albumin. Differences in GFR over time for three different groups of rats that were perfused for 3 min with physiological salt solution (NS), high osmolarity control (HO) and high salt (HS), respectively (**A**). 10 min after the perfusion the GFR had decreased to around half of the GFR at the start of the experiment in the rats perfused with HO, and even further in the rats perfused with HS (**B**). Fractional clearance for the three different groups over time. 40 min after perfusion with HS the rats became anuric (**C**). 10 min after the perfusion the fractional clearance of albumin was increase in the rats perfused with HS, but not in the rats perfused with HO or NS (**D**). **P < 0.01, ***P < 0.001, error bars represent SEM.

To understand if the reduction of the ESL affected the GFR and fractional clearance of albumin functional measurements were performed. The pre-perfusion GFR was approximately 1.2 ml/min/g wet kidney weight in all animals (NS 1.21 ± 0.04, HO 1.14 ± 0.06, HS 1.18 ± 0.04, n = 6). The post-perfusion GFR fell to 0.6 ml/min/g wet weight in HO perfused rats and remained at this level during the observation period. In HS rats, the GFR was decreased to 0.1 ml/min/g wet weight 10 min post perfusion (P < 0.01 compared to HO, P < 0.001 compared to NS), and 40 min post perfusion these animals were anuric (Fig. 2A,B). GFR was unchanged in NS perfused rat kidneys throughout the observation period. Fractional clearance of albumin increased significantly in the HS rats 10 min post perfusion (P < 0.001 compared to both HS and HO, Fig. 2C,D) but remained normal for the NS rats and was only slightly elevated in the HO rats. These data show that loss of ESL increases the fractional clearance of albumin pinpointing its importance for a normal glomerular permselectivity.

### Identification of proteoglycan content of the endothelial cell surface layer in rats

To identify the eluted proteins from the ESL we performed mass spectrometry analysis and compared the findings from the different eluates. In total, 575 different proteins were identified in the eluates by label-free proteomics. Since the rat proteomics database contains a limited number of proteins (around 8000), a database for mouse (around 17,000 proteins) was added to the analysis, taking the homology between the two species into account. Using this database, 658 proteins were identified. In total, we identified 17 proteoglycans in our samples: tsukushin, lumican, decorin, biglycan, syndecan-4, glypican-1, protein AMBP, serglycin, agrin, chondroitin sulfate proteoglycan 4, amyloid-beta A4 protein, CD44 antigen, perlecan, podocan, glypican-4, collagen alpha-1 (XV) chain and collagen alpha-1 (XVIII) chain. Using the extended database search (mouse) we identified all the PGs found using the rat database, except for serglycin. Quantitative information about the identified PGs are presented in Table 2. Amyloid beta A4^17^ and CD44^18^ are not always linked to GAG chains so further investigation is needed to confirm their GAG content. As expected, PGs were more abundant in the HS eluate compared to HO and NS eluates since HS elutes highly negatively charged molecules.

**Table 2:** Proteoglycans identified in renal eluates from rats using the Swiss-prot rat and mouse database, respectively.

Accession	Description	MW [kDa]	Coverage [%]	# PSMs	# peptides quantified	Abundance NS	Abundance HO	Abundance HS	Ratio HS/HO
Q6QMY6	Tsukushin^a,b^	38.1	4	4	1	20,418	23,885	35,097	1.5
P51886	Lumican^a,b^	38.3	33	177	10	86,372,660	105,850,162	441,680,290	4.2
Q01129	Decorin^a,b^	39.8	14	17	5		503,440	4,310,444	8.6
P47853	Biglycan^a,b^	41.7	17	9	5	87,655	97,718	823,404	8.4
P34901	Syndecan-4^a,b^	21.9	27	43	4	6,663,690	16,561,848	27,852,454	1.7
P35053	Glypican-1^a,b^	61.7	20	9	8		78,216	1,242,848	15.9
Q64240	Protein AMBP^a,b^	38.8	41	231	12	242,822,968	170,135,637	206,468,772	1.2
P04917	Serglycin^a^	18.6	16	9	2	86,974	184,847	858,694	4.6
P25304	Agrin^a,b^	208.5	8	16	8	411,375	352,127	797,641	2.3
Q00657	Chondroitin sulfate proteoglycan 4^a,b^	251.8	7	16	12		6614	895,270	135.4
P08592	Amyloid-beta A4 protein^a,b^	86.6	10	19	5	182,002	1,339,443	1,719,534	1.3
P26051	CD44 antigen^a,b^	55.9	4	9	2	935,255	846,521	2,513,633	3.0
Q05793	Perlecan^b^	398	11	76	26	19,946,442	337,796,644	20,731,486	0.06
Q7TQ62	Podocan^b^	68.7	16	10	7	25,595		3,427,046	N/A
P51655	Glypican-4^b^	62.5	3	1	1			10,530	N/A
O35206	Collagen alpha-1 (XV) chain^b^	140.4	2	3	2	8,336,392	12,083,764	236,860	0.02
P39061	Collagen alpha-1 (XVIII) chain^b^	182.1	3	14	5	433,880	335,745	1,183,567	3.53

The reported abundances were calculated based on the peptides quantified, normalized to total amount of peptides.

^a^Identified searching the proteomics data against the Swiss-Prot rat database.

^b^Identified searching the proteomics data against the Swiss-Prot mouse database.

In order to understand which of the eluted proteins are important for matrix organization we used the Reactome web page for pathway analysis. We found that 25 of our identified proteins belong to the pathway extracellular matrix organization (Table S1), including PGs but also other important proteins for matrix formation like fibronectin and collagens. All identified proteins from the respective databases are found in appendix S1, S2.

### Identification of the glycosaminoglycan hyaluronan in renal eluates from rats

**Figure 3:**
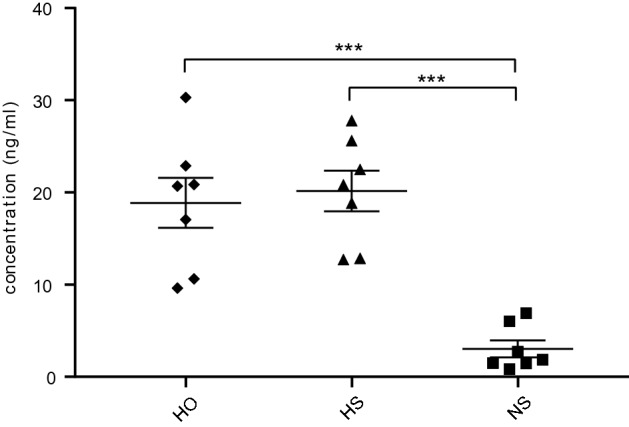
Concentration of hyaluronan in eluates. The concentration of hyaluronan in the eluates from the rats was measured after perfusion with physiological salt (NS), high osmolarity (HO) and high salt (HS) solution. Perfusing with HS or HO eluted similar amounts of hyaluronan, while NS eluted lower amounts. . ***P < 0.001, error bars represent SEM.

Hyaluronan has been thought to play a role in the permselectivity of the glomerular filtration barrier. It is a glycosaminoglycan that lacks a core protein (and is not anchored to the cell surface) and could not be identified using our mass spectrometry set up. We therefore determined the hyaluronan content using ELISA. The amount of hyaluronan in eluates from HS and HO rats was significantly higher than the amount of hyaluronan in eluates from NS perfused rats (p < 0.001, n = 7, Fig. 3).

### Proteoglycan and proteoglycan-related proteins in glomeruli from patients with diabetic kidney disease

A reduction of ESL has previously been shown in patients with DKD and our in vivo results show that loss of ESL and PGs leads to reduced GFR and proteinuria. Next we investigated whether the PGs identified in the ESL in vivo were expressed and altered in glomeruli from patients with DKD compared to healthy controls. We analyzed our previously published next generation sequencing (NGS) data set on micro-dissected glomeruli from patient biopsies^19^. Expression of LUM, GPC4, COL15A1, COL18A1 and CD44 were significantly upregulated while AGRN and DCN were downregulated (Table 3). In addition, we found six additional PGs to be significantly regulated in the patient glomeruli compared to control (Table S2). Several of these PGs had a similar, statistically significant regulation in a second transcriptomic data set of glomeruli from DKD patients^20^, Table S3. The enzymes involved in GAG chain synthesis and addition of the negatively charged sulfate groups were significantly downregulated (Table S4). In addition, we discovered enzymes and proteins involved in PG degradation to be significantly up-regulated (Table S4). Overall, this indicates alterations in PG and GAG chain turnover and decreased synthesis of GAG chains in glomeruli from DKD patients resulting in a decreased negative charge of the ESL, hence contributing to glomerular dysfunction and proteinuria.

**Table 3:** Significantly regulated proteoglycans in glomeruli from patients with diabetic kidney disease compared to control.

Gene symbol	Official name	Fold change (log2)	Fold change (unlogged)	p-value	p-value (adjusted)
LUM	Lumican	2.07	4.19	1.09E−08	5.48E−07
AGRN	Agrin	− 0.66	0.63	0.000119	0.0016753
DCN	Decorin	− 1.57	0.34	7.52E−20	9.41E−17
GPC4	Glypican 4	0.80	1.74	0.000843	0.0079477
COL15A1	Collagen type XV alpha 1 chain	1.62	3.08	3.58E−07	1.19E−05
COL18A1	Collagen type XVIII alpha 1 chain	0.58	1.50	0.000386	0.004271
CD44	CD44 molecule (Indian blood group)	1.11	2.16	1.58E−07	5.84E−06

### Localization of proteoglycans in the human glomerular capillary endothelium

**Figure 4:**
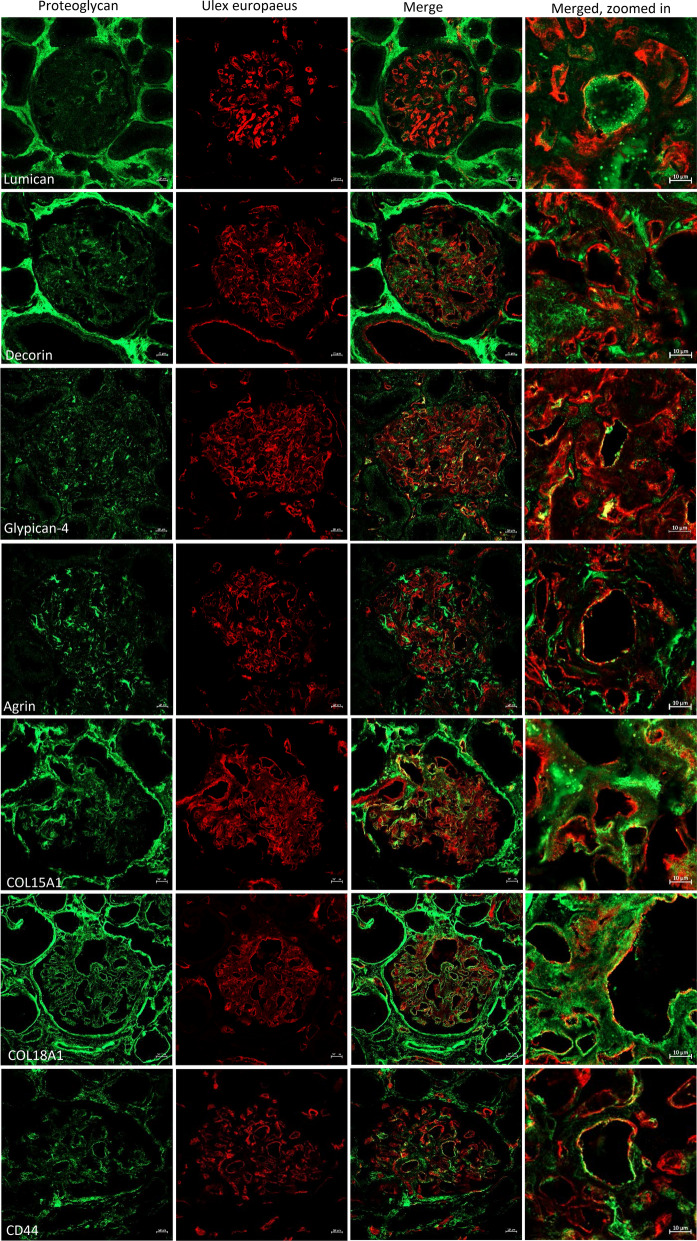
Expression of proteoglycans in the human glomerular endothelium. Immunofluorescence of proteoglycans and *Ulex europaeus* agglutinin I, a marker for endothelial cells (red), confirmed expression of (in green) lumican, decorin, glypican-4, agrin, collagen 15 alpha 1 chain, collagen 18 alpha 1 chain and CD44 in the glomerulus of human kidney sections. Co-localization is seen as yellow. Scale bars for pictures of full glomeruli represent 20 µm, zoomed in images 10 µm.

We performed co-localization experiments with immunofluorescence to determine if the PGs found in the eluted ESL in vivo and expressed and regulated in glomeruli from DKD patients are localized to the surface of the human glomerular endothelium. PGs were visualized in combination with the endothelial cell marker *Ulex europaeus* agglutinin I on tissue sections from healthy human kidneys. There was a clear co-localization with the endothelial marker for expression of lumican, glypican-4, agrin, collagen alpha-1 (XVIII) chain and CD44 while there was a lower degree of co-localization for decorin and collagen alpha-1 (XV) chain (Fig. 4).

### Expression of proteoglycans in primary human glomerular endothelial cells in diabetic milieu

**Figure 5:**
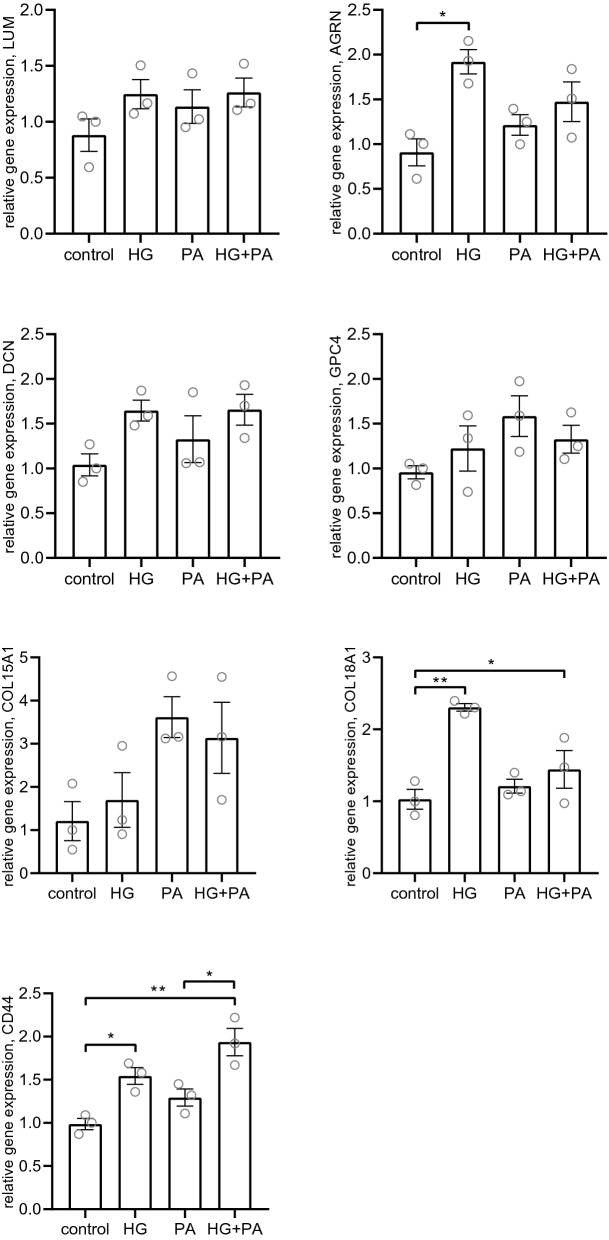
Gene expression of proteoglycans in human glomerular endothelial cells in vitro. Relative gene expression of the proteoglycans; LUM, AGRN, DCN, GPC4, COL15A1, COL18A1 and CD44 after treatment of human glomerular endothelial cells with high glucose (HG), palmitate (100 µM) bound to HSA (PA) or a combination of both (HG+PA) for 24 h. *P < 0.05, **P < 0.01, error bars represent SEM.

**Figure 6:**
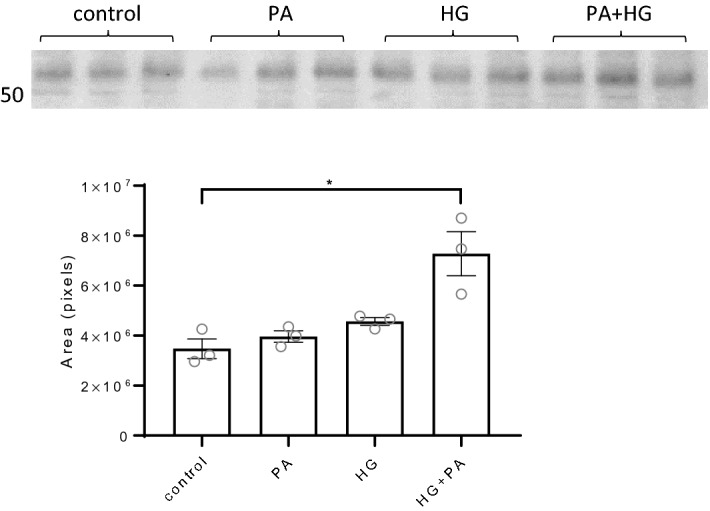
Protein expression of lumican in human glomerular endothelial cells in vitro. Western blot of lumican expression in cells after treatment of human glomerular endothelial cells with high glucose (HG), palmitate (100 µM) bound to HSA (PA) or a combination of both (HG+PA)for 24 h. The band corresponding to lumican was analyzed for protein abundance. The blot presented is a cropped version without ladder and the full blots are presented in Supplementary Figure 1. *P < 0.05, error bars represent SEM.

Since the DKD cohort revealed significant alterations in PG expression in glomeruli we wanted to investigate how a diabetic milieu could affect PG expression in human glomerular endothelial cells (hGEC). All investigated genes (LUM, DEC, AGRN, GPC4, COL15A1, COL18A1 and CD44) were expressed by the cells, but there was lower expression of GPC4 and COL15A1. The cells were treated with high glucose (HG) or palmitate (PA) bound to human serum albumin (HSA) or a combination of the two (HG + PA) for 24 h to investigate alterations in the PG gene expression of LUM, DEC, AGRN, GPC4, COL15A1, COL18A1 and CD44. The expression of the two small leucine-rich PGs (SLRPs) LUM and DCN was only slightly affected by the treatment compared to control. AGRN had a significantly increased expression when treated with HG (P < 0.05), but not with PA or PA + HG. COL18A1 had significantly increased expression when treated with HG (P < 0.01) and HG + PA (P < 0.05) compared to untreated controls. This pattern was also seen for CD44, with a significant increase in the expression when treated with HG (P < 0.05) and HG + PA (P < 0.01). There was no significant differences found for COL15A1 and GPC4 (Fig. 5). Protein expression of lumican revealed significant upregulation of lumican in cells treated with HG + PA (P < 0.05) (Fig. 6). Overall, this indicate that PG expression in glomerular endothelial cells is affected in diabetic conditions.

## Discussion

PGs are a family of proteins characterized by a core protein covalently binding one or more GAG chains contributing to the charge selectivity of the glomerular filtration barrier^21^. PGs are important for structure and anchoring of the ESL and storage of growth factors and other signaling molecules^22^. It is known that patients with diabetes mellitus have a reduction in endothelial glycocalyx thickness and charge selectivity of the glomerulus^13,14^ that can contribute to proteinuria and loss of GFR. In this study, we investigated specific PG content in rats in vivo by eluting charged components of the ESL from the glomerular capillaries. After identifying the PG content of the ESL we went on to explore the gene expression in the humans by analyzing the data retrieved from glomeruli from patients with DKD using a NGS DKD cohort previously published by our group^19^. Finally, we investigated how a diabetic milieu affect the glomerular endothelial cell PG production in vitro.

To determine the composition of the ESL, we perfused rats in vivo with high salt (HS) to elute the negatively charged molecules in the ESL. As high salt alters the osmolality of the solution, we added a high osmolality (HO) group and a physiological salt solution (NS) as controls. The eluates were analyzed using mass spectrometry and the renal function and morphology after perfusion was investigated. HS perfusion resulted in a significant decrease of the glomerular ESL thickness and the rats developed albuminuria with a decrease of GFR with over 85%.

Exposure to HO did not reduce the thickness of the ESL and did not increase the fractional clearance of albumin but reduced the GFR to around 50%. The reason for the profound effect on GFR in HS and HO rats could not be explained by morphological alterations of the glomerular structures, since they were intact as analyzed by TEM. In an earlier paper, we used a shorter (10–15 s) flush with HS to elute the ESL, leading to a reduction in GFR and increased fractional clearance of albumin with recovery after 20 min. The only PG detected at that time was lumican^2^, indicating that a longer elution time is needed to elute larger molecules from the ESL. In the present study, applying a longer elution time 17 PGs were identified in the ESL in combination with an even more pronounced increase in albuminuria and reduction of GFR. This allows us to draw the conclusion that the integrity and function of the ESL is highly dependent on its PG and GAG content.

When analyzing the glomerular data from the DKD patient cohort we discovered that 7 of the 17 PGs determined to be a part of the ESL in vivo, were significantly regulated compared to healthy controls at the gene level. LUM, GPC4, CD44, COL15A1 and COL18A1 were upregulated while AGRN and DCN was downregulated. To confirm the localization of these proteins to the glomerular endothelium, immunohistochemical analysis of human kidney sections were performed. This revealed that lumican, glypican-4, agrin, collagen alpha-1 (XVIII) chain and CD44 co-localized with the glomerular endothelial marker while there was a lower abundance of co-localization for decorin and collagen alpha-1 (XV) chain. In vitro experiments confirmed that the gene expression of the seven PGs was detectable in hGECs.

Culturing hGECs in a diabetic milieu consisting of high glucose and palmitate confirmed that PG expression is affected in this setting, mainly through up-regulation, analogously to what was seen in the human DKD setting. As DKD is known to be associated with a reduced ESL and proteinuria, we expected the levels of PGs to be reduced both in the DKD cohort and in the in vitro setting. However, it is not only the PG core protein expression that determine the integrity and function of the ESL. It is likely that the GAG chain content also is important for their function and structure. Analysis of the DKD cohort revealed a decrease in all the detected enzymes involved in GAG chain synthesis in combination with an increase in most of the proteins involved in PG degradation. This implies that the reduction in ESL in DKD may not only be a result of loss of core proteins but also of negatively charged GAG chain content and an increased turnover of PGs.

It has been suggested that hyaluronan, an anionic, non-sulfated GAG with a massive chain length and one of the main components of extracellular matrix^23^ is vital for ESL integrity. Loss of hyaluronan has been observed in patients with DKD and endothelium-specific knock out of the enzyme Has2 (one of the three enzymes involved in the production of hyaluronan) in mice led to substantial loss of their glycocalyx structure^24^. In addition, knock out of the enzyme hyaluronidase, an enzyme that cleaves hyaluronan, prevented albuminuria in a mouse model of type I diabetes^25^. However, we found equal amounts of hyaluronan in the eluates from HS and HO rats indicating that the increased fractional clearance of albumin in the HS rats is not likely to be due to loss of hyaluronan in contrary to the other studies above. Neither did we find significant changes in the gene expression of any of the enzymes that are needed to synthesize hyaluronan (HAS1, 2 or 3) in our NGS DKD cohort. However, the enzymatic activity could still be affected and this was not investigated.

When further investigating the role of the individual PGs lumican, a small leucine-rich PG (SLRP), was the most up-regulated PG in both the DKD cohorts investigated. Lumican has previously been identified in the human glomerular endothelium^2,26^ while decorin (another SLRP) has been found mainly in sclerotic areas of the glomerulus^27,28^. In line with the biopsy data, lumican protein expression was increased when treating hGECs with PA and HG. Lumican has been suggested as a plasma biomarker for DKD when exploring the plasma glycoproteome in diabetic controls and DKD patients^29^. It is also involved in inflammation and can affect the innate immune system as well as the TGFβ signaling pathway, which is common to other members of the SLRPs^6^. Furthermore lumican is involved in collagen fibril assembly, and thus important for the function of extracellular matrixes^30^. Our results confirm that lumican has an important role in the ESL and in DKD, likely along with some of the other glomerular ESL proteoglycans that were identified in this study. To be able to fully understand the role of PGs in the glomerular ESL further analysis of protein expression and function in DKD patient populations are needed.

In conclusion, this study supports the role of the ESL as an important contributor to the charge selective properties of the glomerular barrier. It highlights the role of the ESL in preventing albumin from passing over the filtration barrier, already in the lumen of the glomerular capillaries. Alteration of the composition and amount of PGs in the ESL leads to a reduced thickness of the ESL but may also lead to disturbances in local signaling events. This is due to the important role of PGs in regulating and harboring signaling molecules and growth factors. We speculate that prevention of loss of PGs or enhancement of their reconstitution in the glomerular ESL in DKD could prevent albuminuria and improve renal function.

## Methods

### Animals and experimental design

Experiments were performed on Female Sprague–Dawley rats (Harlan, Horst, Netherlands) weighing 230 to 270 g, n = 17. Animals had free access to food and water. All surgical procedures took place under isoflurane (Isoba vet, Schering-Plough Animal Health) anesthesia. The animals were randomly divided into 3 groups, high salt (HS, n = 7), high osmolarity (HO, n = 5) and physiological salt (NS, n = 5). The Gothenburg animal ethics committee approved all experimental procedures; all experiments were performed in compliance with the ARRIVE guidelines and relevant regulations.

### Perfusion solutions

Three different perfusion solutions, all based on Tyrode’s solution, were used. The composition was as follows (in mM); normal salt (NS, 0.15 M NaCl) 148.1 Na and 133.5 Cl, high salt (HS, 1 M NaCl) (in mM) 1032.65 Na and 1018.05 Cl and the high osmolality (HO) 148.1 Na, 133.5 Cl, and 692.0 mannitol. All solutions contained 25.04 HCO_3_, 0.49 H_2_PO_4_, 0.83 Mg, 4.29 K, 2.50 Ca and 5.60 glucose. They were protected from light, bubbled with 5% CO_2_ in O_2_ and used at 37 °C. The pH was maintained at 7.4 throughout the experiment.

### Renal morphology

Directly after the perfusion of the kidneys with either NS, HS or HO, 1.5 ml of intralipid solution was injected. The intralipid solution was prepared from Intralipid (Fresenius Kabi AB, Uppsala, Sweden) as described before^2^. The left renal artery and vein was clamped and the kidney was fixed by subcapsular injection of Karnovsky’s fixative and sliced as described earlier^5^.

### Transmission electron microscopy

Transmission electron microscopy was used to assess the thickness of the ESL and the ultrastructure of the glomerular filtration barrier after perfusion as previously published^2,31^. Micrographs of glomerular capillaries at a magnification of 8000 were acquired from five animals in each group, giving a total of 378 unique glomerular capillaries (102 from NS, 143 from HS and 133 from HO rats). Morphological measurements were performed in a blinded fashion using BioPix iQ 2.2.1 (BioPix AB, Göteborg, Sweden).

### Collection of renal eluates

Preparation for collection of renal eluates was performed as previously described^2^. The kidneys were perfused with 12 ml of perfusion solution with NS, HS or HO during 3 min using a syringe pump (AgnTho's AB, Lindingö, Sweden) and finally rinsed with 1 ml of 0.15 M NaCl. The amount of albumin and ^51^Cr-EDTA in blood and urine samples, as well as glomerular filtration rate, was estimated as previously^2^.

### Processing of renal eluates for mass spectrometry-based proteomics

Individual renal eluate replicates were centrifuged and a pooled sample was generated representing each of the perfusion treatments HS (n = 7), HO (n = 5) and NS (n = 5). Each replicate sample contributed with the same amount of > 50 kDa molecular weight (Mw) proteins. Peptides and proteins were identified using proteome discoverer with Mascot (Thermo Scientific, Waltham, MA) matching experimental data against the *Rattus norwegicus* and *Mus musculus* Swiss-Prot protein databases. The relative protein abundances reported are based on the sum of normalized abundances of unique peptides corrected for variance in the global intensities of all features. The sample preparation, mass spectrometry and proteomics methods including the data analysis are explained in detail in Appendix S3.

### Reactome pathway database

A list of identified eluted proteins was analyzed with the Reactome web page for pathway analysis (https://reactome.org/)^32^. Proteins identified as regulated in the proteomics analysis were searched against the *Rattus norvegicus* Uniprot database and filtered by statistical significance (p < 0.05).

### Assessment of hyaluronan in renal eluates

The amount of hyaluronan present in eluates from rat kidneys perfused with NS, HS and HO was determined by using the Hyaluronan Enzyme-Linked Immunosorbent Assay kit K-1200 (Echelon Biosciences Inc., Salt Lake City, UT, USA) according to manufacturer’s protocol.

### Immunofluorescence

Cryosections of nephrectomized healthy human kidney tissue was used for immunofluoresence. The antibodies used were; anti-lumican (R&D Systems, Minneapolis, MN), anti-decorin (Thermo Scientific), anti-glypican-4 (Thermo Scientific), anti-CD44 (Thermo Scientific) anti-agrin (Abcam), anti-collagen alpha-1 (XVIII) chain and collagen alpha-1 (XV) chain (Atlas Antibodies, Stockholm, Sweden). For endothelial co-localization the endothelial-specific lectin rhodamine *Ulex Europeaus* agglutinin (Vector Laboratories, Burlingame, CA) was used and the sections were analyzed with a confocal microscope, Zeiss (Zeiss, Oberkochen, Germany).

### Human diabetic kidney disease next generation sequencing cohort

RNA seq data from glomeruli from biopsy material previously published was used in this study to examine the expression of PGs and related genes^19^. 19 patients with DKD were included, median (range) age: 61 (30–85), DKD stages 1–4. 20 living kidney donors were used as control, median (range) age: 56 (30–70) years. The local ethics committees in Sweden (Stockholm and Gothenburg) approved the study.

### Human glomerular endothelial cells treated with high glucose and palmitate

Primary human glomerular endothelial cells (Cell Systems, Kirkland, WA) were cultured in Complete Medium as described by the manufacturer. Palmitic acid was prepared in NaCl 150 mM pH 7.4 solution, and conjugated with human serum albumin (HSA) in a 6:1 molar ratio palmitate/HSA for 1 h at 37 °C. Cells were starved in medium containing 0.5% FBS and no culture boost for 24 h prior to stimulation with 30 mM high glucose/HSA, palmitate/HSA (Sigma-Aldrich, Saint Louis, MO) 100 µM or a combination of the both for 24 h. Normal glucose (5 mM) was used as control. RNA was extracted and purified with RNeasy Mini Kit (Qiagen, Hilden, Germany). cDNA was generated using High Capacity RNA-to-cDNA kit (Thermo Scientific). Quantitative PCR was performed using Taqman probes (Thermo Scientific) with GAPDH as housekeeping gene on the QuantStudio 7 Pro System (Thermo Scientific). All assays were performed in biological triplicates and technical quadruplicates.

For protein analysis the cells were harvested with lysis buffer (Triton X-100 1% Tris–HCl 50 mM, NaCl 150 mM, pH 7.5) with phosphatase and protease inhibitors (Sigma Aldrich) and protein concentration was determined using Pierce BCA protein assay kit (Thermo Fisher Scientific). Western blot was run using Mini-Protean TGX Stain free Gel 4–15%. Lumican was visualized using an anti-lumican antibody (R&D Systems). Images were acquired with a ChemiDoc Touch Imager (Bio-Rad). Relative quantification of lumican was done using the Bio-Rad V3 Western Workflow by normalizing the band intensities to the total lane volume.

### Statistics

Graphpad Prism v.8.3.0 (Graphpad Software, San Diego, CA) was used for all analysis. One-way ANOVA with multiple comparisons using Sidak’s test were used for analysis of normally distributed data. Non-normally distributed data were analyzed using Kruskal–Wallis test with multiple comparisons using Dunn’s test. P < 0.05 was considered statistically significant. Error bars represent SEM unless stated otherwise.

## Supplementary Information

Supplementary Information.
