# Supplementary material for: Proteoglycans contribute to the functional integrity of the glomerular endothelial cell surface layer and are regulated in diabetic kidney disease
Source: Sci Rep. 2021 Apr 19;11:8487. doi: 10.1038/s41598-021-87753-3 (PMC8055884; doi:10.1038/s41598-021-87753-3)
Supplement: Supplementary file 1 — Supplementary Information. [file 41598_2021_87753_MOESM1_ESM.docx]

**SUPPLEMENTARY MATERIAL**

**Proteoglycans contribute to the functional integrity of the glomerular endothelial cell surface layer and are regulated in diabetic kidney disease**

Alina Khramova, MS, Roberto Boi, PhD, Vincent Fridén, PhD, Anna Björnson Granqvist, PhD, Ulf Nilsson, PhD^,^ Olav Tenstad, Professor, Eystein Oveland, PhD, Börje Haraldsson, MD, Professor, Kerstin Ebefors, PhD, Jenny Nyström, Professor

Supplementary tables, page 2-4

Supplementary figure, page 5

Supplementary appendix 1 – all proteins detected using the Swiss-prot rat data base, page 6-46

Supplementary appendix 2 – all proteins detected using the Swiss-prot mouse data base, page 46-90

Supplementary appendix 3 – supplementary methods, page 91-93

**Supplementary tables**

| Supplementary table 1. Proteins identified in the rat eluates belonging to the Reactome pathway Extracellular matrix organization | | | | | | | | |
| --- | --- | --- | --- | --- | --- | --- | --- | --- |
| Accession | Description | MW [kDa] | Coverage [%] | # PSMs | # Peptides quantified | Abundance  HS | Abundance  HO | Abundance  HS |
| P06238 | Alpha-2-macroglobulin | 163.7 | 25 | 249 | 27 | 52778096 | 47435754 | 13801928 |
| P29534 | Vascular cell adhesion protein 1 | 81.2 | 13 | 16 | 6 | 1344367 | 1033779 | 1221146 |
| P34901 | Syndecan-4 | 21.9 | 27 | 43 | 4 | 6663690 | 16561848 | 27852454 |
| Q9R0T4 | Cadherin-1 | 98.7 | 17 | 27 | 6 | 174205 | 611778 | 5557864 |
| P04937 | Fibronectin | 272.3 | 35 | 538 | 55 | 113930760 | 25697190 | 153515731 |
| Q01177 | Plasminogen | 90.5 | 58 | 247 | 33 | 364849972 | 310761308 | 291468458 |
| P51886 | Lumican | 38.3 | 33 | 177 | 10 | 86372660 | 105850162 | 441680290 |
| O08775 | Vascular endothelial growth factor receptor 2 | 150.3 | 2 | 2 | 2 | 58754 |  |  |
| Q01129 | Decorin | 39.8 | 14 | 17 | 5 |  | 503440 | 4310444 |
| P14480 | Fibrinogen beta chain | 54.2 | 67 | 442 | 27 | 110491158 | 3129253 | 486638587 |
| P02767 | Transthyretin | 15.7 | 56 | 84 | 6 | 148264770 | 157284791 | 139660963 |
| P25304 | Agrin | 208.5 | 8 | 16 | 8 | 411375 | 352127 | 797641 |
| P47853 | Biglycan | 41.7 | 17 | 9 | 5 | 87655 | 97718 | 823404 |
| P02466 | Collagen alpha-2(I) chain | 129.5 | 2 | 7 | 2 | 1434581 |  | 631199 |
| P04785 | Protein disulfide-isomerase | 56.9 | 35 | 30 | 13 | 3569909 | 1485610 | 16017389 |
| P97571 | Calpain-1 catalytic subunit | 82.1 | 19 | 35 | 11 |  | 1063236 | 2080546 |
| Q64537 | Calpain small subunit 1 | 28.6 | 13 | 4 | 2 | 381653 | 1981696 | 1197581 |
| P02680 | Fibrinogen gamma chain | 50.6 | 45 | 358 | 17 | 131975073 | 5430348 | 603464056 |
| P26051 | CD44 antigen | 55.9 | 4 | 9 | 2 | 935255 | 846521 | 2513633 |
| Q62598 | Dentin sialophosphoprotein | 70.1 | 2 | 3 | 1 |  |  | 59302 |
| P17246 | Transforming growth factor beta-1 proprotein | 44.3 | 3 | 1 | 1 |  |  | 77231 |
| P08592 | Amyloid-beta A4 protein | 86.6 | 10 | 19 | 5 | 182002 | 1339443 | 1719534 |
| P14272 | Plasma kallikrein | 71.2 | 38 | 88 | 15 | 47329682 | 44529700 | 45192721 |
| P06399 | Fibrinogen alpha chain | 86.6 | 30 | 261 | 21 | 67283988 | 13138784 | 338039327 |
| P02454 | Collagen alpha-1(I) chain | 137.9 | 7 | 16 | 7 | 4453848 | 450246 | 81690 |
| The reported abundances were calculated based on the peptides quantified, normalized to total amount of peptides. | | | | | | | | |

| Supplementary table 2. Additionally significantly regulated proteoglycans in glomeruli from patients with diabetic kidney disease compared to control | | | | | |
| --- | --- | --- | --- | --- | --- |
| Gene symbol | Official name | Foldchange (log2) | Foldchange (unlogged) | p-value | p-value (adjusted) |
| VCAN | versican | 1.80 | 3.47 | 4.98E-07 | 1.60E-05 |
| TGFBR3 | transforming growth factor beta receptor 3 | -1.59 | 0.33 | 1.79E-18 | 1.23E-15 |
| FMOD | fibromodulin | 1.26 | 2.39 | 3.69E-05 | 0.000633 |
| PRELP | proline and arginine rich end leucine rich repeat protein | 1.00 | 2.01 | 2.65E-05 | 0.00049 |
| SPOCK1 | SPARC (osteonectin), cwcv and kazal like domains proteoglycan 1 | -0.97 | 0.51 | 0.000339 | 0.003852 |
| SPOCK2 | SPARC (osteonectin), cwcv and kazal like domains proteoglycan 2 | -1.33 | 0.40 | 2.62E-07 | 9.03E-06 |
| * this protein has been found to sometimes have GAG chains attached to its core | | | | | |

| Supplementary table 3. Significantly regulated proteoglycans in glomerluli from DKD pateints (in data set from Woroniecka et al) | | |
| --- | --- | --- |
| Probe Set ID | gene symbol | Fold change |
| 201744_s_at | LUM | 5.19 |
| 201893_x_at | DCN | -2.48 |
| 204619_s_at | VCAN | 2.42 |
| 204731_at | TGFBR3 | -4.76 |
| 202709_at | FMOD | 1.67 |
| 202363_at | SPOCK1 | -6.61 |
| 202523_s_at | SPOCK2 | -2.22 |
| 202524_s_at | SPOCK2 | -4.29 |
| 204489_s_at | CD44 | 1.81 |
| 204490_s_at | CD44 | 1.56 |
| 212063_at | CD44 | 2.66 |
| P-value less than 0.05 for all genes | | |

| Supplementary table 4. Significantly regulated genes of proteins and enzymes involved in proteoglycan GAG chanin synthesis and breakdown in glomeruli from patients with diabetic kidney disease compared to control | | | | | |
| --- | --- | --- | --- | --- | --- |
| Gene symbol | Official name | Foldchange (log2) | Foldchange (unlogged) | p-value | p-value (adjusted) |
| *Enzymes involved in GAG synthesis* | |  |  |  |  |
| CHSY1 | chondroitin sulfate synthase 1 | -0.71 | 0.61 | 4.82E-05 | 0.0007887 |
| HS3ST3A1 | heparan sulfate-glucosamine 3-sulfotransferase 3A1 | -1.11 | 0.46 | 7.84E-06 | 0.0001711 |
| HS3ST3B1 | heparan sulfate-glucosamine 3-sulfotransferase 3B1 | -0.97 | 0.51 | 5.30E-05 | 0.0008531 |
| XYLT1 | xylosyltransferase 1 | -1.06 | 0.48 | 9.73E-05 | 0.0014102 |
| CHST15 | carbohydrate sulfotransferase 15 | 1.03 | 2.04 | 0.000134 | 0.001843 |
| CHST14 | carbohydrate sulfotransferase 14 | -1.09 | 0.47 | 2.41E-08 | 1.12E-06 |
| CHST1 | carbohydrate sulfotransferase 1 | -1.35 | 0.39 | 2.51E-06 | 6.52E-05 |
| MGAT5 | alpha-1,6-mannosylglycoprotein 6-beta-N-acetylglucosaminyltransferase | -1.13 | 0.46 | 3.35E-06 | 8.41E-05 |
| *Proteins involved in PG degradation* | |  |  |  |  |
| GNS | glucosamine (N-acetyl)-6-sulfatase | 0.58 | 1.50 | 2.70E-05 | 0.0004972 |
| ADAM9 | ADAM metallopeptidase domain 9 | 1.01 | 2.01 | 6.44E-05 | 0.0010039 |
| ADAMTS9 | ADAM metallopeptidase with thrombospondin type 1 motif 9 | -1.05 | 0.48 | 4.00E-12 | 4.62E-10 |
| CTSA | cathepsin A | 0.70 | 1.63 | 6.08E-06 | 0.0001383 |
| CTSC | cathepsin C | 0.86 | 1.82 | 0.000194 | 0.0024923 |
| CTSD | cathepsin D | 0.58 | 1.50 | 3.51E-06 | 8.71E-05 |
| CTSS | cathepsin S | 0.92 | 1.89 | 0.0002 | 0.0025463 |
| MMP2 | matrix metallopeptidase 2 | 1.29 | 2.44 | 6.05E-07 | 1.90E-05 |
| MMP7 | matrix metallopeptidase 7 | 1.93 | 3.82 | 7.31E-11 | 6.79E-09 |
| MMP14 | matrix metallopeptidase 14 | 0.83 | 1.78 | 0.000302 | 0.0035171 |
| MMP25 | matrix metallopeptidase 25 | -1.20 | 0.44 | 0.000678 | 0.0066416 |
| MMP28 | matrix metallopeptidase 28 | -1.86 | 0.28 | 4.09E-13 | 6.62E-11 |
| MMP24 | matrix metallopeptidase 24 | 1.16 | 2.24 | 0.000218 | 0.0027158 |

**Supplementary Figures**


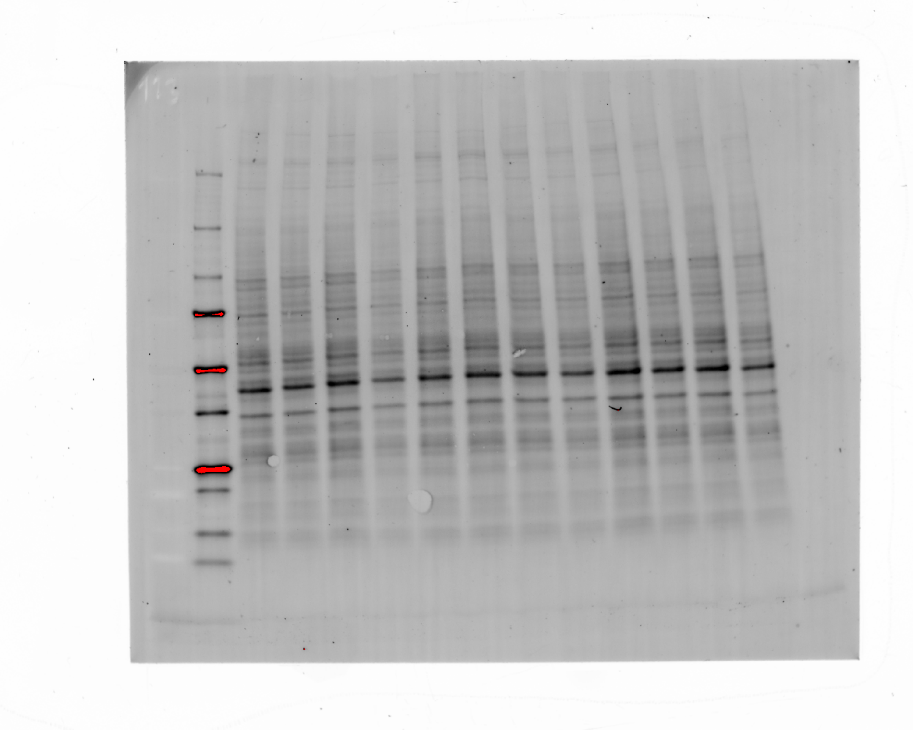
A B


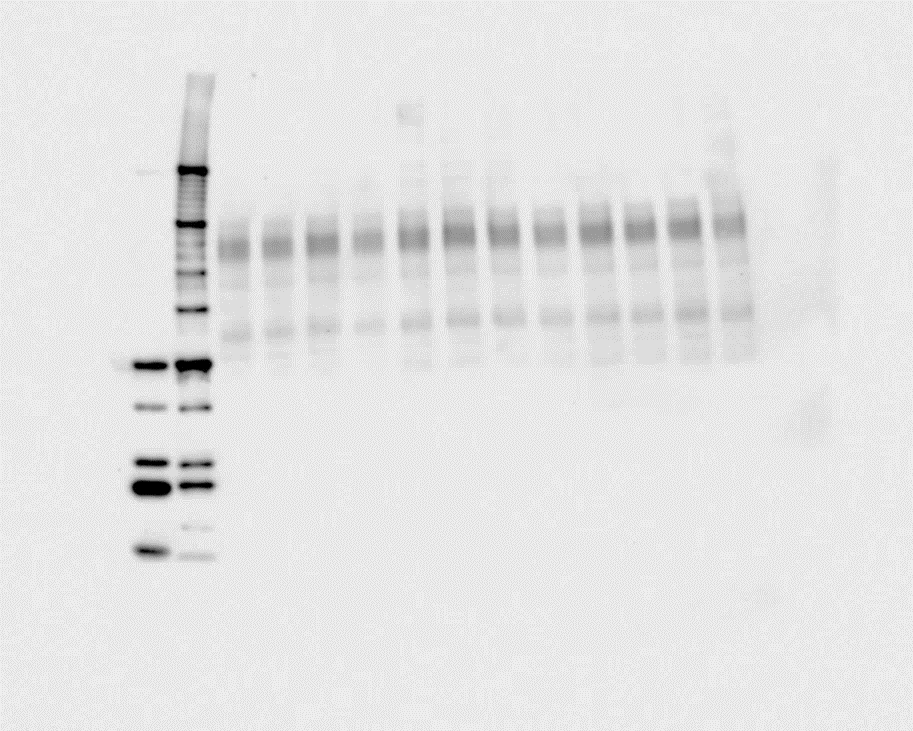


Control

PA

HG

PA+HG

200

150

75

50

100

**Supplementary figure 1**. Full blot for lumican. Red box corresponds to figure 6 (A). Total protein blot used for normalization for the lumican expression (B).

**Supplementary appendix 1.**

| Proteins identified in renal eluates from rats using the Swiss-prot rat database | | | | | | | | |
| --- | --- | --- | --- | --- | --- | --- | --- | --- |
| Accession | Description | MW [kDa] | Coverage [%] | # PSMs | # Peptides quantified | Abundances (Normalized): HO: Sample | Abundances (Normalized): NS: Sample | Abundances (Normalized): HS: Sample |
| Q32Q05 | Ubiquitin thioesterase OTU1 OS=Rattus norvegicus OX=10116 GN=Yod1 PE=2 SV=1 | 37.4 | 17 | 13 | 5 | 610672.3907 | 39849.57408 |  |
| Q6P0K8 | Junction plakoglobin OS=Rattus norvegicus OX=10116 GN=Jup PE=1 SV=1 | 81.7 | 7 | 5 | 5 |  | 203024.0537 |  |
| P30836 | L-selectin OS=Rattus norvegicus OX=10116 GN=Sell PE=2 SV=1 | 42.4 | 7 | 5 | 3 | 228434.395 | 311977.615 |  |
| P21743 | Insulin-like growth factor-binding protein 1 OS=Rattus norvegicus OX=10116 GN=Igfbp1 PE=1 SV=2 | 29.7 | 14 | 2 | 2 |  | 184675.7453 |  |
| Q9R0D6 | Transcobalamin-2 OS=Rattus norvegicus OX=10116 GN=Tcn2 PE=2 SV=1 | 47.4 | 4 | 3 | 1 |  | 76347.29423 |  |
| O08775 | Vascular endothelial growth factor receptor 2 OS=Rattus norvegicus OX=10116 GN=Kdr PE=1 SV=1 | 150.3 | 2 | 2 | 2 |  | 58753.89911 |  |
| Q6W3B0 | Desmoglein-4 OS=Rattus norvegicus OX=10116 GN=Dsg4 PE=1 SV=1 | 114.3 | 3 | 3 | 2 |  | 93786.77419 |  |
| Q91ZT1 | Vascular endothelial growth factor receptor 3 OS=Rattus norvegicus OX=10116 GN=Flt4 PE=1 SV=1 | 153.2 | 1 | 4 | 1 | 149509.4767 | 244925.8166 |  |
| Q6B345 | Protein S100-A11 OS=Rattus norvegicus OX=10116 GN=S100a11 PE=3 SV=1 | 11.1 | 11 | 1 | 1 |  | 15467.19709 |  |
| Q6AYK3 | Inositol-3-phosphate synthase 1 OS=Rattus norvegicus OX=10116 GN=Isyna1 PE=3 SV=2 | 60.8 | 1 | 1 | 1 | 61110.07058 | 18561.8345 |  |
| P54287 | Voltage-dependent L-type calcium channel subunit beta-3 OS=Rattus norvegicus OX=10116 GN=Cacnb3 PE=1 SV=1 | 54.5 | 2 | 1 | 1 | 7191.498317 | 7267.824807 |  |
| P07867 | Hepatic triacylglycerol lipase OS=Rattus norvegicus OX=10116 GN=Lipc PE=2 SV=2 | 55.6 | 3 | 2 | 1 |  | 439273.9413 |  |
| Q6IG00 | Keratin. type II cytoskeletal 4 OS=Rattus norvegicus OX=10116 GN=Krt4 PE=3 SV=1 | 57.6 | 8 | 32 | 3 | 136367162 | 171306426.9 | 1322472.258 |
| P47967 | Galectin-5 OS=Rattus norvegicus OX=10116 GN=Lgals5 PE=1 SV=2 | 16.2 | 24 | 17 | 3 | 133482.7183 | 129600.8747 | 24785.67969 |
| Q4VSI4 | Ubiquitin carboxyl-terminal hydrolase 7 OS=Rattus norvegicus OX=10116 GN=Usp7 PE=1 SV=1 | 128.3 | 7 | 4 | 4 | 289628.6173 | 276217.7099 | 230545.2422 |
| Q5FVM4 | Non-POU domain-containing octamer-binding protein OS=Rattus norvegicus OX=10116 GN=Nono PE=1 SV=3 | 54.9 | 11 | 3 | 1 | 73186703.19 | 37001915.05 | 8868129 |
| P08650 | Complement C5 (Fragment) OS=Rattus norvegicus OX=10116 GN=C5 PE=1 SV=2 | 9 | 25 | 6 | 2 | 1384889.328 | 1260348.292 | 264057.2188 |
| P49744 | Thrombospondin-4 OS=Rattus norvegicus OX=10116 GN=Thbs4 PE=1 SV=1 | 108.1 | 4 | 9 | 3 | 170453.2963 | 397551.6734 | 56893.53516 |
| Q5M7T9 | Threonine synthase-like 2 OS=Rattus norvegicus OX=10116 GN=Thnsl2 PE=2 SV=1 | 54.1 | 15 | 12 | 4 | 715689.7576 | 738958.3539 | 5572460.49 |
| Q99PD6 | Transforming growth factor beta-1-induced transcript 1 protein OS=Rattus norvegicus OX=10116 GN=Tgfb1i1 PE=1 SV=2 | 50.1 | 5 | 5 | 2 | 5147.399769 | 560582.7925 | 83435.1543 |
| Q00715 | Histone H2B type 1 OS=Rattus norvegicus OX=10116 PE=1 SV=2 | 14 | 19 | 4 | 2 | 473731.1244 | 875363.7563 | 327356.125 |
| P58775 | Tropomyosin beta chain OS=Rattus norvegicus OX=10116 GN=Tpm2 PE=1 SV=1 | 32.8 | 18 | 42 | 1 | 157903363.1 | 8728248.81 | 3766499.75 |
| P48199 | C-reactive protein OS=Rattus norvegicus OX=10116 GN=Crp PE=1 SV=1 | 25.5 | 42 | 986 | 8 | 480359150.6 | 728032683.7 | 435359583.4 |
| P15429 | Beta-enolase OS=Rattus norvegicus OX=10116 GN=Eno3 PE=1 SV=3 | 47 | 19 | 10 | 2 | 538226.7583 | 1244787.431 | 710455.5625 |
| P15083 | Polymeric immunoglobulin receptor OS=Rattus norvegicus OX=10116 GN=Pigr PE=1 SV=1 | 84.7 | 25 | 185 | 16 | 14867089.69 | 37122242.91 | 25887697.14 |
| P14630 | Apolipoprotein M OS=Rattus norvegicus OX=10116 GN=Apom PE=1 SV=2 | 21.5 | 23 | 16 | 3 | 2209335.433 | 1022403.528 | 647199.9082 |
| P10111 | Peptidyl-prolyl cis-trans isomerase A OS=Rattus norvegicus OX=10116 GN=Ppia PE=1 SV=2 | 17.9 | 46 | 17 | 7 | 1041921.494 | 759598.7834 | 2362071.09 |
| P09006 | Serine protease inhibitor A3N OS=Rattus norvegicus OX=10116 GN=Serpina3n PE=1 SV=3 | 46.6 | 58 | 158 | 19 | 303753005.4 | 397030166.1 | 317060819.5 |
| Q9EQV9 | Carboxypeptidase B2 OS=Rattus norvegicus OX=10116 GN=Cpb2 PE=2 SV=1 | 48.8 | 22 | 36 | 8 | 21930314.47 | 26475461.6 | 14919152.23 |
| Q63556 | Serine protease inhibitor A3M (Fragment) OS=Rattus norvegicus OX=10116 GN=Serpina3m PE=2 SV=1 | 46.1 | 46 | 115 | 14 | 103204011.6 | 91466471.42 | 68742146.5 |
| Q5M8C6 | Fibrinogen-like protein 1 OS=Rattus norvegicus OX=10116 GN=Fgl1 PE=2 SV=1 | 36.5 | 6 | 3 | 2 |  | 107176.9682 | 50571.38672 |
| Q64240 | Protein AMBP OS=Rattus norvegicus OX=10116 GN=Ambp PE=1 SV=1 | 38.8 | 41 | 231 | 12 | 170135637.3 | 242822967.6 | 206468771.7 |
| B0BN18 | Prefoldin subunit 2 OS=Rattus norvegicus OX=10116 GN=Pfdn2 PE=2 SV=1 | 16.6 | 17 | 3 | 2 | 1620101.74 | 945931.8647 | 1403929.625 |
| P07151 | Beta-2-microglobulin OS=Rattus norvegicus OX=10116 GN=B2m PE=1 SV=1 | 13.7 | 19 | 10 | 3 | 10891864.36 | 8108647.695 | 6423150.578 |
| P36953 | Afamin OS=Rattus norvegicus OX=10116 GN=Afm PE=3 SV=1 | 69.3 | 57 | 377 | 34 | 677039444.3 | 765428373.3 | 604939588.9 |
| Q5M872 | Dipeptidase 2 OS=Rattus norvegicus OX=10116 GN=Dpep2 PE=2 SV=1 | 53.3 | 16 | 11 | 5 | 644782.0161 | 269259.1953 | 899497.75 |
| P29266 | 3-hydroxyisobutyrate dehydrogenase. mitochondrial OS=Rattus norvegicus OX=10116 GN=Hibadh PE=1 SV=3 | 35.3 | 10 | 2 | 2 |  | 352485.3668 | 507311.625 |
| P14046 | Alpha-1-inhibitor 3 OS=Rattus norvegicus OX=10116 GN=A1i3 PE=1 SV=1 | 163.7 | 62 | 10266 | 25 | 2615871044 | 3615688937 | 2725890083 |
| P55280 | Cadherin-6 OS=Rattus norvegicus OX=10116 GN=Cdh6 PE=1 SV=1 | 88.3 | 7 | 6 | 4 | 648969.312 | 540286.4045 | 2006358.133 |
| P06866 | Haptoglobin OS=Rattus norvegicus OX=10116 GN=Hp PE=1 SV=3 | 38.5 | 55 | 378 | 19 | 1005716907 | 1275197352 | 994118058.8 |
| B0BNN3 | Carbonic anhydrase 1 OS=Rattus norvegicus OX=10116 GN=Ca1 PE=1 SV=1 | 28.3 | 36 | 20 | 6 | 1324917.506 | 187173.1939 | 63705.30664 |
| Q6MG61 | Chloride intracellular channel protein 1 OS=Rattus norvegicus OX=10116 GN=Clic1 PE=1 SV=1 | 27 | 47 | 36 | 7 | 967873.7332 | 1006312.747 | 16413194.9 |
| Q9EPH1 | Alpha-1B-glycoprotein OS=Rattus norvegicus OX=10116 GN=A1bg PE=2 SV=2 | 56.4 | 57 | 892 | 23 | 2292084993 | 2102236634 | 1628865318 |
| Q63416 | Inter-alpha-trypsin inhibitor heavy chain H3 OS=Rattus norvegicus OX=10116 GN=Itih3 PE=2 SV=1 | 99 | 42 | 514 | 27 | 269276137.7 | 368937893.9 | 307730830.6 |
| P17475 | Alpha-1-antiproteinase OS=Rattus norvegicus OX=10116 GN=Serpina1 PE=1 SV=2 | 46.1 | 53 | 448 | 18 | 1642081029 | 1744928303 | 1274624635 |
| P20766 | Ig lambda-1 chain C region OS=Rattus norvegicus OX=10116 PE=4 SV=1 | 11.6 | 48 | 13 | 3 | 2223907.796 | 2915915.776 | 1806712.359 |
| P01015 | Angiotensinogen OS=Rattus norvegicus OX=10116 GN=Agt PE=1 SV=1 | 51.9 | 40 | 72 | 13 | 93667612.94 | 106086049 | 71792001.78 |
| Q6P6T1 | Complement C1s subcomponent OS=Rattus norvegicus OX=10116 GN=C1s PE=2 SV=2 | 77 | 31 | 103 | 17 | 7974732.302 | 39959319.31 | 26961834.64 |
| Q00495 | Macrophage colony-stimulating factor 1 receptor OS=Rattus norvegicus OX=10116 GN=Csf1r PE=1 SV=1 | 109.2 | 1 | 3 | 1 | 804957.8656 | 763683.1238 | 634900.875 |
| Q6P734 | Plasma protease C1 inhibitor OS=Rattus norvegicus OX=10116 GN=Serping1 PE=2 SV=1 | 55.6 | 41 | 306 | 19 | 194634022.4 | 292648780.1 | 244873310.2 |
| P20761 | Ig gamma-2B chain C region OS=Rattus norvegicus OX=10116 GN=Igh-1a PE=1 SV=1 | 36.5 | 37 | 645 | 9 | 1510405089 | 1264455772 | 1045110869 |
| P13635 | Ceruloplasmin OS=Rattus norvegicus OX=10116 GN=Cp PE=1 SV=3 | 120.8 | 52 | 849 | 41 | 1303462629 | 1162408091 | 929471857.2 |
| P62630 | Elongation factor 1-alpha 1 OS=Rattus norvegicus OX=10116 GN=Eef1a1 PE=2 SV=1 | 50.1 | 14 | 9 | 3 | 6163.600752 | 109012.9382 | 119015.457 |
| Q6IRK9 | Carboxypeptidase Q OS=Rattus norvegicus OX=10116 GN=Cpq PE=1 SV=1 | 52 | 27 | 51 | 9 | 30271174.63 | 23418996.69 | 21221597.5 |
| P02767 | Transthyretin OS=Rattus norvegicus OX=10116 GN=Ttr PE=1 SV=1 | 15.7 | 56 | 84 | 6 | 157284790.7 | 148264769.7 | 139660962.5 |
| P01805 | Ig heavy chain V region IR2 OS=Rattus norvegicus OX=10116 PE=4 SV=1 | 16 | 25 | 29 | 3 | 21779209.95 | 7181951.187 | 7244587.66 |
| Q03626 | Murinoglobulin-1 OS=Rattus norvegicus OX=10116 GN=Mug1 PE=2 SV=1 | 165.2 | 67 | 10281 | 24 | 2350135555 | 3023542221 | 2492381417 |
| P62716 | Serine/threonine-protein phosphatase 2A catalytic subunit beta isoform OS=Rattus norvegicus OX=10116 GN=Ppp2cb PE=2 SV=1 | 35.6 | 8 | 7 | 2 | 140138.0528 | 29403.43406 | 25635.94531 |
| P20762 | Ig gamma-2C chain C region OS=Rattus norvegicus OX=10116 PE=2 SV=1 | 36.5 | 47 | 176 | 11 | 241650215.4 | 272007028.1 | 222859910.8 |
| P32821 | Trypsin V-A OS=Rattus norvegicus OX=10116 PE=2 SV=1 | 26.9 | 8 | 8 | 1 | 4586398.263 | 3821873.534 | 3415759.688 |
| P05544 | Serine protease inhibitor A3L OS=Rattus norvegicus OX=10116 GN=Serpina3l PE=1 SV=3 | 46.2 | 65 | 1179 | 21 | 1175397000 | 2241464218 | 1533553522 |
| P01026 | Complement C3 OS=Rattus norvegicus OX=10116 GN=C3 PE=1 SV=3 | 186.3 | 69 | 4640 | 105 | 7332934562 | 7273235092 | 7172075413 |
| P12346 | Serotransferrin OS=Rattus norvegicus OX=10116 GN=Tf PE=1 SV=3 | 76.3 | 73 | 1149 | 53 | 7183492500 | 7165592541 | 6438967764 |
| P23680 | Serum amyloid P-component OS=Rattus norvegicus OX=10116 GN=Apcs PE=2 SV=2 | 26.2 | 62 | 99 | 9 | 99014628.72 | 98290401.6 | 85119681.62 |
| P02764 | Alpha-1-acid glycoprotein OS=Rattus norvegicus OX=10116 GN=Orm1 PE=2 SV=1 | 23.6 | 20 | 43 | 5 | 88773446.15 | 74818609.37 | 82366128.37 |
| P02466 | Collagen alpha-2(I) chain OS=Rattus norvegicus OX=10116 GN=Col1a2 PE=1 SV=3 | 129.5 | 2 | 7 | 2 |  | 1434581.381 | 631198.8125 |
| P20059 | Hemopexin OS=Rattus norvegicus OX=10116 GN=Hpx PE=1 SV=3 | 51.3 | 65 | 1401 | 28 | 3959100832 | 5595806417 | 5173799362 |
| P32038 | Complement factor D OS=Rattus norvegicus OX=10116 GN=Cfd PE=1 SV=2 | 28.4 | 40 | 35 | 5 | 21338628.16 | 15330110.84 | 12328228.81 |
| P13596 | Neural cell adhesion molecule 1 OS=Rattus norvegicus OX=10116 GN=Ncam1 PE=1 SV=1 | 94.6 | 10 | 11 | 5 | 830743.224 | 1184986.479 | 808833.3945 |
| Q62740 | Secreted phosphoprotein 24 OS=Rattus norvegicus OX=10116 GN=Spp2 PE=1 SV=2 | 23.2 | 11 | 4 | 2 | 69294.0248 | 138929.6592 | 185908.9961 |
| Q99J86 | Attractin OS=Rattus norvegicus OX=10116 GN=Atrn PE=2 SV=1 | 158.6 | 19 | 69 | 19 | 11587520.52 | 15477530.19 | 11520314.75 |
| Q01177 | Plasminogen OS=Rattus norvegicus OX=10116 GN=Plg PE=2 SV=2 | 90.5 | 58 | 247 | 33 | 310761308.1 | 364849971.9 | 291468457.6 |
| P02770 | Serum albumin OS=Rattus norvegicus OX=10116 GN=Alb PE=1 SV=2 | 68.7 | 84 | 13235 | 74 | 46515496542 | 53247665301 | 39238006004 |
| P29534 | Vascular cell adhesion protein 1 OS=Rattus norvegicus OX=10116 GN=Vcam1 PE=1 SV=1 | 81.2 | 13 | 16 | 6 | 1033779.47 | 1344366.895 | 1221146.461 |
| Q99M63 | WD40 repeat-containing protein SMU1 OS=Rattus norvegicus OX=10116 GN=Smu1 PE=2 SV=1 | 57.5 | 3 | 8 | 1 | 1581173.117 | 1940532.572 | 1920799.828 |
| P08934 | Kininogen-1 OS=Rattus norvegicus OX=10116 GN=Kng1 PE=2 SV=1 | 70.9 | 40 | 144 | 15 | 77917319.64 | 66868468.31 | 87515788.72 |
| D3ZZ80 | Obscurin-like protein 1 OS=Rattus norvegicus OX=10116 GN=Obsl1 PE=2 SV=3 | 197.7 | 1 | 5 | 1 | 80337.733 | 482157.3341 | 483560.5469 |
| P41498 | Low molecular weight phosphotyrosine protein phosphatase OS=Rattus norvegicus OX=10116 GN=Acp1 PE=1 SV=3 | 18.1 | 17 | 11 | 2 | 1201169.942 | 779068.5222 | 846766.1445 |
| P35859 | Insulin-like growth factor-binding protein complex acid labile subunit OS=Rattus norvegicus OX=10116 GN=Igfals PE=1 SV=1 | 66.8 | 35 | 150 | 14 | 110869996.7 | 114544139.6 | 73576790.71 |
| Q6IMF3 | Keratin. type II cytoskeletal 1 OS=Rattus norvegicus OX=10116 GN=Krt1 PE=2 SV=1 | 64.8 | 7 | 88 | 3 | 14263435.9 | 16166065.8 | 16289380.72 |
| P08661 | Mannose-binding protein C OS=Rattus norvegicus OX=10116 GN=Mbl2 PE=1 SV=2 | 26 | 15 | 24 | 3 | 2122697.038 | 2853853.069 | 3237913.828 |
| P20767 | Ig lambda-2 chain C region OS=Rattus norvegicus OX=10116 PE=4 SV=1 | 11.3 | 87 | 202 | 7 | 228641852.3 | 160050463 | 159076685.2 |
| Q63678 | Zinc-alpha-2-glycoprotein OS=Rattus norvegicus OX=10116 GN=Azgp1 PE=2 SV=1 | 34 | 42 | 33 | 10 | 30788659.93 | 28766385.51 | 25743586.2 |
| Q6IG04 | Keratin. type II cytoskeletal 72 OS=Rattus norvegicus OX=10116 GN=Krt72 PE=3 SV=2 | 56.8 | 4 | 28 | 1 | 190729.7213 | 453232.0256 | 479030.8242 |
| Q63621 | Interleukin-1 receptor accessory protein OS=Rattus norvegicus OX=10116 GN=Il1rap PE=2 SV=1 | 65.6 | 14 | 35 | 7 | 18484100.88 | 18073353.63 | 18680155.28 |
| Q4FZU2 | Keratin. type II cytoskeletal 6A OS=Rattus norvegicus OX=10116 GN=Krt6a PE=1 SV=1 | 59.2 | 8 | 65 | 3 | 8849549.975 | 10519060.08 | 10555837.06 |
| P52296 | Importin subunit beta-1 OS=Rattus norvegicus OX=10116 GN=Kpnb1 PE=1 SV=1 | 97.1 | 10 | 17 | 6 | 1201812.421 | 8909.629738 | 582743.084 |
| P0DP29 | Calmodulin-1 OS=Rattus norvegicus OX=10116 GN=Calm1 PE=1 SV=1 | 16.8 | 11 | 1 | 1 |  | 39804.98326 | 42456.42578 |
| P27139 | Carbonic anhydrase 2 OS=Rattus norvegicus OX=10116 GN=Ca2 PE=1 SV=2 | 29.1 | 65 | 188 | 12 | 322332849.2 | 242906510.6 | 272857574.2 |
| O70535 | Leukemia inhibitory factor receptor OS=Rattus norvegicus OX=10116 GN=Lifr PE=2 SV=1 | 122.3 | 25 | 156 | 19 | 56294100.69 | 75600468.49 | 72353195.71 |
| P14272 | Plasma kallikrein OS=Rattus norvegicus OX=10116 GN=Klkb1 PE=1 SV=1 | 71.2 | 38 | 88 | 15 | 44529700.07 | 47329681.56 | 45192721.32 |
| Q6IFW6 | Keratin. type I cytoskeletal 10 OS=Rattus norvegicus OX=10116 GN=Krt10 PE=3 SV=1 | 56.5 | 14 | 103 | 6 | 8163928.13 | 12098917.38 | 14512022.3 |
| P05545 | Serine protease inhibitor A3K OS=Rattus norvegicus OX=10116 GN=Serpina3k PE=1 SV=3 | 46.5 | 61 | 1022 | 19 | 560602715.5 | 1414108935 | 1015407768 |
| P00762 | Anionic trypsin-1 OS=Rattus norvegicus OX=10116 GN=Prss1 PE=1 SV=1 | 25.9 | 8 | 262 | 1 | 785663045.6 | 737808514.6 | 813515170 |
| Q6IE52 | Murinoglobulin-2 OS=Rattus norvegicus OX=10116 GN=Mug2 PE=1 SV=1 | 161.5 | 52 | 6937 | 11 | 23188107.32 | 35839639.38 | 40032348.77 |
| P08932 | T-kininogen 2 OS=Rattus norvegicus OX=10116 PE=1 SV=2 | 47.7 | 60 | 494 | 9 | 244658059.3 | 284585674.5 | 271787719 |
| Q62975 | Protein Z-dependent protease inhibitor OS=Rattus norvegicus OX=10116 GN=Serpina10 PE=2 SV=2 | 50.2 | 38 | 130 | 15 | 38882473.58 | 45615177.05 | 50961130.55 |
| P01836 | Ig kappa chain C region. A allele OS=Rattus norvegicus OX=10116 PE=1 SV=1 | 11.7 | 92 | 1046 | 10 | 1366320089 | 783375574.2 | 699671661.5 |
| P55314 | Complement component C8 beta chain OS=Rattus norvegicus OX=10116 GN=C8b PE=2 SV=2 | 66.6 | 45 | 147 | 22 | 40909856.31 | 40334610.58 | 51980865.8 |
| Q9EQV8 | Carboxypeptidase N catalytic chain OS=Rattus norvegicus OX=10116 GN=Cpn1 PE=2 SV=1 | 51.9 | 28 | 76 | 7 | 18323035 | 21724123.22 | 26770252.99 |
| P10860 | Glutamate dehydrogenase 1. mitochondrial OS=Rattus norvegicus OX=10116 GN=Glud1 PE=1 SV=2 | 61.4 | 8 | 6 | 4 | 670290.4855 | 462152.5679 | 793522.1172 |
| P10959 | Carboxylesterase 1C OS=Rattus norvegicus OX=10116 GN=Ces1c PE=1 SV=3 | 60.1 | 43 | 166 | 19 | 673666299.1 | 504478614.8 | 609650885.6 |
| P20760 | Ig gamma-2A chain C region OS=Rattus norvegicus OX=10116 GN=Igg-2a PE=1 SV=1 | 35.2 | 61 | 1054 | 15 | 3680052023 | 1723862294 | 2012849685 |
| Q9JJS8 | Mannan-binding lectin serine protease 2 OS=Rattus norvegicus OX=10116 GN=Masp2 PE=1 SV=2 | 75.6 | 18 | 33 | 8 | 1082389.579 | 2388965.691 | 2170887.758 |
| P16086 | Spectrin alpha chain. non-erythrocytic 1 OS=Rattus norvegicus OX=10116 GN=Sptan1 PE=1 SV=2 | 284.5 | 10 | 27 | 17 | 228929.3371 | 101681.6587 | 1428160.486 |
| Q62930 | Complement component C9 OS=Rattus norvegicus OX=10116 GN=C9 PE=1 SV=1 | 62.2 | 51 | 96 | 20 | 145073686.6 | 128875301.6 | 173213472.9 |
| Q8CHN8 | Mannan-binding lectin serine protease 1 OS=Rattus norvegicus OX=10116 GN=Masp1 PE=1 SV=2 | 80 | 21 | 46 | 11 | 2082480.553 | 3165910.654 | 3927939.621 |
| Q9QX79 | Fetuin-B OS=Rattus norvegicus OX=10116 GN=Fetub PE=2 SV=2 | 41.5 | 41 | 53 | 9 | 4466306.96 | 3989323.583 | 4522660.766 |
| P06760 | Beta-glucuronidase OS=Rattus norvegicus OX=10116 GN=Gusb PE=2 SV=1 | 74.7 | 2 | 2 | 1 | 382386.4678 | 141317.7626 | 173842.6563 |
| Q68FS2 | COP9 signalosome complex subunit 4 OS=Rattus norvegicus OX=10116 GN=Cops4 PE=1 SV=1 | 46.3 | 28 | 14 | 8 | 372065.672 | 304750.0332 | 376831.8721 |
| P06238 | Alpha-2-macroglobulin OS=Rattus norvegicus OX=10116 GN=A2m PE=2 SV=2 | 163.7 | 25 | 249 | 27 | 47435754.38 | 52778096.02 | 13801928.43 |
| P01048 | T-kininogen 1 OS=Rattus norvegicus OX=10116 GN=Map1 PE=1 SV=2 | 47.7 | 52 | 667 | 10 | 918178842.1 | 984121918.1 | 912666285.7 |
| P26644 | Beta-2-glycoprotein 1 OS=Rattus norvegicus OX=10116 GN=Apoh PE=2 SV=2 | 33.2 | 36 | 49 | 9 | 99501490.02 | 89672978.36 | 106024101.7 |
| O54735 | cGMP-specific 3'.5'-cyclic phosphodiesterase OS=Rattus norvegicus OX=10116 GN=Pde5a PE=2 SV=1 | 94.5 | 11 | 15 | 6 | 820418.9853 | 1983994.471 | 625457.1816 |
| P47853 | Biglycan OS=Rattus norvegicus OX=10116 GN=Bgn PE=1 SV=1 | 41.7 | 17 | 9 | 5 | 97718.33155 | 87654.82838 | 823404.4453 |
| Q9WUW3 | Complement factor I OS=Rattus norvegicus OX=10116 GN=Cfi PE=2 SV=1 | 67.3 | 52 | 184 | 20 | 63895289.62 | 109166207.3 | 132255598.2 |
| P04937 | Fibronectin OS=Rattus norvegicus OX=10116 GN=Fn1 PE=1 SV=2 | 272.3 | 35 | 538 | 55 | 25697190.02 | 113930759.6 | 153515730.7 |
| O70513 | Galectin-3-binding protein OS=Rattus norvegicus OX=10116 GN=Lgals3bp PE=1 SV=2 | 63.7 | 20 | 24 | 9 | 1718910.685 | 1141926.194 | 1270176.459 |
| P31211 | Corticosteroid-binding globulin OS=Rattus norvegicus OX=10116 GN=Serpina6 PE=1 SV=2 | 44.6 | 42 | 232 | 14 | 395818779.1 | 366019789.2 | 383734112.3 |
| P24090 | Alpha-2-HS-glycoprotein OS=Rattus norvegicus OX=10116 GN=Ahsg PE=1 SV=2 | 38 | 39 | 175 | 8 | 119415436 | 99046058.84 | 193986339.8 |
| Q64268 | Heparin cofactor 2 OS=Rattus norvegicus OX=10116 GN=Serpind1 PE=1 SV=1 | 54.5 | 34 | 128 | 12 | 56117681.57 | 70707877.29 | 109585463.2 |
| P01883 | Ig delta chain C region (Fragment) OS=Rattus norvegicus OX=10116 PE=2 SV=1 | 22.4 | 32 | 19 | 4 | 641634.946 | 1231197.332 | 1786066.578 |
| P28077 | Proteasome subunit beta type-9 OS=Rattus norvegicus OX=10116 GN=Psmb9 PE=1 SV=2 | 23.3 | 10 | 5 | 2 | 533669.2856 | 174760.9168 | 233321.957 |
| Q6IFV3 | Keratin. type I cytoskeletal 15 OS=Rattus norvegicus OX=10116 GN=Krt15 PE=1 SV=1 | 48.8 | 9 | 11 | 4 | 2339062.756 | 992901.0462 | 2667587.469 |
| Q811M5 | Complement component C6 OS=Rattus norvegicus OX=10116 GN=C6 PE=2 SV=1 | 105 | 40 | 116 | 25 | 26757378.1 | 27712703.28 | 41901229.6 |
| P20759 | Ig gamma-1 chain C region OS=Rattus norvegicus OX=10116 PE=1 SV=1 | 35.9 | 47 | 424 | 7 | 270039440.8 | 132891747.4 | 216490324 |
| P31721 | Complement C1q subcomponent subunit B OS=Rattus norvegicus OX=10116 GN=C1qb PE=1 SV=2 | 26.6 | 21 | 25 | 4 | 1576482.971 | 522384.9572 | 2148720.867 |
| P01681 | Ig kappa chain V region S211 OS=Rattus norvegicus OX=10116 PE=1 SV=1 | 11.9 | 36 | 14 | 3 | 1928398.47 | 1273527.425 | 266240.4688 |
| P97532 | 3-mercaptopyruvate sulfurtransferase OS=Rattus norvegicus OX=10116 GN=Mpst PE=1 SV=3 | 32.9 | 28 | 16 | 5 | 1409620.291 | 195357.2617 | 1075074.688 |
| P97594 | Mast cell protease 8 OS=Rattus norvegicus OX=10116 GN=Mcpt8 PE=2 SV=1 | 27.5 | 7 | 2 | 2 |  | 26248.59258 | 92208.30859 |
| P19999 | Mannose-binding protein A OS=Rattus norvegicus OX=10116 GN=Mbl1 PE=1 SV=1 | 25.3 | 5 | 12 | 1 | 1189377.272 | 1210712.846 | 2462857.656 |
| Q63041 | Alpha-1-macroglobulin OS=Rattus norvegicus OX=10116 GN=A1m PE=1 SV=1 | 167 | 74 | 7255 | 88 | 4757899621 | 3943160040 | 6384497109 |
| P11517 | Hemoglobin subunit beta-2 OS=Rattus norvegicus OX=10116 PE=1 SV=2 | 16 | 85 | 828 | 2 | 89167625.52 | 36157571.95 | 60574735.4 |
| P13697 | NADP-dependent malic enzyme OS=Rattus norvegicus OX=10116 GN=Me1 PE=1 SV=2 | 64 | 31 | 17 | 11 | 184527.2189 | 392768.5044 | 1704064.816 |
| Q5FVF9 | Biotinidase OS=Rattus norvegicus OX=10116 GN=Btd PE=2 SV=1 | 58 | 39 | 37 | 12 | 4600777.879 | 3953180.685 | 6294826.141 |
| Q6QMY6 | Tsukushin OS=Rattus norvegicus OX=10116 GN=Tsku PE=2 SV=1 | 38.1 | 4 | 4 | 1 | 23884.64369 | 20418.33059 | 35097.19922 |
| P17046 | Lysosome-associated membrane glycoprotein 2 OS=Rattus norvegicus OX=10116 GN=Lamp2 PE=1 SV=3 | 45.1 | 2 | 2 | 1 | 164297.6191 | 457748.9077 | 794096.4375 |
| P22985 | Xanthine dehydrogenase/oxidase OS=Rattus norvegicus OX=10116 GN=Xdh PE=1 SV=3 | 146.1 | 28 | 86 | 22 | 7848488.614 | 5567131.232 | 9365684.783 |
| P18292 | Prothrombin OS=Rattus norvegicus OX=10116 GN=F2 PE=1 SV=1 | 70.4 | 47 | 364 | 25 | 154134717.6 | 284967591.5 | 482797772.2 |
| Q68FP1 | Gelsolin OS=Rattus norvegicus OX=10116 GN=Gsn PE=1 SV=1 | 86 | 49 | 480 | 21 | 187980065.3 | 174030447.6 | 321148949.6 |
| P15684 | Aminopeptidase N OS=Rattus norvegicus OX=10116 GN=Anpep PE=1 SV=2 | 109.4 | 11 | 9 | 6 | 431667.875 | 430798.6064 | 509842.25 |
| Q9ESW0 | DNA damage-binding protein 1 OS=Rattus norvegicus OX=10116 GN=Ddb1 PE=1 SV=1 | 126.8 | 5 | 9 | 5 | 505362.8002 | 461808.2148 | 1578312.383 |
| Q8CJH6 | Neuronal PAS domain-containing protein 4 OS=Rattus norvegicus OX=10116 GN=Npas4 PE=1 SV=1 | 87.3 | 1 | 12 | 1 | 90684041.54 | 72993116.18 | 138327597.2 |
| P02651 | Apolipoprotein A-IV OS=Rattus norvegicus OX=10116 GN=Apoa4 PE=1 SV=2 | 44.4 | 65 | 150 | 19 | 772817500.1 | 554212598.9 | 250642753.9 |
| P83868 | Prostaglandin E synthase 3 OS=Rattus norvegicus OX=10116 GN=Ptges3 PE=1 SV=2 | 18.7 | 26 | 4 | 3 |  | 2460487.481 | 6884536.539 |
| P29598 | Urokinase-type plasminogen activator OS=Rattus norvegicus OX=10116 GN=Plau PE=1 SV=1 | 47.9 | 2 | 1 | 1 |  | 27746.45641 | 56985.31641 |
| G3V7W1 | Programmed cell death protein 6 OS=Rattus norvegicus OX=10116 GN=Pdcd6 PE=2 SV=1 | 21.9 | 6 | 13 | 1 | 4004192.259 | 12908488.01 | 26769933 |
| O08770 | Platelet glycoprotein V OS=Rattus norvegicus OX=10116 GN=Gp5 PE=3 SV=1 | 63.3 | 22 | 34 | 8 | 5462046.033 | 2498502.319 | 5159904.195 |
| P04276 | Vitamin D-binding protein OS=Rattus norvegicus OX=10116 GN=Gc PE=1 SV=3 | 53.5 | 77 | 821 | 31 | 934277848.2 | 905741951.2 | 1224006511 |
| P05371 | Clusterin OS=Rattus norvegicus OX=10116 GN=Clu PE=1 SV=2 | 51.3 | 28 | 132 | 11 | 11624854.78 | 11628505.49 | 21920618.66 |
| P31044 | Phosphatidylethanolamine-binding protein 1 OS=Rattus norvegicus OX=10116 GN=Pebp1 PE=1 SV=3 | 20.8 | 61 | 17 | 6 | 9293485.18 | 9373727.442 | 7561572.516 |
| O35217 | Multiple inositol polyphosphate phosphatase 1 OS=Rattus norvegicus OX=10116 GN=Minpp1 PE=1 SV=3 | 54.6 | 8 | 6 | 3 |  | 272085.2194 | 1498722.25 |
| Q920P6 | Adenosine deaminase OS=Rattus norvegicus OX=10116 GN=Ada PE=1 SV=3 | 39.9 | 7 | 2 | 2 | 309567.7249 | 330046.657 | 751113.6875 |
| Q6IFV4 | Keratin. type I cytoskeletal 13 OS=Rattus norvegicus OX=10116 GN=Krt13 PE=3 SV=1 | 47.7 | 9 | 35 | 4 | 207833.9766 | 645102.3196 | 2092882.953 |
| P08649 | Complement C4 OS=Rattus norvegicus OX=10116 GN=C4 PE=1 SV=3 | 192 | 56 | 1080 | 65 | 637223417.6 | 451159419 | 996478963.4 |
| P59996 | Proprotein convertase subtilisin/kexin type 9 OS=Rattus norvegicus OX=10116 GN=Pcsk9 PE=1 SV=1 | 74.7 | 18 | 20 | 8 | 45476.90433 | 438593.2635 | 224069.6807 |
| B0BNE5 | S-formylglutathione hydrolase OS=Rattus norvegicus OX=10116 GN=Esd PE=2 SV=1 | 31.3 | 43 | 30 | 7 | 8911846.648 | 4144078.164 | 4821271.824 |
| Q6P6Q2 | Keratin. type II cytoskeletal 5 OS=Rattus norvegicus OX=10116 GN=Krt5 PE=1 SV=1 | 61.8 | 11 | 55 | 4 | 261550.6049 | 660903.2234 | 1715628.96 |
| Q9JHL4 | Drebrin-like protein OS=Rattus norvegicus OX=10116 GN=Dbnl PE=1 SV=1 | 48.6 | 3 | 1 | 1 |  | 51130.59499 | 133709.8281 |
| P02091 | Hemoglobin subunit beta-1 OS=Rattus norvegicus OX=10116 GN=Hbb PE=1 SV=3 | 16 | 97 | 1043 | 5 | 1856600679 | 447654830.9 | 1210687781 |
| Q9QX69 | Glutathione S-transferase LANCL1 OS=Rattus norvegicus OX=10116 GN=Lancl1 PE=1 SV=2 | 45.2 | 18 | 21 | 5 | 960623.9333 | 559970.0105 | 1856989.102 |
| P26051 | CD44 antigen OS=Rattus norvegicus OX=10116 GN=Cd44 PE=1 SV=2 | 55.9 | 4 | 9 | 2 | 846520.9436 | 935255.0214 | 2513633.313 |
| Q4FZT6 | Histone H2A type 3 OS=Rattus norvegicus OX=10116 PE=2 SV=3 | 14.1 | 22 | 17 | 2 | 1886847.318 | 2978955.224 | 32284009.75 |
| P85973 | Purine nucleoside phosphorylase OS=Rattus norvegicus OX=10116 GN=Pnp PE=1 SV=1 | 32.3 | 45 | 32 | 10 | 29590941.3 | 6775651.263 | 18661383.53 |
| Q66HD0 | Endoplasmin OS=Rattus norvegicus OX=10116 GN=Hsp90b1 PE=1 SV=2 | 92.7 | 6 | 10 | 3 | 253748.0083 | 108279.4026 | 1459352.266 |
| P11442 | Clathrin heavy chain 1 OS=Rattus norvegicus OX=10116 GN=Cltc PE=1 SV=3 | 191.5 | 14 | 28 | 18 | 1352469.142 | 20803.46693 | 735009.3125 |
| P00773 | Chymotrypsin-like elastase family member 1 OS=Rattus norvegicus OX=10116 GN=Cela1 PE=1 SV=1 | 29 | 8 | 9 | 1 | 138557.8243 | 94154.50701 | 260582.4688 |
| P01946 | Hemoglobin subunit alpha-1/2 OS=Rattus norvegicus OX=10116 GN=Hba1 PE=1 SV=3 | 15.3 | 89 | 1197 | 12 | 5580163635 | 1757721703 | 4372933525 |
| P35704 | Peroxiredoxin-2 OS=Rattus norvegicus OX=10116 GN=Prdx2 PE=1 SV=3 | 21.8 | 43 | 90 | 7 | 88405984.85 | 9723336.873 | 27050115.1 |
| Q9ER34 | Aconitate hydratase. mitochondrial OS=Rattus norvegicus OX=10116 GN=Aco2 PE=1 SV=2 | 85.4 | 5 | 2 | 2 |  | 1924545.397 | 18542406.22 |
| Q3MIF4 | Xylulose kinase OS=Rattus norvegicus OX=10116 GN=Xylb PE=2 SV=1 | 57.9 | 12 | 9 | 3 | 303180.5619 | 381720.5362 | 966403.2227 |
| P04642 | L-lactate dehydrogenase A chain OS=Rattus norvegicus OX=10116 GN=Ldha PE=1 SV=1 | 36.4 | 19 | 12 | 4 | 108375.0449 | 81577.50308 | 655435.6816 |
| P02454 | Collagen alpha-1(I) chain OS=Rattus norvegicus OX=10116 GN=Col1a1 PE=1 SV=5 | 137.9 | 7 | 16 | 7 | 450246.3667 | 4453847.748 | 81689.71875 |
| Q9EPF2 | Cell surface glycoprotein MUC18 OS=Rattus norvegicus OX=10116 GN=Mcam PE=1 SV=2 | 71.3 | 25 | 22 | 10 | 1407379.115 | 1202978.187 | 10589370.91 |
| Q64537 | Calpain small subunit 1 OS=Rattus norvegicus OX=10116 GN=Capns1 PE=1 SV=3 | 28.6 | 13 | 4 | 2 | 1981695.537 | 381653.4156 | 1197580.891 |
| P08592 | Amyloid-beta A4 protein OS=Rattus norvegicus OX=10116 GN=App PE=1 SV=2 | 86.6 | 10 | 19 | 5 | 1339443.464 | 182001.8241 | 1719533.938 |
| P08025 | Insulin-like growth factor I OS=Rattus norvegicus OX=10116 GN=Igf1 PE=1 SV=3 | 17.1 | 10 | 1 | 1 | 47020.06979 | 71101.35438 | 226861.2344 |
| P53813 | Vitamin K-dependent protein S OS=Rattus norvegicus OX=10116 GN=Pros1 PE=2 SV=1 | 74.6 | 11 | 24 | 7 | 1715250.597 | 752916.9919 | 5195726.102 |
| P60711 | Actin. cytoplasmic 1 OS=Rattus norvegicus OX=10116 GN=Actb PE=1 SV=1 | 41.7 | 68 | 663 | 10 | 486055210.2 | 288141185.8 | 826891685.6 |
| P68511 | 14-3-3 protein eta OS=Rattus norvegicus OX=10116 GN=Ywhah PE=1 SV=2 | 28.2 | 32 | 43 | 4 | 2586052.334 | 1248351.689 | 4502208.859 |
| P31722 | Complement C1q subcomponent subunit C OS=Rattus norvegicus OX=10116 GN=C1qc PE=1 SV=2 | 25.7 | 11 | 3 | 2 | 288650.5272 | 38447.65017 | 407616.5234 |
| Q4V7D6 | NIF3-like protein 1 OS=Rattus norvegicus OX=10116 GN=Nif3l1 PE=1 SV=1 | 41.5 | 3 | 2 | 1 | 299311.2472 | 177432.1281 | 611237.1875 |
| P97536 | Cullin-associated NEDD8-dissociated protein 1 OS=Rattus norvegicus OX=10116 GN=Cand1 PE=1 SV=1 | 136.3 | 12 | 22 | 11 | 204966.3425 | 759607.3131 | 3093264.512 |
| P28037 | Cytosolic 10-formyltetrahydrofolate dehydrogenase OS=Rattus norvegicus OX=10116 GN=Aldh1l1 PE=1 SV=3 | 98.8 | 9 | 7 | 6 |  | 10120.31827 | 339476.4688 |
| Q5XI73 | Rho GDP-dissociation inhibitor 1 OS=Rattus norvegicus OX=10116 GN=Arhgdia PE=1 SV=1 | 23.4 | 51 | 18 | 8 | 12953506.75 | 1380565.512 | 20526121.97 |
| P70645 | Bleomycin hydrolase OS=Rattus norvegicus OX=10116 GN=Blmh PE=1 SV=1 | 52.3 | 53 | 139 | 19 | 72782309.02 | 24184608.36 | 47207211.06 |
| Q08420 | Extracellular superoxide dismutase [Cu-Zn] OS=Rattus norvegicus OX=10116 GN=Sod3 PE=1 SV=2 | 26.6 | 34 | 22 | 5 | 6372139.448 | 5851978.625 | 9617704.68 |
| P30835 | ATP-dependent 6-phosphofructokinase. liver type OS=Rattus norvegicus OX=10116 GN=Pfkl PE=1 SV=3 | 85.3 | 18 | 26 | 9 | 1452738.513 | 414042.232 | 1620454.6 |
| Q9WTS8 | Ficolin-1 OS=Rattus norvegicus OX=10116 GN=Fcn1 PE=2 SV=2 | 36.6 | 12 | 16 | 4 | 577736.284 | 520621.7047 | 1821505.969 |
| P31720 | Complement C1q subcomponent subunit A OS=Rattus norvegicus OX=10116 GN=C1qa PE=1 SV=2 | 25.9 | 6 | 7 | 1 | 295967.0929 | 159749.4508 | 613600.9063 |
| P62250 | 40S ribosomal protein S16 OS=Rattus norvegicus OX=10116 GN=Rps16 PE=1 SV=2 | 16.4 | 15 | 4 | 2 | 119354.7454 | 16085.17704 | 61807.33594 |
| P23739 | Sucrase-isomaltase. intestinal OS=Rattus norvegicus OX=10116 GN=Si PE=1 SV=5 | 210.2 | 1 | 2 | 1 |  | 5420.939081 | 21118.13965 |
| Q63515 | C4b-binding protein beta chain OS=Rattus norvegicus OX=10116 GN=C4bpb PE=2 SV=1 | 28.6 | 12 | 4 | 3 | 18889.67175 | 75317.23048 | 703271.8125 |
| Q9JLJ3 | 4-trimethylaminobutyraldehyde dehydrogenase OS=Rattus norvegicus OX=10116 GN=Aldh9a1 PE=1 SV=1 | 53.6 | 17 | 11 | 6 | 732168.2159 | 487736.5433 | 1911506.504 |
| P50297 | Arylamine N-acetyltransferase 1 OS=Rattus norvegicus OX=10116 GN=Nat1 PE=2 SV=1 | 33.4 | 9 | 2 | 2 |  | 71198.17606 | 285349.75 |
| P06214 | Delta-aminolevulinic acid dehydratase OS=Rattus norvegicus OX=10116 GN=Alad PE=1 SV=1 | 36 | 64 | 85 | 13 | 18435380.71 | 4853343.028 | 23689428.11 |
| P14480 | Fibrinogen beta chain OS=Rattus norvegicus OX=10116 GN=Fgb PE=1 SV=4 | 54.2 | 67 | 442 | 27 | 3129253.318 | 110491157.7 | 486638587.2 |
| P62828 | GTP-binding nuclear protein Ran OS=Rattus norvegicus OX=10116 GN=Ran PE=1 SV=3 | 24.4 | 31 | 23 | 5 | 2295620.345 | 650628.6173 | 2745905.656 |
| P19132 | Ferritin heavy chain OS=Rattus norvegicus OX=10116 GN=Fth1 PE=1 SV=3 | 21.1 | 37 | 40 | 6 | 6692982.729 | 762521.9415 | 9249140.43 |
| Q63716 | Peroxiredoxin-1 OS=Rattus norvegicus OX=10116 GN=Prdx1 PE=1 SV=1 | 22.1 | 47 | 45 | 8 | 37309718.8 | 15386884.2 | 47460161.37 |
| P35559 | Insulin-degrading enzyme OS=Rattus norvegicus OX=10116 GN=Ide PE=1 SV=1 | 117.6 | 18 | 27 | 12 | 3714001.372 | 324035.6055 | 2711071.895 |
| Q05982 | Nucleoside diphosphate kinase A OS=Rattus norvegicus OX=10116 GN=Nme1 PE=1 SV=1 | 17.2 | 65 | 100 | 3 | 25250329.49 | 4610134.981 | 20601754.28 |
| P23562 | Band 3 anion transport protein OS=Rattus norvegicus OX=10116 GN=Slc4a1 PE=1 SV=3 | 103.1 | 8 | 18 | 5 | 1250238.973 | 734714.0043 | 3076558.254 |
| P06399 | Fibrinogen alpha chain OS=Rattus norvegicus OX=10116 GN=Fga PE=1 SV=3 | 86.6 | 30 | 261 | 21 | 13138784.32 | 67283987.65 | 338039326.9 |
| P18297 | Sepiapterin reductase OS=Rattus norvegicus OX=10116 GN=Spr PE=1 SV=1 | 28.1 | 46 | 48 | 8 | 2301415.268 | 738565.1318 | 3211728.16 |
| Q64230 | Meprin A subunit alpha OS=Rattus norvegicus OX=10116 GN=Mep1a PE=1 SV=2 | 85.1 | 8 | 8 | 5 | 63867.33786 | 40990.86069 | 548120.2031 |
| P04785 | Protein disulfide-isomerase OS=Rattus norvegicus OX=10116 GN=P4hb PE=1 SV=2 | 56.9 | 35 | 30 | 13 | 1485609.829 | 3569908.917 | 16017388.96 |
| P68136 | Actin. alpha skeletal muscle OS=Rattus norvegicus OX=10116 GN=Acta1 PE=1 SV=1 | 42 | 38 | 198 | 3 | 95193468.82 | 29595599.99 | 163661318 |
| P51886 | Lumican OS=Rattus norvegicus OX=10116 GN=Lum PE=1 SV=1 | 38.3 | 33 | 177 | 10 | 105850162.2 | 86372660.34 | 441680290.3 |
| Q8R2H5 | Phosphatidylinositol-glycan-specific phospholipase D OS=Rattus norvegicus OX=10116 GN=Gpld1 PE=2 SV=1 | 93.7 | 12 | 18 | 8 | 957114.8532 | 445443.9595 | 2632660.18 |
| O08619 | Coagulation factor XIII A chain OS=Rattus norvegicus OX=10116 GN=F13a1 PE=2 SV=3 | 82.6 | 11 | 18 | 7 | 173359.733 | 156130.5417 | 1382671.465 |
| Q6Q0N1 | Cytosolic non-specific dipeptidase OS=Rattus norvegicus OX=10116 GN=Cndp2 PE=1 SV=1 | 52.7 | 16 | 8 | 5 | 54304.28664 | 82382.32718 | 1813461.323 |
| B2GUZ5 | F-actin-capping protein subunit alpha-1 OS=Rattus norvegicus OX=10116 GN=Capza1 PE=1 SV=1 | 32.9 | 23 | 5 | 3 | 820038.9086 | 321876.2228 | 2476134.906 |
| Q6AY09 | Heterogeneous nuclear ribonucleoprotein H2 OS=Rattus norvegicus OX=10116 GN=Hnrnph2 PE=1 SV=1 | 49.3 | 6 | 11 | 2 | 83842.80186 | 8275.111337 | 320161.2422 |
| Q7TP52 | Carboxymethylenebutenolidase homolog OS=Rattus norvegicus OX=10116 GN=Cmbl PE=2 SV=1 | 27.9 | 29 | 44 | 8 | 601216.5968 | 693999.1378 | 11112712.47 |
| Q4KLZ6 | Triokinase/FMN cyclase OS=Rattus norvegicus OX=10116 GN=Tkfc PE=1 SV=1 | 59.4 | 32 | 37 | 12 | 3213997.572 | 968925 | 24847634.89 |
| P19112 | Fructose-1.6-bisphosphatase 1 OS=Rattus norvegicus OX=10116 GN=Fbp1 PE=1 SV=2 | 39.6 | 52 | 79 | 16 | 707506.3491 | 198250.6566 | 54142334.64 |
| P02680 | Fibrinogen gamma chain OS=Rattus norvegicus OX=10116 GN=Fgg PE=1 SV=3 | 50.6 | 45 | 358 | 17 | 5430348.491 | 131975072.9 | 603464056.1 |
| Q5U2Q3 | Ester hydrolase C11orf54 homolog OS=Rattus norvegicus OX=10116 PE=1 SV=1 | 35 | 30 | 25 | 7 | 59533.80804 | 838062.4621 | 16007022.62 |
| O55096 | Dipeptidyl peptidase 3 OS=Rattus norvegicus OX=10116 GN=Dpp3 PE=1 SV=2 | 83 | 19 | 14 | 7 | 1118016.41 | 255542.0451 | 6906132.438 |
| P25304 | Agrin OS=Rattus norvegicus OX=10116 GN=Agrn PE=1 SV=2 | 208.5 | 8 | 16 | 8 | 352127.3519 | 411375.1547 | 797641.2656 |
| P10719 | ATP synthase subunit beta. mitochondrial OS=Rattus norvegicus OX=10116 GN=Atp5f1b PE=1 SV=2 | 56.3 | 6 | 8 | 2 | 4212566.954 | 1926744.66 | 10555080.69 |
| P04762 | Catalase OS=Rattus norvegicus OX=10116 GN=Cat PE=1 SV=3 | 59.7 | 70 | 510 | 27 | 1122604756 | 122573826.1 | 683807832.3 |
| P62804 | Histone H4 OS=Rattus norvegicus OX=10116 GN=Hist1h4b PE=1 SV=2 | 11.4 | 43 | 32 | 6 | 1439055.973 | 2685890.015 | 10429008.93 |
| P05065 | Fructose-bisphosphate aldolase A OS=Rattus norvegicus OX=10116 GN=Aldoa PE=1 SV=2 | 39.3 | 70 | 161 | 15 | 117414397.2 | 21720408.47 | 120694934.1 |
| Q811X6 | Lambda-crystallin homolog OS=Rattus norvegicus OX=10116 GN=Cryl1 PE=1 SV=3 | 35.3 | 67 | 52 | 13 | 930884.7425 | 5538186.291 | 135583478 |
| P63182 | Cerebellin-1 OS=Rattus norvegicus OX=10116 GN=Cbln1 PE=1 SV=2 | 21.1 | 4 | 1 | 1 | 214749.4447 | 34589.71269 | 195140.7031 |
| Q63028 | Alpha-adducin OS=Rattus norvegicus OX=10116 GN=Add1 PE=1 SV=2 | 80.3 | 22 | 16 | 8 | 311426.4035 | 45724.24307 | 4751936.238 |
| P09330 | Ribose-phosphate pyrophosphokinase 2 OS=Rattus norvegicus OX=10116 GN=Prps2 PE=1 SV=3 | 34.8 | 16 | 9 | 4 | 543165.2437 | 17551.56879 | 148122.332 |
| O35828 | Coronin-7 OS=Rattus norvegicus OX=10116 GN=Coro7 PE=1 SV=2 | 100.7 | 6 | 2 | 2 |  | 14117.25416 | 83664.66406 |
| P55159 | Serum paraoxonase/arylesterase 1 OS=Rattus norvegicus OX=10116 GN=Pon1 PE=1 SV=3 | 39.3 | 37 | 77 | 8 | 14680151.4 | 4973419.196 | 24717494.23 |
| P13676 | Acylamino-acid-releasing enzyme OS=Rattus norvegicus OX=10116 GN=Apeh PE=1 SV=1 | 81.3 | 62 | 200 | 25 | 65208030.42 | 15626979.46 | 51786993.86 |
| P16296 | Coagulation factor IX OS=Rattus norvegicus OX=10116 GN=F9 PE=2 SV=2 | 51.8 | 49 | 131 | 15 | 134762218.8 | 31618329.41 | 167137599.3 |
| P84245 | Histone H3.3 OS=Rattus norvegicus OX=10116 GN=H3f3b PE=1 SV=2 | 15.3 | 5 | 6 | 1 | 206959.9733 | 529319.4121 | 3249001 |
| P12711 | Alcohol dehydrogenase class-3 OS=Rattus norvegicus OX=10116 GN=Adh5 PE=1 SV=2 | 39.6 | 16 | 5 | 3 | 277958.2871 | 214396.893 | 4055159.75 |
| Q6AYC4 | Macrophage-capping protein OS=Rattus norvegicus OX=10116 GN=Capg PE=1 SV=1 | 38.8 | 8 | 2 | 2 | 154796.6037 | 17251.79066 | 106862.2813 |
| Q05764 | Beta-adducin OS=Rattus norvegicus OX=10116 GN=Add2 PE=1 SV=4 | 80.5 | 6 | 8 | 3 | 436105.6007 | 159187.1096 | 1933549.5 |
| P63018 | Heat shock cognate 71 kDa protein OS=Rattus norvegicus OX=10116 GN=Hspa8 PE=1 SV=1 | 70.8 | 45 | 307 | 23 | 303980719.6 | 67680436.31 | 330785303.1 |
| D3ZXK7 | E3 ubiquitin-protein ligase RNF123 OS=Rattus norvegicus OX=10116 GN=Rnf123 PE=1 SV=1 | 149 | 2 | 1 | 1 | 60889.98819 | 23866.44181 | 152975.5156 |
| O35567 | Bifunctional purine biosynthesis protein PURH OS=Rattus norvegicus OX=10116 GN=Atic PE=1 SV=2 | 64.2 | 62 | 106 | 25 | 98889785.38 | 10791218.25 | 70650529.03 |
| P85972 | Vinculin OS=Rattus norvegicus OX=10116 GN=Vcl PE=1 SV=1 | 116.5 | 24 | 50 | 17 | 6090655.81 | 1076246.586 | 29021239.85 |
| Q62894 | Extracellular matrix protein 1 OS=Rattus norvegicus OX=10116 GN=Ecm1 PE=1 SV=2 | 63.2 | 33 | 47 | 15 | 5004522.581 | 2614582.575 | 34423880.57 |
| P04764 | Alpha-enolase OS=Rattus norvegicus OX=10116 GN=Eno1 PE=1 SV=4 | 47.1 | 34 | 37 | 7 | 1667621.704 | 261993.0285 | 6449409.375 |
| Q9Z1P2 | Alpha-actinin-1 OS=Rattus norvegicus OX=10116 GN=Actn1 PE=1 SV=1 | 102.9 | 56 | 199 | 24 | 2486202.69 | 3173684.386 | 23616570.26 |
| Q9Z1Y3 | Cadherin-2 OS=Rattus norvegicus OX=10116 GN=Cdh2 PE=1 SV=1 | 99.6 | 11 | 6 | 4 | 43763.55042 | 61122.97135 | 644836.8047 |
| Q5RKI0 | WD repeat-containing protein 1 OS=Rattus norvegicus OX=10116 GN=Wdr1 PE=1 SV=3 | 66.1 | 17 | 14 | 6 | 1167304.387 | 169707.4757 | 8931736.484 |
| P85968 | 6-phosphogluconate dehydrogenase. decarboxylating OS=Rattus norvegicus OX=10116 GN=Pgd PE=1 SV=1 | 53.2 | 22 | 14 | 7 | 15959478.36 | 1159428.101 | 14271056.56 |
| Q6AYZ1 | Tubulin alpha-1C chain OS=Rattus norvegicus OX=10116 GN=Tuba1c PE=1 SV=1 | 49.9 | 40 | 92 | 5 | 2139523.406 | 513107.478 | 6623819.711 |
| Q63797 | Proteasome activator complex subunit 1 OS=Rattus norvegicus OX=10116 GN=Psme1 PE=2 SV=1 | 28.6 | 20 | 4 | 4 | 385433.9627 | 91041.28363 | 2794945.781 |
| A1A5Q7 | von Willebrand factor A domain-containing protein 3A OS=Rattus norvegicus OX=10116 GN=Vwa3a PE=2 SV=1 | 83.2 | 1 | 2 | 1 | 282366.4017 | 29946.6436 | 216105.4844 |
| Q9WTT6 | Guanine deaminase OS=Rattus norvegicus OX=10116 GN=Gda PE=1 SV=1 | 51 | 69 | 140 | 23 | 128128803.3 | 5143677.271 | 31083834.08 |
| Q9ESS6 | Basal cell adhesion molecule OS=Rattus norvegicus OX=10116 GN=Bcam PE=2 SV=1 | 67.5 | 26 | 28 | 9 | 967124.1623 | 453388.6809 | 8194355.867 |
| P32755 | 4-hydroxyphenylpyruvate dioxygenase OS=Rattus norvegicus OX=10116 GN=Hpd PE=1 SV=3 | 45.1 | 53 | 40 | 15 | 10032332.84 | 6227462.624 | 66031916.72 |
| Q6P7Q4 | Lactoylglutathione lyase OS=Rattus norvegicus OX=10116 GN=Glo1 PE=1 SV=3 | 20.8 | 58 | 20 | 7 | 25048264.33 | 3298477.771 | 27081653 |
| P28075 | Proteasome subunit beta type-5 OS=Rattus norvegicus OX=10116 GN=Psmb5 PE=1 SV=3 | 28.6 | 26 | 47 | 6 | 5747328.762 | 361851.9042 | 5246256.961 |
| Q63530 | Phosphotriesterase-related protein OS=Rattus norvegicus OX=10116 GN=Pter PE=2 SV=2 | 39.1 | 34 | 13 | 7 | 957050.05 | 88673.13624 | 4060232.453 |
| P04917 | Serglycin OS=Rattus norvegicus OX=10116 GN=Srgn PE=1 SV=1 | 18.6 | 16 | 9 | 2 | 184847.2297 | 86973.79084 | 858693.9844 |
| P54759 | Ephrin type-A receptor 7 OS=Rattus norvegicus OX=10116 GN=Epha7 PE=1 SV=1 | 111.9 | 4 | 3 | 3 |  | 18813.48326 | 851651.6563 |
| P04041 | Glutathione peroxidase 1 OS=Rattus norvegicus OX=10116 GN=Gpx1 PE=1 SV=4 | 22.3 | 63 | 167 | 12 | 446356876 | 62078041.52 | 516604034.2 |
| Q63514 | C4b-binding protein alpha chain OS=Rattus norvegicus OX=10116 GN=C4bpa PE=2 SV=1 | 62.2 | 24 | 74 | 11 | 2971830.32 | 2099655.783 | 11355078.18 |
| P27590 | Uromodulin OS=Rattus norvegicus OX=10116 GN=Umod PE=2 SV=1 | 71 | 32 | 102 | 14 | 62879065.73 | 7961604.927 | 70653129.11 |
| P63102 | 14-3-3 protein zeta/delta OS=Rattus norvegicus OX=10116 GN=Ywhaz PE=1 SV=1 | 27.8 | 55 | 106 | 8 | 27817540.75 | 12534601.09 | 137561049.3 |
| Q9ET61 | Complement component C1q receptor OS=Rattus norvegicus OX=10116 GN=Cd93 PE=1 SV=1 | 68.7 | 11 | 11 | 4 | 224206.4946 | 151777.8127 | 2498040.438 |
| P11232 | Thioredoxin OS=Rattus norvegicus OX=10116 GN=Txn PE=1 SV=2 | 11.7 | 43 | 20 | 4 | 16477427.85 | 2661898.108 | 20161438.07 |
| P48032 | Metalloproteinase inhibitor 3 OS=Rattus norvegicus OX=10116 GN=Timp3 PE=2 SV=1 | 24.2 | 29 | 21 | 5 | 786638.4384 | 655689.4887 | 4927569.207 |
| P09495 | Tropomyosin alpha-4 chain OS=Rattus norvegicus OX=10116 GN=Tpm4 PE=1 SV=3 | 28.5 | 37 | 66 | 6 | 11055809.2 | 2105484.811 | 25810380.2 |
| P34901 | Syndecan-4 OS=Rattus norvegicus OX=10116 GN=Sdc4 PE=1 SV=1 | 21.9 | 27 | 43 | 4 | 16561847.88 | 6663690.259 | 27852453.86 |
| P51647 | Retinal dehydrogenase 1 OS=Rattus norvegicus OX=10116 GN=Aldh1a1 PE=1 SV=3 | 54.4 | 37 | 29 | 9 | 736518.6268 | 523874.6446 | 9798065.031 |
| Q9R1T5 | Aspartoacylase OS=Rattus norvegicus OX=10116 GN=Aspa PE=1 SV=2 | 35.3 | 60 | 40 | 12 | 1291401.481 | 341846.1407 | 36974329.49 |
| P19468 | Glutamate--cysteine ligase catalytic subunit OS=Rattus norvegicus OX=10116 GN=Gclc PE=1 SV=2 | 72.6 | 46 | 85 | 21 | 39918859.1 | 3738185.115 | 70659378.74 |
| Q5XIF6 | Tubulin alpha-4A chain OS=Rattus norvegicus OX=10116 GN=Tuba4a PE=1 SV=1 | 49.9 | 46 | 94 | 6 | 2263655.534 | 1171920.283 | 10183112.26 |
| P70619 | Glutathione reductase (Fragment) OS=Rattus norvegicus OX=10116 GN=Gsr PE=2 SV=2 | 46.3 | 34 | 16 | 8 | 1191286.719 | 695106.3538 | 41529744.75 |
| P34067 | Proteasome subunit beta type-4 OS=Rattus norvegicus OX=10116 GN=Psmb4 PE=1 SV=2 | 29.2 | 17 | 13 | 3 | 3802488.618 | 55533.86025 | 3642491.516 |
| Q9QWN8 | Spectrin beta chain. non-erythrocytic 2 OS=Rattus norvegicus OX=10116 GN=Sptbn2 PE=1 SV=2 | 270.9 | 1 | 25 | 1 | 343610.6299 | 62401.90753 | 1472112.394 |
| P28064 | Proteasome subunit beta type-8 OS=Rattus norvegicus OX=10116 GN=Psmb8 PE=1 SV=3 | 30.6 | 27 | 20 | 7 | 2462334.14 | 79451.74593 | 1285360.813 |
| Q63207 | Coagulation factor X OS=Rattus norvegicus OX=10116 GN=F10 PE=2 SV=1 | 54.2 | 30 | 63 | 13 | 26091125.99 | 1818530.002 | 23192155.52 |
| Q63270 | Cytoplasmic aconitate hydratase OS=Rattus norvegicus OX=10116 GN=Aco1 PE=1 SV=1 | 98.1 | 33 | 60 | 20 | 7198016.628 | 1712819.861 | 52613008.18 |
| A7VJC2 | Heterogeneous nuclear ribonucleoproteins A2/B1 OS=Rattus norvegicus OX=10116 GN=Hnrnpa2b1 PE=1 SV=1 | 37.5 | 14 | 13 | 5 | 48744.01088 | 29915.35679 | 2862390.102 |
| O88600 | Heat shock 70 kDa protein 4 OS=Rattus norvegicus OX=10116 GN=Hspa4 PE=1 SV=1 | 94 | 42 | 87 | 22 | 10650435.05 | 719046.7187 | 19139895.93 |
| P97584 | Prostaglandin reductase 1 OS=Rattus norvegicus OX=10116 GN=Ptgr1 PE=2 SV=3 | 35.7 | 22 | 7 | 5 |  | 228975.7619 | 6974467.969 |
| Q6AYS7 | Aminoacylase-1A OS=Rattus norvegicus OX=10116 GN=Acy1a PE=1 SV=1 | 45.8 | 36 | 24 | 10 | 9255619.186 | 10538880.73 | 61099659.39 |
| P63004 | Platelet-activating factor acetylhydrolase IB subunit alpha OS=Rattus norvegicus OX=10116 GN=Pafah1b1 PE=1 SV=2 | 46.6 | 14 | 11 | 4 | 615468.6359 | 17263.62698 | 2997885.949 |
| P25093 | Fumarylacetoacetase OS=Rattus norvegicus OX=10116 GN=Fah PE=1 SV=1 | 45.9 | 46 | 39 | 11 | 4458478.177 | 450482.1176 | 145278660.4 |
| P40241 | CD9 antigen OS=Rattus norvegicus OX=10116 GN=Cd9 PE=1 SV=2 | 25.2 | 4 | 3 | 1 | 417694.9057 | 25472.87028 | 330218.0938 |
| P11348 | Dihydropteridine reductase OS=Rattus norvegicus OX=10116 GN=Qdpr PE=1 SV=1 | 25.5 | 41 | 13 | 6 | 3130762.989 | 383287.1374 | 41539826.06 |
| Q9JJ40 | Na(+)/H(+) exchange regulatory cofactor NHE-RF3 OS=Rattus norvegicus OX=10116 GN=Pdzk1 PE=1 SV=2 | 56.8 | 60 | 46 | 22 | 651093.9918 | 587100.9158 | 71503062.85 |
| B2RYG6 | Ubiquitin thioesterase OTUB1 OS=Rattus norvegicus OX=10116 GN=Otub1 PE=1 SV=1 | 31.3 | 7 | 1 | 1 |  | 19160.756 | 258516.9688 |
| Q9JHW0 | Proteasome subunit beta type-7 OS=Rattus norvegicus OX=10116 GN=Psmb7 PE=1 SV=1 | 29.9 | 9 | 12 | 4 | 2012445.736 | 8614.183958 | 1414399.523 |
| P46413 | Glutathione synthetase OS=Rattus norvegicus OX=10116 GN=Gss PE=1 SV=1 | 52.3 | 32 | 18 | 10 | 3502555.906 | 3355383.371 | 40554133.78 |
| Q9EPB1 | Dipeptidyl peptidase 2 OS=Rattus norvegicus OX=10116 GN=Dpp7 PE=1 SV=1 | 55.1 | 7 | 6 | 3 | 1064787.156 | 90588.52955 | 1665122.539 |
| P17220 | Proteasome subunit alpha type-2 OS=Rattus norvegicus OX=10116 GN=Psma2 PE=1 SV=3 | 25.9 | 38 | 28 | 6 | 2598153.402 | 784766.5742 | 2339043.359 |
| Q6P9T8 | Tubulin beta-4B chain OS=Rattus norvegicus OX=10116 GN=Tubb4b PE=1 SV=1 | 49.8 | 56 | 78 | 4 | 455158.1746 | 147059.2537 | 4702313.617 |
| P04797 | Glyceraldehyde-3-phosphate dehydrogenase OS=Rattus norvegicus OX=10116 GN=Gapdh PE=1 SV=3 | 35.8 | 48 | 96 | 11 | 8364264.148 | 2654761.893 | 37224202.5 |
| P46462 | Transitional endoplasmic reticulum ATPase OS=Rattus norvegicus OX=10116 GN=Vcp PE=1 SV=3 | 89.3 | 48 | 113 | 28 | 12928360.17 | 4083004.077 | 17830366.64 |
| P09034 | Argininosuccinate synthase OS=Rattus norvegicus OX=10116 GN=Ass1 PE=1 SV=1 | 46.5 | 49 | 79 | 16 | 3567833.861 | 1112486.162 | 23898628.75 |
| Q6XQN1 | Nicotinate phosphoribosyltransferase OS=Rattus norvegicus OX=10116 GN=Naprt PE=2 SV=1 | 58.5 | 38 | 41 | 12 | 824540.581 | 487989.789 | 36584520.87 |
| Q64604 | Receptor-type tyrosine-protein phosphatase F OS=Rattus norvegicus OX=10116 GN=Ptprf PE=2 SV=1 | 211.4 | 11 | 24 | 13 | 285658.4275 | 220192.4595 | 6901213.012 |
| P12928 | Pyruvate kinase PKLR OS=Rattus norvegicus OX=10116 GN=Pklr PE=1 SV=2 | 62.2 | 32 | 34 | 11 | 5556295.467 | 141531.1207 | 5288513.266 |
| P60901 | Proteasome subunit alpha type-6 OS=Rattus norvegicus OX=10116 GN=Psma6 PE=1 SV=1 | 27.4 | 46 | 48 | 9 | 13085429.09 | 2100794.644 | 13403399.25 |
| Q5XI22 | Acetyl-CoA acetyltransferase. cytosolic OS=Rattus norvegicus OX=10116 GN=Acat2 PE=1 SV=1 | 41.1 | 15 | 5 | 3 |  | 26718.50084 | 1166126.047 |
| P06761 | Endoplasmic reticulum chaperone BiP OS=Rattus norvegicus OX=10116 GN=Hspa5 PE=1 SV=1 | 72.3 | 20 | 50 | 10 | 5289709.016 | 267108.7431 | 16660916.52 |
| P48004 | Proteasome subunit alpha type-7 OS=Rattus norvegicus OX=10116 GN=Psma7 PE=1 SV=1 | 28.3 | 27 | 28 | 6 | 6217812.775 | 195592.977 | 4728364.203 |
| P61983 | 14-3-3 protein gamma OS=Rattus norvegicus OX=10116 GN=Ywhag PE=1 SV=2 | 28.3 | 39 | 54 | 4 | 859138.2876 | 66781.6829 | 8399273.156 |
| P14173 | Aromatic-L-amino-acid decarboxylase OS=Rattus norvegicus OX=10116 GN=Ddc PE=1 SV=1 | 54 | 30 | 22 | 10 | 1684176.247 | 495589.1692 | 41638547.74 |
| Q9R0T4 | Cadherin-1 OS=Rattus norvegicus OX=10116 GN=Cdh1 PE=1 SV=1 | 98.7 | 17 | 27 | 6 | 611778.2484 | 174205.3066 | 5557863.797 |
| P40307 | Proteasome subunit beta type-2 OS=Rattus norvegicus OX=10116 GN=Psmb2 PE=1 SV=1 | 22.9 | 33 | 39 | 7 | 6101427.34 | 140921.6813 | 5278970.605 |
| Q7TP54 | Rho family-interacting cell polarization regulator 2 OS=Rattus norvegicus OX=10116 GN=Ripor2 PE=1 SV=1 | 144.6 | 7 | 52 | 6 | 11949978.99 | 1009513.795 | 17055106.62 |
| P25113 | Phosphoglycerate mutase 1 OS=Rattus norvegicus OX=10116 GN=Pgam1 PE=1 SV=4 | 28.8 | 49 | 23 | 7 | 51526.76619 | 426728.2414 | 83914478.46 |
| Q6AYT0 | Quinone oxidoreductase OS=Rattus norvegicus OX=10116 GN=Cryz PE=2 SV=1 | 35 | 54 | 33 | 11 | 512142.8464 | 649967.8402 | 60104079.07 |
| Q6P6V0 | Glucose-6-phosphate isomerase OS=Rattus norvegicus OX=10116 GN=Gpi PE=1 SV=1 | 62.8 | 43 | 47 | 14 | 26576967.59 | 2392217.801 | 130554547.3 |
| P18421 | Proteasome subunit beta type-1 OS=Rattus norvegicus OX=10116 GN=Psmb1 PE=1 SV=3 | 26.5 | 43 | 56 | 8 | 9645235.692 | 482989.6099 | 7431135.166 |
| P97603 | Neogenin (Fragment) OS=Rattus norvegicus OX=10116 GN=Neo1 PE=1 SV=1 | 150.5 | 19 | 50 | 16 | 1156081.345 | 167392.8014 | 6599775.889 |
| Q64640 | Adenosine kinase OS=Rattus norvegicus OX=10116 GN=Adk PE=1 SV=3 | 40.1 | 26 | 7 | 6 | 468071.7341 | 120722.4043 | 3297286.902 |
| Q5U300 | Ubiquitin-like modifier-activating enzyme 1 OS=Rattus norvegicus OX=10116 GN=Uba1 PE=1 SV=1 | 117.7 | 51 | 129 | 35 | 48413225.36 | 5354125.697 | 74074217.75 |
| P63245 | Receptor of activated protein C kinase 1 OS=Rattus norvegicus OX=10116 GN=Rack1 PE=1 SV=3 | 35.1 | 23 | 17 | 4 | 104914.5454 | 23609.74373 | 1758106.02 |
| Q5RKI1 | Eukaryotic initiation factor 4A-II OS=Rattus norvegicus OX=10116 GN=Eif4a2 PE=1 SV=1 | 46.4 | 13 | 6 | 4 |  | 34891.74406 | 2567814.375 |
| Q6URK4 | Heterogeneous nuclear ribonucleoprotein A3 OS=Rattus norvegicus OX=10116 GN=Hnrnpa3 PE=1 SV=1 | 39.6 | 20 | 18 | 5 | 1810382.717 | 10649.31116 | 1826050.926 |
| P30904 | Macrophage migration inhibitory factor OS=Rattus norvegicus OX=10116 GN=Mif PE=1 SV=4 | 12.5 | 17 | 8 | 2 | 145859.9469 | 71975.23681 | 2438700.188 |
| P50137 | Transketolase OS=Rattus norvegicus OX=10116 GN=Tkt PE=1 SV=1 | 67.6 | 43 | 74 | 15 | 24711869.97 | 840481.2966 | 51471217.2 |
| P61459 | Pterin-4-alpha-carbinolamine dehydratase OS=Rattus norvegicus OX=10116 GN=Pcbd1 PE=1 SV=2 | 12 | 73 | 18 | 6 | 54476.79284 | 156573.5619 | 9908623.898 |
| P04904 | Glutathione S-transferase alpha-3 OS=Rattus norvegicus OX=10116 GN=Gsta3 PE=1 SV=3 | 25.3 | 38 | 22 | 4 | 50929.88673 | 78515.81549 | 5315510.461 |
| P34064 | Proteasome subunit alpha type-5 OS=Rattus norvegicus OX=10116 GN=Psma5 PE=1 SV=1 | 26.4 | 37 | 41 | 6 | 10407387.56 | 382171.1951 | 10231139.23 |
| P09606 | Glutamine synthetase OS=Rattus norvegicus OX=10116 GN=Glul PE=1 SV=3 | 42.2 | 13 | 7 | 3 | 425339.784 | 64659.89657 | 1323241.816 |
| Q9Z0W7 | Chloride intracellular channel protein 4 OS=Rattus norvegicus OX=10116 GN=Clic4 PE=1 SV=3 | 28.6 | 39 | 24 | 6 | 71116.71421 | 41211.28439 | 6753200.926 |
| P62986 | Ubiquitin-60S ribosomal protein L40 OS=Rattus norvegicus OX=10116 GN=Uba52 PE=1 SV=2 | 14.7 | 27 | 39 | 3 | 18346363.3 | 631275.5219 | 23895602.76 |
| P10760 | Adenosylhomocysteinase OS=Rattus norvegicus OX=10116 GN=Ahcy PE=1 SV=3 | 47.5 | 37 | 29 | 14 | 12067350.83 | 816939.8608 | 13245624.56 |
| Q642G2 | Sclerostin domain-containing protein 1 OS=Rattus norvegicus OX=10116 GN=Sostdc1 PE=2 SV=1 | 23.1 | 7 | 2 | 1 |  | 27020.08127 | 696185.5 |
| P06757 | Alcohol dehydrogenase 1 OS=Rattus norvegicus OX=10116 GN=Adh1 PE=1 SV=3 | 39.6 | 27 | 17 | 5 | 278720.9026 | 23491.52563 | 5693463.449 |
| P40112 | Proteasome subunit beta type-3 OS=Rattus norvegicus OX=10116 GN=Psmb3 PE=1 SV=1 | 22.9 | 34 | 24 | 5 | 1149529.517 | 67349.07632 | 2363478.32 |
| P18422 | Proteasome subunit alpha type-3 OS=Rattus norvegicus OX=10116 GN=Psma3 PE=1 SV=3 | 28.4 | 27 | 45 | 6 | 11961342.4 | 212402.9473 | 8707516.227 |
| Q4QQR9 | Protein MEMO1 OS=Rattus norvegicus OX=10116 GN=Memo1 PE=1 SV=1 | 33.7 | 35 | 24 | 7 | 325694.7706 | 25427.62244 | 1768854.457 |
| P23764 | Glutathione peroxidase 3 OS=Rattus norvegicus OX=10116 GN=Gpx3 PE=2 SV=2 | 25.4 | 50 | 1107 | 10 | 676099599.2 | 187619907.7 | 4531473312 |
| P04639 | Apolipoprotein A-I OS=Rattus norvegicus OX=10116 GN=Apoa1 PE=1 SV=2 | 30 | 57 | 155 | 14 | 67448543.39 | 2556163.439 | 51032263.78 |
| P62260 | 14-3-3 protein epsilon OS=Rattus norvegicus OX=10116 GN=Ywhae PE=1 SV=1 | 29.2 | 49 | 96 | 11 | 25573842.19 | 4786190.897 | 103795035.2 |
| P41562 | Isocitrate dehydrogenase [NADP] cytoplasmic OS=Rattus norvegicus OX=10116 GN=Idh1 PE=1 SV=1 | 46.7 | 48 | 71 | 17 | 760229.2384 | 1381643.667 | 155611225.1 |
| P05197 | Elongation factor 2 OS=Rattus norvegicus OX=10116 GN=Eef2 PE=1 SV=4 | 95.2 | 38 | 71 | 21 | 701733.3586 | 144154.2307 | 34261912.53 |
| Q5M876 | N-acyl-aromatic-L-amino acid amidohydrolase (carboxylate-forming) OS=Rattus norvegicus OX=10116 GN=Acy3 PE=1 SV=1 | 35.4 | 46 | 34 | 9 | 649208.7541 | 527704.1201 | 47705185.78 |
| P28073 | Proteasome subunit beta type-6 OS=Rattus norvegicus OX=10116 GN=Psmb6 PE=1 SV=3 | 25.3 | 19 | 26 | 5 | 4430961.926 | 52058.16448 | 3514489.914 |
| Q9EQS0 | Transaldolase OS=Rattus norvegicus OX=10116 GN=Taldo1 PE=1 SV=2 | 37.4 | 24 | 14 | 7 | 1645972.41 | 266239.5452 | 22719747.92 |
| P70712 | Kynureninase OS=Rattus norvegicus OX=10116 GN=Kynu PE=1 SV=2 | 52.4 | 30 | 16 | 11 |  | 42449.05376 | 17674669.78 |
| P85108 | Tubulin beta-2A chain OS=Rattus norvegicus OX=10116 GN=Tubb2a PE=1 SV=1 | 49.9 | 47 | 74 | 2 | 85618.94264 | 11967.05464 | 502961.3984 |
| Q63610 | Tropomyosin alpha-3 chain OS=Rattus norvegicus OX=10116 GN=Tpm3 PE=1 SV=2 | 29 | 31 | 62 | 8 | 24430976.25 | 2219482.197 | 66314846.93 |
| Q8CG45 | Aflatoxin B1 aldehyde reductase member 2 OS=Rattus norvegicus OX=10116 GN=Akr7a2 PE=1 SV=2 | 40.6 | 31 | 79 | 7 | 9343495.857 | 1877599.65 | 60543197.93 |
| P38918 | Aflatoxin B1 aldehyde reductase member 3 OS=Rattus norvegicus OX=10116 GN=Akr7a3 PE=1 SV=2 | 36.7 | 32 | 43 | 7 | 554615.2723 | 405696.3986 | 12056173.48 |
| Q9WUW8 | Sulfotransferase 1C2 OS=Rattus norvegicus OX=10116 GN=Sult1c2 PE=2 SV=1 | 34.7 | 36 | 20 | 8 | 334403.7498 | 180479.8138 | 25165292.38 |
| P48508 | Glutamate--cysteine ligase regulatory subunit OS=Rattus norvegicus OX=10116 GN=Gclm PE=1 SV=1 | 30.5 | 31 | 15 | 6 | 5406100.764 | 116353.334 | 16803439.13 |
| P21670 | Proteasome subunit alpha type-4 OS=Rattus norvegicus OX=10116 GN=Psma4 PE=1 SV=1 | 29.5 | 30 | 21 | 7 | 5880216.745 | 125700.7849 | 4833639.337 |
| P02650 | Apolipoprotein E OS=Rattus norvegicus OX=10116 GN=Apoe PE=1 SV=2 | 35.7 | 33 | 180 | 10 | 435659684.9 | 4070876.071 | 225182052.5 |
| Q9QXQ0 | Alpha-actinin-4 OS=Rattus norvegicus OX=10116 GN=Actn4 PE=1 SV=2 | 104.8 | 58 | 203 | 26 | 4933478.605 | 335766.3338 | 33791654.63 |
| P16617 | Phosphoglycerate kinase 1 OS=Rattus norvegicus OX=10116 GN=Pgk1 PE=1 SV=2 | 44.5 | 29 | 24 | 8 | 15353.52485 | 27479.338 | 6746201.355 |
| P42123 | L-lactate dehydrogenase B chain OS=Rattus norvegicus OX=10116 GN=Ldhb PE=1 SV=2 | 36.6 | 51 | 48 | 12 | 82724.62932 | 95373.29836 | 27242410.34 |
| P18420 | Proteasome subunit alpha type-1 OS=Rattus norvegicus OX=10116 GN=Psma1 PE=1 SV=2 | 29.5 | 41 | 62 | 9 | 10402204.6 | 387126.8779 | 11284741.17 |
| P05426 | 60S ribosomal protein L7 OS=Rattus norvegicus OX=10116 GN=Rpl7 PE=1 SV=2 | 30.3 | 12 | 11 | 2 | 18774.7823 | 2487.519028 | 275049.5762 |
| Q9JJ19 | Na(+)/H(+) exchange regulatory cofactor NHE-RF1 OS=Rattus norvegicus OX=10116 GN=Slc9a3r1 PE=1 SV=3 | 38.8 | 37 | 32 | 10 | 707414.6336 | 344820.077 | 74768729.89 |
| Q5I0D7 | Xaa-Pro dipeptidase OS=Rattus norvegicus OX=10116 GN=Pepd PE=2 SV=1 | 54.7 | 38 | 69 | 14 | 8056699.702 | 2393625.058 | 137951773.5 |
| P48500 | Triosephosphate isomerase OS=Rattus norvegicus OX=10116 GN=Tpi1 PE=1 SV=2 | 26.8 | 68 | 73 | 12 | 16530029.25 | 1511268.549 | 183732227.7 |
| Q9Z339 | Glutathione S-transferase omega-1 OS=Rattus norvegicus OX=10116 GN=Gsto1 PE=1 SV=2 | 27.7 | 50 | 31 | 8 | 7640496.474 | 619365.9858 | 39833612.77 |
| Q9JLZ1 | Glutaredoxin-3 OS=Rattus norvegicus OX=10116 GN=Glrx3 PE=1 SV=2 | 37.8 | 11 | 7 | 3 |  | 15755.02689 | 1580346.875 |
| P19804 | Nucleoside diphosphate kinase B OS=Rattus norvegicus OX=10116 GN=Nme2 PE=1 SV=1 | 17.3 | 68 | 126 | 3 | 19007538.21 | 1370665.501 | 85730910.66 |
| P35213 | 14-3-3 protein beta/alpha OS=Rattus norvegicus OX=10116 GN=Ywhab PE=1 SV=3 | 28 | 48 | 62 | 5 | 1762221.595 | 216814.5792 | 13714060.15 |
| O88989 | Malate dehydrogenase. cytoplasmic OS=Rattus norvegicus OX=10116 GN=Mdh1 PE=1 SV=3 | 36.5 | 41 | 41 | 11 | 50557668.19 | 7157014.866 | 440961391.1 |
| Q711G3 | Isoamyl acetate-hydrolyzing esterase 1 homolog OS=Rattus norvegicus OX=10116 GN=Iah1 PE=2 SV=2 | 28 | 39 | 35 | 6 | 802407.3753 | 249530.5505 | 34375806.65 |
| O89049 | Thioredoxin reductase 1. cytoplasmic OS=Rattus norvegicus OX=10116 GN=Txnrd1 PE=1 SV=5 | 54.6 | 37 | 45 | 12 | 1033467.102 | 57928.96678 | 29365737.03 |
| P18418 | Calreticulin OS=Rattus norvegicus OX=10116 GN=Calr PE=1 SV=1 | 48 | 34 | 40 | 10 | 7843129.812 | 145832.3297 | 22434111.97 |
| P00884 | Fructose-bisphosphate aldolase B OS=Rattus norvegicus OX=10116 GN=Aldob PE=1 SV=2 | 39.6 | 62 | 227 | 15 | 20707496.55 | 4289564.383 | 1075707228 |
| P69897 | Tubulin beta-5 chain OS=Rattus norvegicus OX=10116 GN=Tubb5 PE=1 SV=1 | 49.6 | 55 | 85 | 2 |  |  | 243486.5391 |
| Q6JE36 | Protein NDRG1 OS=Rattus norvegicus OX=10116 GN=Ndrg1 PE=1 SV=1 | 42.9 | 33 | 57 | 8 | 1272196.782 | 43347.29384 | 35994448 |
| Q8VIF7 | Methanethiol oxidase OS=Rattus norvegicus OX=10116 GN=Selenbp1 PE=1 SV=1 | 52.5 | 42 | 52 | 14 | 40789523.16 | 148670.2795 | 32949432.96 |
| P68255 | 14-3-3 protein theta OS=Rattus norvegicus OX=10116 GN=Ywhaq PE=1 SV=1 | 27.8 | 42 | 50 | 5 | 1677991.61 | 92257.82461 | 26482318.5 |
| Q00657 | Chondroitin sulfate proteoglycan 4 OS=Rattus norvegicus OX=10116 GN=Cspg4 PE=1 SV=2 | 251.8 | 7 | 16 | 12 | 6613.511521 |  | 895270.0195 |
| P97571 | Calpain-1 catalytic subunit OS=Rattus norvegicus OX=10116 GN=Capn1 PE=1 SV=1 | 82.1 | 19 | 35 | 11 | 1063236.42 |  | 2080545.719 |
| P04692 | Tropomyosin alpha-1 chain OS=Rattus norvegicus OX=10116 GN=Tpm1 PE=1 SV=3 | 32.7 | 17 | 40 | 3 | 5344219.451 | 47130.56574 | 25867757.89 |
| D4A1J4 | 3-hydroxybutyrate dehydrogenase type 2 OS=Rattus norvegicus OX=10116 GN=Bdh2 PE=3 SV=2 | 26.6 | 45 | 10 | 8 | 73532.56318 |  | 25526762.31 |
| Q7TQ94 | Deaminated glutathione amidase OS=Rattus norvegicus OX=10116 GN=Nit1 PE=2 SV=2 | 36.1 | 35 | 13 | 7 | 77034.8731 |  | 11223695.47 |
| P62959 | Histidine triad nucleotide-binding protein 1 OS=Rattus norvegicus OX=10116 GN=Hint1 PE=1 SV=5 | 13.8 | 71 | 23 | 5 | 45070.11225 |  | 9391600.031 |
| P04176 | Phenylalanine-4-hydroxylase OS=Rattus norvegicus OX=10116 GN=Pah PE=1 SV=3 | 51.8 | 28 | 11 | 8 |  |  | 11729195.88 |
| Q66X93 | Staphylococcal nuclease domain-containing protein 1 OS=Rattus norvegicus OX=10116 GN=Snd1 PE=1 SV=1 | 101.9 | 14 | 9 | 7 |  |  | 2193555.484 |
| P19139 | Casein kinase II subunit alpha OS=Rattus norvegicus OX=10116 GN=Csnk2a1 PE=1 SV=2 | 45 | 26 | 9 | 7 |  |  | 1396769.773 |
| Q8VI04 | Isoaspartyl peptidase/L-asparaginase OS=Rattus norvegicus OX=10116 GN=Asrgl1 PE=1 SV=1 | 34.4 | 31 | 10 | 7 |  |  | 13439228.75 |
| P34058 | Heat shock protein HSP 90-beta OS=Rattus norvegicus OX=10116 GN=Hsp90ab1 PE=1 SV=4 | 83.2 | 15 | 13 | 8 |  |  | 1631449.344 |
| P35053 | Glypican-1 OS=Rattus norvegicus OX=10116 GN=Gpc1 PE=1 SV=1 | 61.7 | 20 | 9 | 8 | 78215.65216 |  | 1242847.666 |
| Q01129 | Decorin OS=Rattus norvegicus OX=10116 GN=Dcn PE=1 SV=1 | 39.8 | 14 | 17 | 5 | 503439.694 |  | 4310444.219 |
| Q562C6 | Leucine zipper transcription factor-like protein 1 OS=Rattus norvegicus OX=10116 GN=Lztfl1 PE=2 SV=1 | 34.6 | 23 | 5 | 5 |  |  | 1420558.797 |
| P13221 | Aspartate aminotransferase. cytoplasmic OS=Rattus norvegicus OX=10116 GN=Got1 PE=1 SV=3 | 46.4 | 15 | 6 | 4 |  |  | 4621419.063 |
| Q63798 | Proteasome activator complex subunit 2 OS=Rattus norvegicus OX=10116 GN=Psme2 PE=2 SV=3 | 26.8 | 28 | 5 | 4 |  |  | 2446212.563 |
| O35077 | Glycerol-3-phosphate dehydrogenase [NAD(+)]. cytoplasmic OS=Rattus norvegicus OX=10116 GN=Gpd1 PE=1 SV=4 | 37.4 | 20 | 18 | 4 | 10638.46439 |  | 1524101.451 |
| P49911 | Acidic leucine-rich nuclear phosphoprotein 32 family member A OS=Rattus norvegicus OX=10116 GN=Anp32a PE=2 SV=1 | 28.5 | 16 | 5 | 4 |  |  | 10945409.75 |
| P06765 | Platelet factor 4 OS=Rattus norvegicus OX=10116 GN=Pf4 PE=1 SV=1 | 11.3 | 38 | 22 | 4 |  | 340214.3694 | 87402600.61 |
| Q6IG05 | Keratin. type II cytoskeletal 75 OS=Rattus norvegicus OX=10116 GN=Krt75 PE=3 SV=2 | 59 | 8 | 49 | 1 |  |  | 135408.375 |
| P24054 | SPARC-like protein 1 OS=Rattus norvegicus OX=10116 GN=Sparcl1 PE=1 SV=1 | 70.6 | 10 | 7 | 5 | 13986.2183 |  | 619374.0938 |
| P31977 | Ezrin OS=Rattus norvegicus OX=10116 GN=Ezr PE=1 SV=3 | 69.3 | 9 | 8 | 5 |  |  | 5451347.5 |
| D3ZW55 | Inosine triphosphate pyrophosphatase OS=Rattus norvegicus OX=10116 GN=Itpa PE=3 SV=1 | 21.9 | 28 | 7 | 4 | 1240438.775 |  | 4612845.063 |
| Q08163 | Adenylyl cyclase-associated protein 1 OS=Rattus norvegicus OX=10116 GN=Cap1 PE=1 SV=3 | 51.6 | 4 | 14 | 1 | 123496.2798 |  | 330779.4492 |
| Q6IG03 | Keratin. type II cytoskeletal 73 OS=Rattus norvegicus OX=10116 GN=Krt73 PE=1 SV=1 | 60.3 | 8 | 46 | 2 |  |  | 73856.58594 |
| Q4TU93 | C-type mannose receptor 2 OS=Rattus norvegicus OX=10116 GN=Mrc2 PE=1 SV=1 | 166.9 | 4 | 7 | 4 | 914773.357 |  | 119680.1719 |
| Q5M819 | Phosphoserine phosphatase OS=Rattus norvegicus OX=10116 GN=Psph PE=2 SV=1 | 25 | 17 | 4 | 3 | 968391.7439 |  | 6240684.063 |
| Q3T1K5 | F-actin-capping protein subunit alpha-2 OS=Rattus norvegicus OX=10116 GN=Capza2 PE=1 SV=1 | 32.9 | 29 | 6 | 4 | 19231.63944 |  | 956986.3281 |
| Q4KM35 | Proteasome subunit beta type-10 OS=Rattus norvegicus OX=10116 GN=Psmb10 PE=2 SV=1 | 29 | 12 | 4 | 2 | 203126.3245 |  | 253821.6094 |
| Q9QVC8 | Peptidyl-prolyl cis-trans isomerase FKBP4 OS=Rattus norvegicus OX=10116 GN=Fkbp4 PE=1 SV=3 | 51.4 | 11 | 4 | 3 |  |  | 1242435.25 |
| P46953 | 3-hydroxyanthranilate 3.4-dioxygenase OS=Rattus norvegicus OX=10116 GN=Haao PE=1 SV=2 | 32.6 | 19 | 13 | 4 | 5667.719866 |  | 2990934.369 |
| P16638 | ATP-citrate synthase OS=Rattus norvegicus OX=10116 GN=Acly PE=1 SV=1 | 120.6 | 5 | 7 | 4 | 6635.200119 |  | 5748.86084 |
| Q8R2H2 | Integrin beta-3 OS=Rattus norvegicus OX=10116 GN=Itgb3 PE=2 SV=1 | 86.9 | 7 | 8 | 4 | 156547.8012 |  | 163352.2197 |
| P04182 | Ornithine aminotransferase. mitochondrial OS=Rattus norvegicus OX=10116 GN=Oat PE=1 SV=1 | 48.3 | 12 | 6 | 4 |  |  | 1880749.875 |
| Q920A6 | Retinoid-inducible serine carboxypeptidase OS=Rattus norvegicus OX=10116 GN=Scpep1 PE=2 SV=1 | 51.1 | 7 | 5 | 2 | 371794.2043 |  | 961783.1563 |
| O88767 | Protein/nucleic acid deglycase DJ-1 OS=Rattus norvegicus OX=10116 GN=Park7 PE=1 SV=1 | 20 | 43 | 10 | 5 |  |  | 4565982.953 |
| Q63598 | Plastin-3 OS=Rattus norvegicus OX=10116 GN=Pls3 PE=1 SV=2 | 70.6 | 9 | 9 | 5 | 1434989.993 |  | 9202631.938 |
| Q9QYU4 | Ketimine reductase mu-crystallin OS=Rattus norvegicus OX=10116 GN=Crym PE=1 SV=1 | 33.5 | 17 | 10 | 3 |  |  | 321631.0723 |
| P14141 | Carbonic anhydrase 3 OS=Rattus norvegicus OX=10116 GN=Ca3 PE=1 SV=3 | 29.4 | 14 | 11 | 3 |  |  | 126719.5059 |
| Q9EST6 | Acidic leucine-rich nuclear phosphoprotein 32 family member B OS=Rattus norvegicus OX=10116 GN=Anp32b PE=1 SV=1 | 31 | 17 | 4 | 3 |  |  | 450253.8594 |
| P18757 | Cystathionine gamma-lyase OS=Rattus norvegicus OX=10116 GN=Cth PE=1 SV=2 | 43.6 | 11 | 4 | 3 |  |  | 2082674.828 |
| P27867 | Sorbitol dehydrogenase OS=Rattus norvegicus OX=10116 GN=Sord PE=1 SV=4 | 38.2 | 14 | 11 | 4 | 2917505.358 |  | 1774998.582 |
| P07150 | Annexin A1 OS=Rattus norvegicus OX=10116 GN=Anxa1 PE=1 SV=2 | 38.8 | 15 | 4 | 4 |  |  | 302232.3281 |
| Q5XIE0 | Acidic leucine-rich nuclear phosphoprotein 32 family member E OS=Rattus norvegicus OX=10116 GN=Anp32e PE=2 SV=1 | 29.4 | 14 | 5 | 2 |  |  | 449684.0938 |
| Q6I7R3 | Isochorismatase domain-containing protein 1 OS=Rattus norvegicus OX=10116 GN=Isoc1 PE=1 SV=1 | 32 | 16 | 3 | 3 |  |  | 3501115.906 |
| P24155 | Thimet oligopeptidase OS=Rattus norvegicus OX=10116 GN=Thop1 PE=1 SV=4 | 78.3 | 8 | 4 | 4 | 535925.2845 |  | 2378351.938 |
| Q9R085 | Ubiquitin carboxyl-terminal hydrolase 15 OS=Rattus norvegicus OX=10116 GN=Usp15 PE=1 SV=1 | 109.2 | 6 | 6 | 5 | 70663.45304 |  | 175908.8418 |
| Q5ZQU0 | Sushi. nidogen and EGF-like domain-containing protein 1 OS=Rattus norvegicus OX=10116 GN=Sned1 PE=2 SV=2 | 151.3 | 3 | 3 | 3 |  |  | 175454.8164 |
| D4ACX8 | Protocadherin-16 OS=Rattus norvegicus OX=10116 GN=Dchs1 PE=3 SV=2 | 345.8 | 1 | 3 | 3 |  |  | 102087.248 |
| P98166 | Very low-density lipoprotein receptor OS=Rattus norvegicus OX=10116 GN=Vldlr PE=2 SV=1 | 96.5 | 4 | 3 | 2 | 26022.67144 |  | 89141.15234 |
| Q5XI32 | F-actin-capping protein subunit beta OS=Rattus norvegicus OX=10116 GN=Capzb PE=1 SV=1 | 30.6 | 21 | 5 | 4 |  |  | 1213160.063 |
| P27605 | Hypoxanthine-guanine phosphoribosyltransferase OS=Rattus norvegicus OX=10116 GN=Hprt1 PE=1 SV=1 | 24.5 | 18 | 6 | 3 | 9449860.077 |  | 4247784.625 |
| P01041 | Cystatin-B OS=Rattus norvegicus OX=10116 GN=Cstb PE=1 SV=1 | 11.2 | 35 | 4 | 3 |  |  | 3544136.875 |
| P00502 | Glutathione S-transferase alpha-1 OS=Rattus norvegicus OX=10116 GN=Gsta1 PE=1 SV=3 | 25.6 | 17 | 8 | 2 |  |  | 1312456.75 |
| Q497C3 | Methyltransferase-like 26 OS=Rattus norvegicus OX=10116 GN=Mettl26 PE=2 SV=2 | 22.6 | 13 | 6 | 2 | 134564.8545 |  | 545251.2188 |
| Q63945 | Protein SET OS=Rattus norvegicus OX=10116 GN=Set PE=2 SV=2 | 33.4 | 15 | 2 | 2 |  |  | 599119.1563 |
| Q66H86 | Olfactomedin-like protein 1 OS=Rattus norvegicus OX=10116 GN=Olfml1 PE=2 SV=1 | 45.6 | 17 | 8 | 6 | 338626.8426 |  | 1928746.063 |
| P61203 | COP9 signalosome complex subunit 2 OS=Rattus norvegicus OX=10116 GN=Cops2 PE=1 SV=1 | 51.6 | 9 | 4 | 3 | 16466.24241 |  | 256281.0391 |
| Q07936 | Annexin A2 OS=Rattus norvegicus OX=10116 GN=Anxa2 PE=1 SV=2 | 38.7 | 12 | 3 | 3 |  |  | 302707.5313 |
| Q6AXR4 | Beta-hexosaminidase subunit beta OS=Rattus norvegicus OX=10116 GN=Hexb PE=2 SV=1 | 61.5 | 9 | 4 | 3 | 494365.4566 |  | 237354.75 |
| P97523 | Hepatocyte growth factor receptor OS=Rattus norvegicus OX=10116 GN=Met PE=1 SV=1 | 153.8 | 3 | 3 | 3 | 89486.69941 |  | 595185.1875 |
| P45592 | Cofilin-1 OS=Rattus norvegicus OX=10116 GN=Cfl1 PE=1 SV=3 | 18.5 | 15 | 5 | 2 |  |  | 205653.7441 |
| Q4KMA2 | UV excision repair protein RAD23 homolog B OS=Rattus norvegicus OX=10116 GN=Rad23b PE=1 SV=1 | 43.5 | 7 | 2 | 2 | 182237.2729 |  | 387184 |
| Q9QXN4 | Inositol oxygenase OS=Rattus norvegicus OX=10116 GN=Miox PE=1 SV=2 | 33.2 | 19 | 5 | 4 |  |  | 273348.3223 |
| Q8K4F7 | m7GpppX diphosphatase OS=Rattus norvegicus OX=10116 GN=Dcps PE=2 SV=1 | 38.7 | 10 | 2 | 2 |  |  | 637604.2188 |
| P50475 | Alanine--tRNA ligase. cytoplasmic OS=Rattus norvegicus OX=10116 GN=Aars PE=1 SV=3 | 106.7 | 6 | 2 | 2 |  |  | 55180.44531 |
| P27952 | 40S ribosomal protein S2 OS=Rattus norvegicus OX=10116 GN=Rps2 PE=1 SV=1 | 31.2 | 9 | 5 | 2 |  |  | 97493.89063 |
| P67874 | Casein kinase II subunit beta OS=Rattus norvegicus OX=10116 GN=Csnk2b PE=1 SV=1 | 24.9 | 12 | 2 | 2 |  |  | 434692.2813 |
| P07379 | Phosphoenolpyruvate carboxykinase. cytosolic [GTP] OS=Rattus norvegicus OX=10116 GN=Pck1 PE=1 SV=1 | 69.4 | 4 | 4 | 2 |  |  | 1271252.555 |
| P26772 | 10 kDa heat shock protein. mitochondrial OS=Rattus norvegicus OX=10116 GN=Hspe1 PE=1 SV=3 | 10.9 | 25 | 2 | 2 |  |  | 2711109.5 |
| Q6AY86 | Vacuolar protein sorting-associated protein 26A OS=Rattus norvegicus OX=10116 GN=Vps26a PE=1 SV=1 | 38.1 | 9 | 2 | 2 |  |  | 170892.75 |
| A2RUV9 | Adipocyte enhancer-binding protein 1 OS=Rattus norvegicus OX=10116 GN=Aebp1 PE=2 SV=1 | 128 | 4 | 4 | 3 |  |  | 384612.2969 |
| O54975 | Xaa-Pro aminopeptidase 1 OS=Rattus norvegicus OX=10116 GN=Xpnpep1 PE=1 SV=1 | 69.6 | 4 | 3 | 2 |  |  | 1807000.813 |
| O08557 | N(G).N(G)-dimethylarginine dimethylaminohydrolase 1 OS=Rattus norvegicus OX=10116 GN=Ddah1 PE=1 SV=3 | 31.4 | 14 | 3 | 3 |  |  | 3745173.813 |
| P27274 | CD59 glycoprotein OS=Rattus norvegicus OX=10116 GN=Cd59 PE=1 SV=2 | 13.8 | 17 | 5 | 2 | 38353.64278 |  | 172910.1875 |
| Q08415 | Kynurenine--oxoglutarate transaminase 1. mitochondrial OS=Rattus norvegicus OX=10116 GN=Kyat1 PE=1 SV=1 | 51.6 | 9 | 3 | 2 |  |  | 711924.0859 |
| Q63525 | Nuclear migration protein nudC OS=Rattus norvegicus OX=10116 GN=Nudc PE=1 SV=1 | 38.4 | 5 | 3 | 1 | 19585.12566 |  | 66413.9375 |
| Q62598 | Dentin sialophosphoprotein OS=Rattus norvegicus OX=10116 GN=Dspp PE=1 SV=2 | 70.1 | 2 | 3 | 1 |  |  | 59301.75391 |
| P04636 | Malate dehydrogenase. mitochondrial OS=Rattus norvegicus OX=10116 GN=Mdh2 PE=1 SV=2 | 35.7 | 6 | 3 | 1 | 91010.85383 |  | 25940.52734 |
| Q62812 | Myosin-9 OS=Rattus norvegicus OX=10116 GN=Myh9 PE=1 SV=3 | 226.2 | 1 | 2 | 1 |  |  | 100229.4961 |
| P81799 | N-acetyl-D-glucosamine kinase OS=Rattus norvegicus OX=10116 GN=Nagk PE=1 SV=4 | 37.2 | 9 | 2 | 2 |  |  | 1752827.25 |
| Q9WTQ2 | Podocalyxin OS=Rattus norvegicus OX=10116 GN=Podxl PE=1 SV=2 | 51.6 | 7 | 3 | 2 |  |  | 263449.6875 |
| Q9QZA2 | Programmed cell death 6-interacting protein OS=Rattus norvegicus OX=10116 GN=Pdcd6ip PE=1 SV=2 | 96.6 | 3 | 2 | 2 |  |  | 550159.5313 |
| P04256 | Heterogeneous nuclear ribonucleoprotein A1 OS=Rattus norvegicus OX=10116 GN=Hnrnpa1 PE=1 SV=3 | 34.2 | 5 | 4 | 1 |  |  | 293771.8438 |
| P62703 | 40S ribosomal protein S4. X isoform OS=Rattus norvegicus OX=10116 GN=Rps4x PE=2 SV=2 | 29.6 | 7 | 3 | 2 |  |  | 83286.31738 |
| P12001 | 60S ribosomal protein L18 OS=Rattus norvegicus OX=10116 GN=Rpl18 PE=1 SV=2 | 21.6 | 7 | 2 | 1 |  |  | 47343.64453 |
| Q03248 | Beta-ureidopropionase OS=Rattus norvegicus OX=10116 GN=Upb1 PE=1 SV=1 | 44 | 5 | 1 | 1 |  |  | 1924591.375 |
| P57097 | Tyrosine-protein kinase Mer OS=Rattus norvegicus OX=10116 GN=Mertk PE=2 SV=1 | 109.4 | 2 | 2 | 1 | 309811.9839 |  | 241207.4375 |
| Q62745 | CD81 antigen OS=Rattus norvegicus OX=10116 GN=Cd81 PE=1 SV=1 | 25.9 | 7 | 2 | 1 |  |  | 144748.8438 |
| D4A1R8 | Copine-1 OS=Rattus norvegicus OX=10116 GN=Cpne1 PE=1 SV=1 | 58.8 | 3 | 4 | 2 | 26720.72427 |  | 243180.2129 |
| Q64119 | Myosin light polypeptide 6 OS=Rattus norvegicus OX=10116 GN=Myl6 PE=1 SV=3 | 17 | 11 | 4 | 1 | 25765.83589 |  | 307771.0742 |
| P63039 | 60 kDa heat shock protein. mitochondrial OS=Rattus norvegicus OX=10116 GN=Hspd1 PE=1 SV=1 | 60.9 | 4 | 4 | 1 |  |  | 49050.31641 |
| P62246 | 40S ribosomal protein S15a OS=Rattus norvegicus OX=10116 GN=Rps15a PE=1 SV=2 | 14.8 | 13 | 3 | 2 |  |  | 170688.7617 |
| P36970 | Phospholipid hydroperoxide glutathione peroxidase OS=Rattus norvegicus OX=10116 GN=Gpx4 PE=1 SV=3 | 22.2 | 13 | 3 | 2 | 70043.37371 |  | 353471.1953 |
| Q5I0D5 | Phospholysine phosphohistidine inorganic pyrophosphate phosphatase OS=Rattus norvegicus OX=10116 GN=Lhpp PE=2 SV=1 | 29.2 | 8 | 1 | 1 |  |  | 218414.4375 |
| D3ZHA0 | Filamin-C OS=Rattus norvegicus OX=10116 GN=Flnc PE=1 SV=1 | 290.8 | 0 | 2 | 1 |  |  | 352312.3203 |
| P62890 | 60S ribosomal protein L30 OS=Rattus norvegicus OX=10116 GN=Rpl30 PE=3 SV=2 | 12.8 | 27 | 3 | 2 | 313924.0162 |  | 117500.3018 |
| P31394 | Vitamin K-dependent protein C OS=Rattus norvegicus OX=10116 GN=Proc PE=2 SV=1 | 51.9 | 4 | 2 | 2 | 137606.8186 |  | 1217210.953 |
| P30713 | Glutathione S-transferase theta-2 OS=Rattus norvegicus OX=10116 GN=Gstt2 PE=1 SV=3 | 27.4 | 9 | 5 | 2 | 11748.97424 |  | 1320811.504 |
| O55145 | Fractalkine OS=Rattus norvegicus OX=10116 GN=Cx3cl1 PE=2 SV=3 | 42 | 10 | 7 | 2 | 866492.6738 |  | 1108163.754 |
| Q68G31 | Phenazine biosynthesis-like domain-containing protein OS=Rattus norvegicus OX=10116 GN=Pbld PE=2 SV=1 | 31.7 | 11 | 3 | 2 |  |  | 602087.2813 |
| Q5XI43 | Matrix remodeling-associated protein 8 OS=Rattus norvegicus OX=10116 GN=Mxra8 PE=1 SV=1 | 42.7 | 3 | 1 | 1 |  |  | 540855.25 |
| Q4V898 | RNA-binding motif protein. X chromosome OS=Rattus norvegicus OX=10116 GN=Rbmx PE=1 SV=1 | 42.2 | 4 | 2 | 1 |  |  | 170189.8008 |
| P51607 | N-acylglucosamine 2-epimerase OS=Rattus norvegicus OX=10116 GN=Renbp PE=2 SV=2 | 49.6 | 3 | 1 | 1 |  |  | 164281.5938 |
| Q63413 | Spliceosome RNA helicase Ddx39b OS=Rattus norvegicus OX=10116 GN=Ddx39b PE=2 SV=3 | 49 | 2 | 1 | 1 |  |  | 521047 |
| P62856 | 40S ribosomal protein S26 OS=Rattus norvegicus OX=10116 GN=Rps26 PE=3 SV=3 | 13 | 21 | 3 | 2 |  |  | 238066.5352 |
| P62278 | 40S ribosomal protein S13 OS=Rattus norvegicus OX=10116 GN=Rps13 PE=1 SV=2 | 17.2 | 11 | 3 | 2 | 29422.43359 |  | 271876.9844 |
| P04638 | Apolipoprotein A-II OS=Rattus norvegicus OX=10116 GN=Apoa2 PE=2 SV=1 | 11.4 | 11 | 3 | 1 | 1026884.447 |  | 242763.0469 |
| P15943 | Amyloid-like protein 2 OS=Rattus norvegicus OX=10116 GN=Aplp2 PE=1 SV=2 | 86.8 | 3 | 1 | 1 |  |  | 24020.74414 |
| F1LMZ8 | 26S proteasome non-ATPase regulatory subunit 11 OS=Rattus norvegicus OX=10116 GN=Psmd11 PE=1 SV=2 | 47.4 | 3 | 3 | 1 |  |  | 20015.08008 |
| Q924K2 | FAS-associated factor 1 OS=Rattus norvegicus OX=10116 GN=Faf1 PE=1 SV=1 | 73.7 | 2 | 1 | 1 |  |  | 23239.31445 |
| Q6AY82 | Endomucin OS=Rattus norvegicus OX=10116 GN=Emcn PE=2 SV=1 | 26.5 | 6 | 3 | 1 |  |  | 646524.6563 |
| P97608 | 5-oxoprolinase OS=Rattus norvegicus OX=10116 GN=Oplah PE=1 SV=2 | 137.6 | 2 | 2 | 2 |  |  | 168696.6797 |
| P17246 | Transforming growth factor beta-1 proprotein OS=Rattus norvegicus OX=10116 GN=Tgfb1 PE=1 SV=1 | 44.3 | 3 | 1 | 1 |  |  | 77231.07813 |
| E9PU28 | Inosine-5'-monophosphate dehydrogenase 2 OS=Rattus norvegicus OX=10116 GN=Impdh2 PE=1 SV=1 | 55.8 | 4 | 2 | 1 | 68955.64383 |  | 51650.40234 |
| Q8CIZ5 | Deleted in malignant brain tumors 1 protein OS=Rattus norvegicus OX=10116 GN=Dmbt1 PE=1 SV=1 | 155.6 | 1 | 3 | 1 | 36968.08821 |  | 61144.65234 |
| Q9JK72 | Copper chaperone for superoxide dismutase OS=Rattus norvegicus OX=10116 GN=Ccs PE=1 SV=1 | 28.9 | 9 | 1 | 1 |  |  | 18156.13477 |
| P97834 | COP9 signalosome complex subunit 1 OS=Rattus norvegicus OX=10116 GN=Gps1 PE=1 SV=1 | 53.4 | 4 | 2 | 2 |  |  | 214560.4102 |
| Q9WU49 | Calcium-regulated heat stable protein 1 OS=Rattus norvegicus OX=10116 GN=Carhsp1 PE=1 SV=1 | 15.9 | 11 | 3 | 1 | 139852.2405 |  | 708879.3281 |
| P04550 | Parathymosin OS=Rattus norvegicus OX=10116 GN=Ptms PE=1 SV=2 | 11.6 | 11 | 3 | 1 | 191688.3888 |  | 20375794.88 |
| F1M775 | Protein diaphanous homolog 1 OS=Rattus norvegicus OX=10116 GN=Diaph1 PE=1 SV=3 | 140.3 | 2 | 2 | 1 |  |  | 72425.375 |
| Q9JJM7 | Platelet glycoprotein Ib beta chain OS=Rattus norvegicus OX=10116 GN=Gp1bb PE=1 SV=1 | 22.2 | 5 | 2 | 1 | 64165.0275 |  | 136930.625 |
| Q9JHB5 | Translin-associated protein X OS=Rattus norvegicus OX=10116 GN=Tsnax PE=1 SV=1 | 33 | 7 | 1 | 1 | 47789.43511 |  | 74451.21875 |
| Q64057 | Alpha-aminoadipic semialdehyde dehydrogenase OS=Rattus norvegicus OX=10116 GN=Aldh7a1 PE=1 SV=2 | 58.7 | 2 | 4 | 1 |  |  | 77444.04688 |
| Q64602 | Kynurenine/alpha-aminoadipate aminotransferase. mitochondrial OS=Rattus norvegicus OX=10116 GN=Aadat PE=1 SV=1 | 47.8 | 2 | 1 | 1 |  |  | 867032.6875 |
| Q62908 | Cysteine and glycine-rich protein 2 OS=Rattus norvegicus OX=10116 GN=Csrp2 PE=1 SV=3 | 20.9 | 8 | 2 | 1 |  |  | 43536.36719 |
| Q64560 | Tripeptidyl-peptidase 2 OS=Rattus norvegicus OX=10116 GN=Tpp2 PE=2 SV=3 | 138.2 | 1 | 1 | 1 | 242135.627 |  | 126665.1172 |
| Q99N27 | Sorting nexin-1 OS=Rattus norvegicus OX=10116 GN=Snx1 PE=1 SV=1 | 59 | 2 | 1 | 1 |  |  | 88411.64844 |
| Q66HL2 | Src substrate cortactin OS=Rattus norvegicus OX=10116 GN=Cttn PE=1 SV=1 | 56.9 | 2 | 1 | 1 |  |  | 200205.3594 |
| P12785 | Fatty acid synthase OS=Rattus norvegicus OX=10116 GN=Fasn PE=1 SV=3 | 272.5 | 1 | 1 | 1 | 35059.94295 |  | 59460.4375 |
| P30919 | N(4)-(Beta-N-acetylglucosaminyl)-L-asparaginase OS=Rattus norvegicus OX=10116 GN=Aga PE=1 SV=2 | 37.1 | 5 | 1 | 1 |  |  | 24552.08008 |
| P14423 | Phospholipase A2. membrane associated OS=Rattus norvegicus OX=10116 GN=Pla2g2a PE=1 SV=2 | 16.3 | 12 | 1 | 1 | 29182.05919 |  | 68033.11719 |
| P14408 | Fumarate hydratase. mitochondrial OS=Rattus norvegicus OX=10116 GN=Fh PE=1 SV=2 | 54.4 | 2 | 1 | 1 |  |  | 856896.375 |
| P20673 | Argininosuccinate lyase OS=Rattus norvegicus OX=10116 GN=Asl PE=2 SV=1 | 51.5 | 4 | 2 | 1 |  |  | 44339.13672 |
| O35783 | Calumenin OS=Rattus norvegicus OX=10116 GN=Calu PE=1 SV=1 | 37 | 5 | 1 | 1 |  |  | 379885.0938 |
| Q63772 | Growth arrest-specific protein 6 OS=Rattus norvegicus OX=10116 GN=Gas6 PE=1 SV=1 | 74.6 | 3 | 1 | 1 | 69253.37047 |  | 41903.29297 |
| B4F795 | Choline transporter-like protein 2 OS=Rattus norvegicus OX=10116 GN=Slc44a2 PE=2 SV=1 | 79.8 | 2 | 1 | 1 |  |  | 72870.84375 |
| P57113 | Maleylacetoacetate isomerase OS=Rattus norvegicus OX=10116 GN=Gstz1 PE=1 SV=2 | 23.9 | 7 | 1 | 1 |  |  | 52835.52734 |
| F1M5N7 | Kinesin-like protein KIF21B OS=Rattus norvegicus OX=10116 GN=Kif21b PE=1 SV=2 | 182.5 | 2 | 1 | 1 |  |  | 1248879.375 |
| P14942 | Glutathione S-transferase alpha-4 OS=Rattus norvegicus OX=10116 GN=Gsta4 PE=1 SV=2 | 25.5 | 6 | 1 | 1 |  |  | 9252.53418 |
| P37377 | Alpha-synuclein OS=Rattus norvegicus OX=10116 GN=Snca PE=1 SV=1 | 14.5 | 31 | 1 | 1 |  |  | 542679.6875 |
| Q9R1E9 | CCN family member 2 OS=Rattus norvegicus OX=10116 GN=Ccn2 PE=2 SV=1 | 37.7 | 5 | 1 | 1 | 52191.83386 |  | 18802.32422 |
| P62994 | Growth factor receptor-bound protein 2 OS=Rattus norvegicus OX=10116 GN=Grb2 PE=1 SV=1 | 25.2 | 3 | 1 | 1 |  |  | 60484.9375 |
| O35820 | 2'-deoxynucleoside 5'-phosphate N-hydrolase 1 OS=Rattus norvegicus OX=10116 GN=Dnph1 PE=1 SV=1 | 17.8 | 6 | 2 | 1 | 1323775.199 |  | 1145417.625 |
| Q6MG97 | Butyrophilin-like protein 2 OS=Rattus norvegicus OX=10116 GN=Btnl2 PE=3 SV=1 | 50.7 | 4 | 1 | 1 |  |  | 1576117.375 |
| Q3SWT0 | Platelet endothelial cell adhesion molecule OS=Rattus norvegicus OX=10116 GN=Pecam1 PE=1 SV=1 | 76.1 | 2 | 1 | 1 |  |  | 30086.7207 |
| B2RZ78 | Vacuolar protein sorting-associated protein 29 OS=Rattus norvegicus OX=10116 GN=Vps29 PE=1 SV=2 | 20.5 | 5 | 1 | 1 |  |  | 108081.7656 |
| P35952 | Low-density lipoprotein receptor OS=Rattus norvegicus OX=10116 GN=Ldlr PE=1 SV=1 | 96.6 | 2 | 1 | 1 | 6933.648635 | 36816.24079 | 14280935 |
| P98106 | P-selectin OS=Rattus norvegicus OX=10116 GN=Selp PE=2 SV=1 | 83.5 | 1 | 1 | 1 |  |  | 118613.7969 |
| P53042 | Serine/threonine-protein phosphatase 5 OS=Rattus norvegicus OX=10116 GN=Ppp5c PE=1 SV=1 | 56.9 | 4 | 1 | 1 | 52860.97103 |  | 346764.8125 |
| Q4V7C7 | Actin-related protein 3 OS=Rattus norvegicus OX=10116 GN=Actr3 PE=1 SV=1 | 47.3 | 2 | 1 | 1 | 156871.7539 |  | 227037.9375 |
| O54858 | Carboxypeptidase Z OS=Rattus norvegicus OX=10116 GN=Cpz PE=2 SV=1 | 73 | 2 | 1 | 1 |  |  | 47650.14453 |
| P16303 | Carboxylesterase 1D OS=Rattus norvegicus OX=10116 GN=Ces1d PE=1 SV=2 | 62.1 | 1 | 1 | 1 |  |  | 1028548.438 |
| O35078 | D-amino-acid oxidase OS=Rattus norvegicus OX=10116 GN=Dao PE=2 SV=1 | 38.8 | 3 | 1 | 1 | 266197.3957 |  | 221958.875 |
| P10716 | C-type lectin domain family 4 member F OS=Rattus norvegicus OX=10116 GN=Clec4f PE=1 SV=1 | 61.1 | 12 | 12 | 5 | 616372.7905 | 166713.8448 | 97748.35156 |
| Q63468 | Phosphoribosyl pyrophosphate synthase-associated protein 1 OS=Rattus norvegicus OX=10116 GN=Prpsap1 PE=1 SV=1 | 39.4 | 34 | 15 | 8 | 424855.935 | 14632.00205 | 94713.27344 |
| P13601 | Aldehyde dehydrogenase. cytosolic 1 OS=Rattus norvegicus OX=10116 GN=Aldh1a7 PE=1 SV=2 | 54.5 | 10 | 6 | 1 |  |  |  |
| P00564 | Creatine kinase M-type OS=Rattus norvegicus OX=10116 GN=Ckm PE=1 SV=2 | 43 | 9 | 2 | 2 |  |  |  |
| P11980 | Pyruvate kinase PKM OS=Rattus norvegicus OX=10116 GN=Pkm PE=1 SV=3 | 57.8 | 7 | 7 | 2 |  |  |  |
| Q9QWJ9 | Neuropilin-1 OS=Rattus norvegicus OX=10116 GN=Nrp1 PE=1 SV=1 | 103 | 4 | 2 | 2 |  |  |  |
| P21139 | Alpha-mannosidase 2C1 OS=Rattus norvegicus OX=10116 GN=Man2c1 PE=1 SV=1 | 115.9 | 2 | 2 | 2 | 62509.80181 |  |  |
| Q68FW9 | COP9 signalosome complex subunit 3 OS=Rattus norvegicus OX=10116 GN=Cops3 PE=1 SV=1 | 47.8 | 9 | 2 | 2 |  |  |  |
| Q5RJQ4 | NAD-dependent protein deacetylase sirtuin-2 OS=Rattus norvegicus OX=10116 GN=Sirt2 PE=1 SV=1 | 39.3 | 7 | 2 | 1 |  |  |  |
| P35577 | Thyroxine-binding globulin OS=Rattus norvegicus OX=10116 GN=Serpina7 PE=1 SV=2 | 46.9 | 6 | 5 | 1 |  |  |  |
| Q9Z2Q4 | Methionine synthase OS=Rattus norvegicus OX=10116 GN=Mtr PE=1 SV=1 | 139.1 | 3 | 2 | 2 |  |  |  |
| Q9WVT0 | Adhesion G protein-coupled receptor F5 OS=Rattus norvegicus OX=10116 GN=Adgrf5 PE=1 SV=1 | 149.4 | 2 | 2 | 2 |  |  |  |
| O35814 | Stress-induced-phosphoprotein 1 OS=Rattus norvegicus OX=10116 GN=Stip1 PE=1 SV=1 | 62.5 | 4 | 2 | 2 |  |  |  |
| Q5HZV9 | Protein phosphatase 1 regulatory subunit 7 OS=Rattus norvegicus OX=10116 GN=Ppp1r7 PE=1 SV=1 | 41.3 | 5 | 1 | 1 |  |  |  |
| A0JPJ7 | Obg-like ATPase 1 OS=Rattus norvegicus OX=10116 GN=Ola1 PE=2 SV=1 | 44.5 | 4 | 1 | 1 | 61485.88912 |  |  |
| Q9Z1B2 | Glutathione S-transferase Mu 5 OS=Rattus norvegicus OX=10116 GN=Gstm5 PE=1 SV=3 | 26.6 | 7 | 1 | 1 |  |  |  |
| P61980 | Heterogeneous nuclear ribonucleoprotein K OS=Rattus norvegicus OX=10116 GN=Hnrnpk PE=1 SV=1 | 50.9 | 3 | 2 | 1 | 10842.57213 |  |  |
| Q64605 | Receptor-type tyrosine-protein phosphatase S OS=Rattus norvegicus OX=10116 GN=Ptprs PE=1 SV=2 | 211.8 | 1 | 3 | 1 |  |  |  |
| Q63081 | Protein disulfide-isomerase A6 OS=Rattus norvegicus OX=10116 GN=Pdia6 PE=1 SV=2 | 48.1 | 5 | 2 | 1 |  |  |  |
| P48679 | Prelamin-A/C OS=Rattus norvegicus OX=10116 GN=Lmna PE=1 SV=1 | 74.3 | 2 | 1 | 1 | 19914.09313 |  |  |
| Q62956 | Receptor tyrosine-protein kinase erbB-4 OS=Rattus norvegicus OX=10116 GN=Erbb4 PE=1 SV=3 | 146.9 | 2 | 1 | 1 |  |  |  |
| P38659 | Protein disulfide-isomerase A4 OS=Rattus norvegicus OX=10116 GN=Pdia4 PE=1 SV=2 | 72.7 | 2 | 1 | 1 |  |  |  |
| P08009 | Glutathione S-transferase Yb-3 OS=Rattus norvegicus OX=10116 GN=Gstm3 PE=1 SV=2 | 25.7 | 6 | 2 | 1 |  |  |  |
| Q5XID1 | Anamorsin OS=Rattus norvegicus OX=10116 GN=Ciapin1 PE=1 SV=1 | 33 | 8 | 1 | 1 |  |  |  |
| P0C6B8 | Sushi. von Willebrand factor type A. EGF and pentraxin domain-containing protein 1 OS=Rattus norvegicus OX=10116 GN=Svep1 PE=1 SV=1 | 387.1 | 1 | 4 | 2 | 1210176.713 | 1369679.458 | 112554.5234 |
| P61206 | ADP-ribosylation factor 3 OS=Rattus norvegicus OX=10116 GN=Arf3 PE=2 SV=2 | 20.6 | 6 | 1 | 1 |  |  |  |
| Q5HZE4 | Methylthioribose-1-phosphate isomerase OS=Rattus norvegicus OX=10116 GN=Mri1 PE=1 SV=1 | 39.6 | 4 | 1 | 1 |  |  |  |
| Q5BJK8 | Golgi integral membrane protein 4 OS=Rattus norvegicus OX=10116 GN=Golim4 PE=1 SV=2 | 76.6 | 3 | 2 | 1 |  |  |  |
| P62898 | Cytochrome c. somatic OS=Rattus norvegicus OX=10116 GN=Cycs PE=1 SV=2 | 11.6 | 10 | 2 | 1 |  |  |  |
| P07896 | Peroxisomal bifunctional enzyme OS=Rattus norvegicus OX=10116 GN=Ehhadh PE=1 SV=2 | 78.6 | 2 | 1 | 1 |  |  |  |
| Q68FT9 | Selenocysteine lyase OS=Rattus norvegicus OX=10116 GN=Scly PE=1 SV=1 | 47.2 | 3 | 1 | 1 |  |  |  |
| P62718 | 60S ribosomal protein L18a OS=Rattus norvegicus OX=10116 GN=Rpl18a PE=2 SV=1 | 20.7 | 7 | 1 | 1 |  |  |  |
| P31000 | Vimentin OS=Rattus norvegicus OX=10116 GN=Vim PE=1 SV=2 | 53.7 | 2 | 1 | 1 |  |  |  |
| P36972 | Adenine phosphoribosyltransferase OS=Rattus norvegicus OX=10116 GN=Aprt PE=1 SV=1 | 19.5 | 11 | 1 | 1 |  |  |  |
| P62963 | Profilin-1 OS=Rattus norvegicus OX=10116 GN=Pfn1 PE=1 SV=2 | 14.9 | 10 | 2 | 1 |  |  |  |
| P49134 | Integrin beta-1 OS=Rattus norvegicus OX=10116 GN=Itgb1 PE=2 SV=1 | 88.4 | 2 | 1 | 1 |  |  |  |
| P32362 | Uroporphyrinogen decarboxylase (Fragment) OS=Rattus norvegicus OX=10116 GN=Urod PE=1 SV=1 | 40.4 | 3 | 1 | 1 |  |  |  |
| O35987 | NSFL1 cofactor p47 OS=Rattus norvegicus OX=10116 GN=Nsfl1c PE=1 SV=1 | 40.7 | 4 | 1 | 1 |  |  |  |
| Q7TPA5 | Serpin A11 OS=Rattus norvegicus OX=10116 GN=Serpina11 PE=2 SV=2 | 47 | 8 | 2 | 2 | 1504369 | 207878.367 | 489861.75 |
| O88775 | Embigin OS=Rattus norvegicus OX=10116 GN=Emb PE=1 SV=1 | 37 | 3 | 1 | 1 |  |  |  |
| Q99PS8 | Histidine-rich glycoprotein OS=Rattus norvegicus OX=10116 GN=Hrg PE=1 SV=1 | 59 | 2 | 1 | 1 |  |  |  |
| Q05695 | Neural cell adhesion molecule L1 OS=Rattus norvegicus OX=10116 GN=L1cam PE=1 SV=3 | 140.8 | 1 | 2 | 1 |  |  |  |
| B0BN93 | 26S proteasome non-ATPase regulatory subunit 13 OS=Rattus norvegicus OX=10116 GN=Psmd13 PE=1 SV=1 | 42.8 | 3 | 1 | 1 | 143045.1536 |  |  |
| Q4W8E7 | Adherens junction-associated protein 1 OS=Rattus norvegicus OX=10116 GN=Ajap1 PE=2 SV=1 | 44.7 | 10 | 1 | 1 |  |  |  |
| B4F777 | High mobility group nucleosome-binding domain-containing protein 5 OS=Rattus norvegicus OX=10116 GN=Hmgn5 PE=2 SV=1 | 48.6 | 3 | 1 | 1 |  |  |  |
| P50398 | Rab GDP dissociation inhibitor alpha OS=Rattus norvegicus OX=10116 GN=Gdi1 PE=1 SV=1 | 50.5 | 4 | 1 | 1 |  |  |  |
| P08081 | Clathrin light chain A OS=Rattus norvegicus OX=10116 GN=Clta PE=1 SV=1 | 27 | 3 | 1 | 1 |  |  |  |
| Q4KLH4 | Paraspeckle component 1 OS=Rattus norvegicus OX=10116 GN=Pspc1 PE=1 SV=1 | 58.7 | 6 | 1 | 1 |  |  |  |
| Q62600 | Nitric oxide synthase. endothelial OS=Rattus norvegicus OX=10116 GN=Nos3 PE=1 SV=4 | 133.2 | 3 | 1 | 1 |  |  |  |
| Q63060 | Glycerol kinase OS=Rattus norvegicus OX=10116 GN=Gk PE=2 SV=1 | 57.4 | 2 | 1 | 1 |  |  |  |
| P48966 | M-phase inducer phosphatase 2 OS=Rattus norvegicus OX=10116 GN=Cdc25b PE=1 SV=1 | 64.2 | 3 | 1 | 1 |  |  |  |
| Q9Z1L0 | Phosphatidylinositol 4.5-bisphosphate 3-kinase catalytic subunit beta isoform OS=Rattus norvegicus OX=10116 GN=Pik3cb PE=2 SV=1 | 122.5 | 1 | 2 | 1 | 1736193.307 |  |  |

Supplementary apenix 2.

| Proteins identified in renal eluates from rats using the Swiss-prot mouse database | | | | | | | | | |
| --- | --- | --- | --- | --- | --- | --- | --- | --- | --- |
| Accession | Description | MW [kDa] | Coverage [%] | # PSMs | # Peptides quantified | | Abundances (Normalized): HO: Sample | Abundances (Normalized): NS: Sample | Abundances (Normalized): HS: Sample |
| P15508 | Spectrin beta chain. erythrocytic OS=Mus musculus OX=10090 GN=Sptb PE=1 SV=4 | 245.1 | 39 | 348 | 64 | 26994878 | | 14514821 | 93037783 |
| P01027 | Complement C3 OS=Mus musculus OX=10090 GN=C3 PE=1 SV=3 | 186.4 | 32 | 1738 | 46 | 3604080799 | | 3793866030 | 3187540464 |
| P08032 | Spectrin alpha chain. erythrocytic 1 OS=Mus musculus OX=10090 GN=Spta1 PE=1 SV=3 | 279.7 | 21 | 219 | 44 | 30523741 | | 17319764 | 89771508 |
| P26039 | Talin-1 OS=Mus musculus OX=10090 GN=Tln1 PE=1 SV=2 | 269.7 | 30 | 143 | 45 | 5150915 | | 390068 | 25536364 |
| P11276 | Fibronectin OS=Mus musculus OX=10090 GN=Fn1 PE=1 SV=4 | 272.4 | 24 | 365 | 40 | 23908475 | | 89577741 | 104637297 |
| P57780 | Alpha-actinin-4 OS=Mus musculus OX=10090 GN=Actn4 PE=1 SV=1 | 104.9 | 52 | 183 | 23 | 3884323 | | 247197 | 28908777 |
| Q7TPR4 | Alpha-actinin-1 OS=Mus musculus OX=10090 GN=Actn1 PE=1 SV=1 | 103 | 49 | 172 | 19 | 2457734 | | 3489722 | 21754950 |
| P07724 | Serum albumin OS=Mus musculus OX=10090 GN=Alb PE=1 SV=3 | 68.6 | 42 | 6558 | 27 | 26049653015 | | 29519890148 | 20072248232 |
| P60710 | Actin. cytoplasmic 1 OS=Mus musculus OX=10090 GN=Actb PE=1 SV=1 | 41.7 | 68 | 645 | 9 | 237276751 | | 133543038 | 485218397 |
| Q64487 | Receptor-type tyrosine-protein phosphatase delta OS=Mus musculus OX=10090 GN=Ptprd PE=1 SV=3 | 214.3 | 31 | 93 | 36 | 199055 | | 325576 | 57358566 |
| P28666 | Murinoglobulin-2 OS=Mus musculus OX=10090 GN=Mug2 PE=1 SV=2 | 162.3 | 12 | 2353 | 3 | 1170143638 | | 1564395646 | 1084932094 |
| P63017 | Heat shock cognate 71 kDa protein OS=Mus musculus OX=10090 GN=Hspa8 PE=1 SV=1 | 70.8 | 45 | 294 | 22 | 286901851 | | 49655630 | 291661727 |
| Q91Y97 | Fructose-bisphosphate aldolase B OS=Mus musculus OX=10090 GN=Aldob PE=1 SV=3 | 39.5 | 59 | 237 | 12 | 159845198 | | 164378380 | 1077756088 |
| P19221 | Prothrombin OS=Mus musculus OX=10090 GN=F2 PE=1 SV=1 | 70.2 | 31 | 247 | 17 | 99853051 | | 185547256 | 315626113 |
| P28665 | Murinoglobulin-1 OS=Mus musculus OX=10090 GN=Mug1 PE=1 SV=3 | 165.2 | 12 | 1907 | 3 | 158853257 | | 231812266 | 177372007 |
| P05064 | Fructose-bisphosphate aldolase A OS=Mus musculus OX=10090 GN=Aldoa PE=1 SV=2 | 39.3 | 62 | 144 | 14 | 119794614 | | 22702807 | 115816330 |
| Q01853 | Transitional endoplasmic reticulum ATPase OS=Mus musculus OX=10090 GN=Vcp PE=1 SV=4 | 89.3 | 45 | 107 | 26 | 12990237 | | 4321300 | 16439754 |
| Q02053 | Ubiquitin-like modifier-activating enzyme 1 OS=Mus musculus OX=10090 GN=Uba1 PE=1 SV=1 | 117.7 | 37 | 86 | 21 | 29732005 | | 1285625 | 46697367 |
| Q8R016 | Bleomycin hydrolase OS=Mus musculus OX=10090 GN=Blmh PE=1 SV=1 | 52.5 | 43 | 109 | 15 | 57006245 | | 22449276 | 31084533 |
| P01029 | Complement C4-B OS=Mus musculus OX=10090 GN=C4b PE=1 SV=3 | 192.8 | 11 | 227 | 17 | 122120502 | | 88779983 | 230931849 |
| P32261 | Antithrombin-III OS=Mus musculus OX=10090 GN=Serpinc1 PE=1 SV=1 | 52 | 27 | 384 | 12 | 997399392 | | 784865311 | 1490227486 |
| P24270 | Catalase OS=Mus musculus OX=10090 GN=Cat PE=1 SV=4 | 59.8 | 31 | 243 | 14 | 583141677 | | 68382358 | 363509257 |
| Q05793 | Basement membrane-specific heparan sulfate proteoglycan core protein OS=Mus musculus OX=10090 GN=Hspg2 PE=1 SV=1 | 398 | 11 | 76 | 26 | 337796644 | | 19946442 | 20731486 |
| P58252 | Elongation factor 2 OS=Mus musculus OX=10090 GN=Eef2 PE=1 SV=2 | 95.3 | 38 | 70 | 21 | 665844 | | 154351 | 33699288 |
| Q91X72 | Hemopexin OS=Mus musculus OX=10090 GN=Hpx PE=1 SV=2 | 51.3 | 30 | 939 | 16 | 2146238462 | | 3197000987 | 2468862899 |
| P68368 | Tubulin alpha-4A chain OS=Mus musculus OX=10090 GN=Tuba4a PE=1 SV=1 | 49.9 | 46 | 90 | 4 | 1295897 | | 866513 | 6292391 |
| P05213 | Tubulin alpha-1B chain OS=Mus musculus OX=10090 GN=Tuba1b PE=1 SV=2 | 50.1 | 45 | 101 | 4 | 2228205 | | 549403 | 6241226 |
| Q61147 | Ceruloplasmin OS=Mus musculus OX=10090 GN=Cp PE=1 SV=2 | 121.1 | 14 | 353 | 14 | 598033247 | | 541421567 | 428464747 |
| P99024 | Tubulin beta-5 chain OS=Mus musculus OX=10090 GN=Tubb5 PE=1 SV=1 | 49.6 | 55 | 78 | 2 |  | |  | 243487 |
| Q921I1 | Serotransferrin OS=Mus musculus OX=10090 GN=Tf PE=1 SV=1 | 76.7 | 21 | 243 | 17 | 2099014623 | | 2330367756 | 2409965790 |
| Q61703 | Inter-alpha-trypsin inhibitor heavy chain H2 OS=Mus musculus OX=10090 GN=Itih2 PE=1 SV=1 | 105.9 | 16 | 216 | 10 | 89071151 | | 122786833 | 138127906 |
| P68372 | Tubulin beta-4B chain OS=Mus musculus OX=10090 GN=Tubb4b PE=1 SV=1 | 49.8 | 58 | 81 | 1 | 101498 | |  | 1888467 |
| Q08879 | Fibulin-1 OS=Mus musculus OX=10090 GN=Fbln1 PE=1 SV=2 | 78 | 28 | 66 | 13 | 15525984 | | 9478711 | 8150438 |
| Q9DBD0 | Inhibitor of carbonic anhydrase OS=Mus musculus OX=10090 GN=Ica PE=1 SV=1 | 76.7 | 18 | 107 | 10 | 106870424 | | 246488824 | 181345846 |
| Q9CWJ9 | Bifunctional purine biosynthesis protein PURH OS=Mus musculus OX=10090 GN=Atic PE=1 SV=2 | 64.2 | 37 | 62 | 16 | 73159773 | | 6500497 | 50564541 |
| P63101 | 14-3-3 protein zeta/delta OS=Mus musculus OX=10090 GN=Ywhaz PE=1 SV=1 | 27.8 | 55 | 106 | 8 | 28970559 | | 13421257 | 146574704 |
| Q8K0E8 | Fibrinogen beta chain OS=Mus musculus OX=10090 GN=Fgb PE=1 SV=1 | 54.7 | 32 | 102 | 13 | 1694092 | | 34066341 | 113653401 |
| Q61704 | Inter-alpha-trypsin inhibitor heavy chain H3 OS=Mus musculus OX=10090 GN=Itih3 PE=1 SV=3 | 99.3 | 18 | 191 | 11 | 126565599 | | 195934851 | 164504869 |
| P97494 | Glutamate--cysteine ligase catalytic subunit OS=Mus musculus OX=10090 GN=Gclc PE=1 SV=4 | 72.5 | 37 | 67 | 18 | 37454354 | | 3199817 | 64270893 |
| P13020 | Gelsolin OS=Mus musculus OX=10090 GN=Gsn PE=1 SV=3 | 85.9 | 22 | 199 | 9 | 70651813 | | 66942531 | 123925396 |
| Q7TMM9 | Tubulin beta-2A chain OS=Mus musculus OX=10090 GN=Tubb2a PE=1 SV=1 | 49.9 | 43 | 67 | 2 | 89168 | | 12814 | 502961 |
| Q8R146 | Acylamino-acid-releasing enzyme OS=Mus musculus OX=10090 GN=Apeh PE=1 SV=3 | 81.5 | 31 | 97 | 14 | 39053621 | | 4157057 | 24881734 |
| Q61838 | Pregnancy zone protein OS=Mus musculus OX=10090 GN=Pzp PE=1 SV=3 | 165.7 | 9 | 696 | 13 | 622136201 | | 500095326 | 783416850 |
| Q9QXD6 | Fructose-1.6-bisphosphatase 1 OS=Mus musculus OX=10090 GN=Fbp1 PE=1 SV=3 | 36.9 | 33 | 36 | 9 | 45544 | |  | 33716475 |
| P56399 | Ubiquitin carboxyl-terminal hydrolase 5 OS=Mus musculus OX=10090 GN=Usp5 PE=1 SV=1 | 95.8 | 36 | 64 | 18 | 8585062 | | 595493 | 11220032 |
| P35441 | Thrombospondin-1 OS=Mus musculus OX=10090 GN=Thbs1 PE=1 SV=1 | 129.6 | 17 | 103 | 15 | 5424925 | | 4123910 | 11830379 |
| P02088 | Hemoglobin subunit beta-1 OS=Mus musculus OX=10090 GN=Hbb-b1 PE=1 SV=2 | 15.8 | 33 | 372 | 2 | 334518462 | | 76079466 | 239741187 |
| O88844 | Isocitrate dehydrogenase [NADP] cytoplasmic OS=Mus musculus OX=10090 GN=Idh1 PE=1 SV=2 | 46.6 | 38 | 56 | 14 | 791740 | | 1479377 | 136658202 |
| P62259 | 14-3-3 protein epsilon OS=Mus musculus OX=10090 GN=Ywhae PE=1 SV=1 | 29.2 | 49 | 94 | 11 | 25689956 | | 4720420 | 89221063 |
| Q8BTM8 | Filamin-A OS=Mus musculus OX=10090 GN=Flna PE=1 SV=5 | 281 | 12 | 62 | 19 | 2492846 | | 248435 | 21049031 |
| P28271 | Cytoplasmic aconitate hydratase OS=Mus musculus OX=10090 GN=Aco1 PE=1 SV=3 | 98.1 | 28 | 45 | 15 | 2433234 | | 1145353 | 37223151 |
| Q01279 | Epidermal growth factor receptor OS=Mus musculus OX=10090 GN=Egfr PE=1 SV=1 | 134.8 | 13 | 58 | 12 | 14790294 | | 19180521 | 13572286 |
| Q61316 | Heat shock 70 kDa protein 4 OS=Mus musculus OX=10090 GN=Hspa4 PE=1 SV=1 | 94.1 | 25 | 50 | 14 | 9048043 | | 426424 | 15827516 |
| Q64727 | Vinculin OS=Mus musculus OX=10090 GN=Vcl PE=1 SV=4 | 116.6 | 23 | 48 | 16 | 6343109 | | 1362227 | 27264975 |
| O09061 | Proteasome subunit beta type-1 OS=Mus musculus OX=10090 GN=Psmb1 PE=1 SV=1 | 26.4 | 39 | 54 | 7 | 9901444 | | 517155 | 7364722 |
| P40142 | Transketolase OS=Mus musculus OX=10090 GN=Tkt PE=1 SV=1 | 67.6 | 30 | 53 | 10 | 16090880 | | 899934 | 35992654 |
| P02089 | Hemoglobin subunit beta-2 OS=Mus musculus OX=10090 GN=Hbb-b2 PE=1 SV=2 | 15.9 | 45 | 377 | 4 | 509342285 | | 137362543 | 370533972 |
| Q8BH35 | Complement component C8 beta chain OS=Mus musculus OX=10090 GN=C8b PE=1 SV=1 | 66.2 | 19 | 61 | 9 | 13882577 | | 14560392 | 17240669 |
| Q9D6F9 | Tubulin beta-4A chain OS=Mus musculus OX=10090 GN=Tubb4a PE=1 SV=3 | 49.6 | 52 | 67 | 1 |  | |  | 310836 |
| Q8VCM7 | Fibrinogen gamma chain OS=Mus musculus OX=10090 GN=Fgg PE=1 SV=1 | 49.4 | 23 | 164 | 7 | 2372585 | | 59594012 | 267771792 |
| Q9R111 | Guanine deaminase OS=Mus musculus OX=10090 GN=Gda PE=1 SV=1 | 51 | 35 | 66 | 12 | 77086876 | | 3139664 | 17623898 |
| Q8BFZ3 | Beta-actin-like protein 2 OS=Mus musculus OX=10090 GN=Actbl2 PE=1 SV=1 | 42 | 27 | 179 | 2 | 7444824 | | 2147323 | 16933887 |
| O88958 | Glucosamine-6-phosphate isomerase 1 OS=Mus musculus OX=10090 GN=Gnpda1 PE=1 SV=3 | 32.5 | 39 | 31 | 7 | 8913153 | | 4560014 | 10611417 |
| P16858 | Glyceraldehyde-3-phosphate dehydrogenase OS=Mus musculus OX=10090 GN=Gapdh PE=1 SV=2 | 35.8 | 41 | 89 | 10 | 8670547 | | 2811508 | 37003434 |
| Q9WU60 | Attractin OS=Mus musculus OX=10090 GN=Atrn PE=1 SV=3 | 158 | 12 | 35 | 12 | 8625122 | | 11449542 | 7726179 |
| P68134 | Actin. alpha skeletal muscle OS=Mus musculus OX=10090 GN=Acta1 PE=1 SV=1 | 42 | 38 | 189 | 3 | 99139175 | | 31689094 | 167092368 |
| P14152 | Malate dehydrogenase. cytoplasmic OS=Mus musculus OX=10090 GN=Mdh1 PE=1 SV=3 | 36.5 | 41 | 40 | 11 | 52384291 | | 7623488 | 439478933 |
| Q9JMH6 | Thioredoxin reductase 1. cytoplasmic OS=Mus musculus OX=10090 GN=Txnrd1 PE=1 SV=3 | 67 | 24 | 38 | 10 | 826923 | | 37784 | 24428641 |
| P17751 | Triosephosphate isomerase OS=Mus musculus OX=10090 GN=Tpi1 PE=1 SV=4 | 32.2 | 39 | 53 | 8 | 17112599 | | 1790010 | 161205171 |
| Q9Z2U1 | Proteasome subunit alpha type-5 OS=Mus musculus OX=10090 GN=Psma5 PE=1 SV=1 | 26.4 | 39 | 41 | 6 | 9308163 | | 358460 | 8759358 |
| P46412 | Glutathione peroxidase 3 OS=Mus musculus OX=10090 GN=Gpx3 PE=1 SV=2 | 25.4 | 43 | 679 | 8 | 468696714 | | 137022566 | 3221038799 |
| P20029 | Endoplasmic reticulum chaperone BiP OS=Mus musculus OX=10090 GN=Hspa5 PE=1 SV=3 | 72.4 | 20 | 50 | 10 | 5508964 | | 286003 | 16660917 |
| Q01768 | Nucleoside diphosphate kinase B OS=Mus musculus OX=10090 GN=Nme2 PE=1 SV=1 | 17.4 | 59 | 113 | 3 | 14948134 | | 1090906 | 69946572 |
| P16460 | Argininosuccinate synthase OS=Mus musculus OX=10090 GN=Ass1 PE=1 SV=1 | 46.6 | 39 | 54 | 12 | 2030303 | | 1095431 | 15573262 |
| Q68FD5 | Clathrin heavy chain 1 OS=Mus musculus OX=10090 GN=Cltc PE=1 SV=3 | 191.4 | 14 | 28 | 18 | 1408528 | | 22275 | 735009 |
| A2AQ07 | Tubulin beta-1 chain OS=Mus musculus OX=10090 GN=Tubb1 PE=1 SV=1 | 50.4 | 35 | 45 | 9 | 751054 | | 336072 | 1528061 |
| Q9DBJ1 | Phosphoglycerate mutase 1 OS=Mus musculus OX=10090 GN=Pgam1 PE=1 SV=3 | 28.8 | 49 | 20 | 7 | 53663 | | 456914 | 81378531 |
| P16125 | L-lactate dehydrogenase B chain OS=Mus musculus OX=10090 GN=Ldhb PE=1 SV=2 | 36.5 | 41 | 38 | 10 | 86154 | | 102120 | 18757288 |
| P70389 | Insulin-like growth factor-binding protein complex acid labile subunit OS=Mus musculus OX=10090 GN=Igfals PE=1 SV=1 | 66.9 | 16 | 74 | 7 | 257742799 | | 229581992 | 171648386 |
| Q11136 | Xaa-Pro dipeptidase OS=Mus musculus OX=10090 GN=Pepd PE=1 SV=3 | 55 | 21 | 35 | 8 | 6825681 | | 2163905 | 94099433 |
| P10518 | Delta-aminolevulinic acid dehydratase OS=Mus musculus OX=10090 GN=Alad PE=1 SV=1 | 36 | 31 | 57 | 8 | 14241171 | | 4156274 | 19802940 |
| Q9JIL4 | Na(+)/H(+) exchange regulatory cofactor NHE-RF3 OS=Mus musculus OX=10090 GN=Pdzk1 PE=1 SV=1 | 56.5 | 22 | 19 | 9 | 118835 | | 93895 | 30052426 |
| P50247 | Adenosylhomocysteinase OS=Mus musculus OX=10090 GN=Ahcy PE=1 SV=3 | 47.7 | 38 | 27 | 13 | 11810944 | | 493276 | 11191679 |
| P21614 | Vitamin D-binding protein OS=Mus musculus OX=10090 GN=Gc PE=1 SV=2 | 53.6 | 12 | 136 | 7 | 332721690 | | 341084089 | 375491187 |
| P17182 | Alpha-enolase OS=Mus musculus OX=10090 GN=Eno1 PE=1 SV=3 | 47.1 | 34 | 35 | 7 | 520446 | | 143442 | 5844607 |
| P00920 | Carbonic anhydrase 2 OS=Mus musculus OX=10090 GN=Ca2 PE=1 SV=4 | 29 | 23 | 80 | 5 | 230822743 | | 159146995 | 146116766 |
| P04186 | Complement factor B OS=Mus musculus OX=10090 GN=Cfb PE=1 SV=2 | 85 | 16 | 104 | 9 | 103266996 | | 121164303 | 126211056 |
| P06684 | Complement C5 OS=Mus musculus OX=10090 GN=C5 PE=1 SV=2 | 188.8 | 8 | 76 | 10 | 52979553 | | 45809956 | 59784001 |
| Q61702 | Inter-alpha-trypsin inhibitor heavy chain H1 OS=Mus musculus OX=10090 GN=Itih1 PE=1 SV=2 | 101 | 10 | 113 | 8 | 33656659 | | 46901901 | 44211162 |
| P42703 | Leukemia inhibitory factor receptor OS=Mus musculus OX=10090 GN=Lifr PE=1 SV=1 | 122.5 | 9 | 65 | 8 | 23878710 | | 41984034 | 31930977 |
| P16546 | Spectrin alpha chain. non-erythrocytic 1 OS=Mus musculus OX=10090 GN=Sptan1 PE=1 SV=4 | 284.4 | 8 | 25 | 15 | 77047 | |  | 1384501 |
| O09173 | Homogentisate 1.2-dioxygenase OS=Mus musculus OX=10090 GN=Hgd PE=1 SV=2 | 49.9 | 37 | 31 | 10 | 150053 | | 253514 | 5747362 |
| O70435 | Proteasome subunit alpha type-3 OS=Mus musculus OX=10090 GN=Psma3 PE=1 SV=3 | 28.4 | 27 | 44 | 6 | 12457132 | | 227428 | 8707516 |
| P06745 | Glucose-6-phosphate isomerase OS=Mus musculus OX=10090 GN=Gpi PE=1 SV=4 | 62.7 | 13 | 24 | 5 | 16191507 | | 168351 | 74179843 |
| P14211 | Calreticulin OS=Mus musculus OX=10090 GN=Calr PE=1 SV=1 | 48 | 30 | 32 | 8 | 4255055 | | 126055 | 12829115 |
| A2A8L5 | Receptor-type tyrosine-protein phosphatase F OS=Mus musculus OX=10090 GN=Ptprf PE=1 SV=1 | 211.4 | 9 | 24 | 11 | 8792 | | 13780 | 1888058 |
| Q6ZQ38 | Cullin-associated NEDD8-dissociated protein 1 OS=Mus musculus OX=10090 GN=Cand1 PE=1 SV=2 | 136.2 | 12 | 22 | 11 | 213462 | | 813339 | 3093265 |
| P47791 | Glutathione reductase. mitochondrial OS=Mus musculus OX=10090 GN=Gsr PE=1 SV=3 | 53.6 | 27 | 15 | 8 | 599827 | | 484942 | 49396086 |
| P21107 | Tropomyosin alpha-3 chain OS=Mus musculus OX=10090 GN=Tpm3 PE=1 SV=3 | 33 | 24 | 41 | 5 | 13548110 | | 1035046 | 38534075 |
| P68254 | 14-3-3 protein theta OS=Mus musculus OX=10090 GN=Ywhaq PE=1 SV=1 | 27.8 | 42 | 50 | 5 | 1747543 | | 98784 | 26482319 |
| Q00519 | Xanthine dehydrogenase/oxidase OS=Mus musculus OX=10090 GN=Xdh PE=1 SV=5 | 146.5 | 10 | 43 | 10 | 4493521 | | 3279919 | 5657224 |
| P47199 | Quinone oxidoreductase OS=Mus musculus OX=10090 GN=Cryz PE=1 SV=1 | 35.2 | 28 | 14 | 6 |  | | 421714 | 34728820 |
| P08226 | Apolipoprotein E OS=Mus musculus OX=10090 GN=Apoe PE=1 SV=2 | 35.8 | 18 | 107 | 7 | 371750017 | | 3319897 | 179241995 |
| P58774 | Tropomyosin beta chain OS=Mus musculus OX=10090 GN=Tpm2 PE=1 SV=1 | 32.8 | 21 | 49 | 4 | 174993990 | | 11662808 | 27964315 |
| Q3V0K9 | Plastin-1 OS=Mus musculus OX=10090 GN=Pls1 PE=1 SV=1 | 70.4 | 22 | 21 | 9 | 251203 | | 156844 | 15476275 |
| Q8R3P0 | Aspartoacylase OS=Mus musculus OX=10090 GN=Aspa PE=1 SV=2 | 35.3 | 35 | 29 | 8 | 1344929 | | 366027 | 31781680 |
| Q62433 | Protein NDRG1 OS=Mus musculus OX=10090 GN=Ndrg1 PE=1 SV=1 | 43 | 25 | 43 | 6 | 485124 | | 32928 | 28858992 |
| Q9EQH3 | Vacuolar protein sorting-associated protein 35 OS=Mus musculus OX=10090 GN=Vps35 PE=1 SV=1 | 91.7 | 13 | 13 | 8 | 207295 | |  | 1753788 |
| Q9QUM9 | Proteasome subunit alpha type-6 OS=Mus musculus OX=10090 GN=Psma6 PE=1 SV=1 | 27.4 | 39 | 35 | 8 | 12710826 | | 1633388 | 12940786 |
| O89020 | Afamin OS=Mus musculus OX=10090 GN=Afm PE=1 SV=2 | 69.3 | 11 | 97 | 9 | 236384415 | | 276943197 | 217633182 |
| P51885 | Lumican OS=Mus musculus OX=10090 GN=Lum PE=1 SV=2 | 38.2 | 18 | 110 | 4 | 42791305 | | 33742022 | 197859885 |
| E9PV24 | Fibrinogen alpha chain OS=Mus musculus OX=10090 GN=Fga PE=1 SV=1 | 87.4 | 9 | 67 | 7 | 2393686 | | 22485946 | 173964209 |
| Q06890 | Clusterin OS=Mus musculus OX=10090 GN=Clu PE=1 SV=1 | 51.6 | 17 | 69 | 8 | 5785458 | | 7430463 | 10674558 |
| Q9R1P4 | Proteasome subunit alpha type-1 OS=Mus musculus OX=10090 GN=Psma1 PE=1 SV=1 | 29.5 | 34 | 33 | 8 | 7998698 | | 644497 | 10238922 |
| Q80YX1 | Tenascin OS=Mus musculus OX=10090 GN=Tnc PE=1 SV=1 | 231.7 | 9 | 26 | 13 | 1673958 | | 563696 | 3147017 |
| P58771 | Tropomyosin alpha-1 chain OS=Mus musculus OX=10090 GN=Tpm1 PE=1 SV=1 | 32.7 | 17 | 38 | 3 | 4674374 | | 24635 | 25867758 |
| Q9JJN5 | Carboxypeptidase N catalytic chain OS=Mus musculus OX=10090 GN=Cpn1 PE=1 SV=1 | 51.8 | 12 | 50 | 4 | 12512841 | | 16388748 | 20927615 |
| P97798 | Neogenin OS=Mus musculus OX=10090 GN=Neo1 PE=1 SV=1 | 163.1 | 9 | 24 | 9 | 840729 | | 52236 | 4662510 |
| P61982 | 14-3-3 protein gamma OS=Mus musculus OX=10090 GN=Ywhag PE=1 SV=2 | 28.3 | 39 | 54 | 4 | 894749 | | 71506 | 8399273 |
| P49722 | Proteasome subunit alpha type-2 OS=Mus musculus OX=10090 GN=Psma2 PE=1 SV=3 | 25.9 | 38 | 28 | 6 | 2658021 | | 832582 | 2085575 |
| Q8BHN3 | Neutral alpha-glucosidase AB OS=Mus musculus OX=10090 GN=Ganab PE=1 SV=1 | 106.8 | 13 | 34 | 9 | 1145124 | | 937718 | 2017715 |
| P99026 | Proteasome subunit beta type-4 OS=Mus musculus OX=10090 GN=Psmb4 PE=1 SV=1 | 29.1 | 22 | 15 | 3 | 1573776 | | 59462 | 972252 |
| Q6GQT1 | Alpha-2-macroglobulin-P OS=Mus musculus OX=10090 GN=A2m PE=2 SV=2 | 164.2 | 8 | 124 | 8 | 200933655 | | 230598256 | 122765656 |
| P15532 | Nucleoside diphosphate kinase A OS=Mus musculus OX=10090 GN=Nme1 PE=1 SV=1 | 17.2 | 49 | 77 | 2 | 11427879 | | 2022875 | 11314749 |
| Q9CQV8 | 14-3-3 protein beta/alpha OS=Mus musculus OX=10090 GN=Ywhab PE=1 SV=3 | 28.1 | 35 | 50 | 2 | 1016520 | | 35232 | 8099273 |
| P06801 | NADP-dependent malic enzyme OS=Mus musculus OX=10090 GN=Me1 PE=1 SV=2 | 63.9 | 25 | 14 | 9 | 122329 | | 282282 | 1212943 |
| P20918 | Plasminogen OS=Mus musculus OX=10090 GN=Plg PE=1 SV=3 | 90.7 | 13 | 43 | 8 | 62580334 | | 77209415 | 48527919 |
| Q91VH6 | Protein MEMO1 OS=Mus musculus OX=10090 GN=Memo1 PE=1 SV=1 | 33.7 | 35 | 24 | 7 | 339195 | | 27226 | 1768854 |
| Q9QYB1 | Chloride intracellular channel protein 4 OS=Mus musculus OX=10090 GN=Clic4 PE=1 SV=3 | 28.7 | 57 | 29 | 8 | 254042 | | 142403 | 8430549 |
| P49182 | Heparin cofactor 2 OS=Mus musculus OX=10090 GN=Serpind1 PE=1 SV=1 | 54.5 | 19 | 66 | 6 | 34454139 | | 46768922 | 58478868 |
| Q62261 | Spectrin beta chain. non-erythrocytic 1 OS=Mus musculus OX=10090 GN=Sptbn1 PE=1 SV=2 | 274.1 | 2 | 35 | 1 |  | |  |  |
| P16294 | Coagulation factor IX OS=Mus musculus OX=10090 GN=F9 PE=2 SV=3 | 52.9 | 23 | 43 | 6 | 47917222 | | 12489813 | 69387946 |
| P49945 | Ferritin light chain 2 OS=Mus musculus OX=10090 GN=Ftl2 PE=3 SV=2 | 20.8 | 33 | 37 | 4 | 9307645 | | 842311 | 12090235 |
| P49429 | 4-hydroxyphenylpyruvate dioxygenase OS=Mus musculus OX=10090 GN=Hpd PE=1 SV=3 | 45 | 18 | 15 | 5 | 276537 | | 1146793 | 14795523 |
| Q11011 | Puromycin-sensitive aminopeptidase OS=Mus musculus OX=10090 GN=Npepps PE=1 SV=2 | 103.3 | 20 | 16 | 12 | 747888 | | 229620 | 8527032 |
| Q9DCD0 | 6-phosphogluconate dehydrogenase. decarboxylating OS=Mus musculus OX=10090 GN=Pgd PE=1 SV=3 | 53.2 | 20 | 12 | 6 | 12105805 | | 586862 | 10754455 |
| Q8R1G2 | Carboxymethylenebutenolidase homolog OS=Mus musculus OX=10090 GN=Cmbl PE=1 SV=1 | 27.9 | 23 | 28 | 7 | 577522 | | 743090 | 5567907 |
| O88544 | COP9 signalosome complex subunit 4 OS=Mus musculus OX=10090 GN=Cops4 PE=1 SV=1 | 46.3 | 28 | 14 | 8 | 387488 | | 326307 | 376832 |
| P29788 | Vitronectin OS=Mus musculus OX=10090 GN=Vtn PE=1 SV=2 | 54.8 | 16 | 52 | 5 | 11149725 | | 4517099 | 9929124 |
| Q99PT1 | Rho GDP-dissociation inhibitor 1 OS=Mus musculus OX=10090 GN=Arhgdia PE=1 SV=3 | 23.4 | 51 | 18 | 8 | 13490421 | | 1478222 | 20526122 |
| Q8CG16 | Complement C1r-A subcomponent OS=Mus musculus OX=10090 GN=C1ra PE=1 SV=1 | 80 | 10 | 48 | 4 | 1770791 | | 7691279 | 9046791 |
| P12382 | ATP-dependent 6-phosphofructokinase. liver type OS=Mus musculus OX=10090 GN=Pfkl PE=1 SV=4 | 85.3 | 14 | 23 | 7 | 1512954 | | 443330 | 1383656 |
| Q9QZ25 | Vascular non-inflammatory molecule 3 OS=Mus musculus OX=10090 GN=Vnn3 PE=1 SV=3 | 56.3 | 17 | 39 | 6 | 5495745 | | 4353340 | 3366100 |
| Q91WP6 | Serine protease inhibitor A3N OS=Mus musculus OX=10090 GN=Serpina3n PE=1 SV=1 | 46.7 | 8 | 38 | 1 | 63170513 | | 96596836 | 69106590 |
| O09172 | Glutamate--cysteine ligase regulatory subunit OS=Mus musculus OX=10090 GN=Gclm PE=1 SV=1 | 30.5 | 31 | 14 | 6 | 5526095 | | 87821 | 15920736 |
| Q9Z1Q5 | Chloride intracellular channel protein 1 OS=Mus musculus OX=10090 GN=Clic1 PE=1 SV=3 | 27 | 47 | 36 | 7 | 1007991 | | 1077496 | 16413195 |
| Q9R1P0 | Proteasome subunit alpha type-4 OS=Mus musculus OX=10090 GN=Psma4 PE=1 SV=1 | 29.5 | 30 | 21 | 7 | 6123948 | | 134592 | 4833639 |
| Q8VC30 | Triokinase/FMN cyclase OS=Mus musculus OX=10090 GN=Tkfc PE=1 SV=1 | 59.7 | 16 | 17 | 5 | 1068247 | |  | 9331961 |
| P01872 | Immunoglobulin heavy constant mu OS=Mus musculus OX=10090 GN=Ighm PE=1 SV=2 | 49.9 | 12 | 82 | 4 | 86633596 | | 55987652 | 96087533 |
| P02468 | Laminin subunit gamma-1 OS=Mus musculus OX=10090 GN=Lamc1 PE=1 SV=2 | 177.2 | 8 | 13 | 8 | 1486149 | | 1311746 | 172248 |
| P35822 | Receptor-type tyrosine-protein phosphatase kappa OS=Mus musculus OX=10090 GN=Ptprk PE=1 SV=1 | 164.1 | 9 | 9 | 8 | 205857 | | 139301 | 6260075 |
| P70441 | Na(+)/H(+) exchange regulatory cofactor NHE-RF1 OS=Mus musculus OX=10090 GN=Slc9a3r1 PE=1 SV=3 | 38.6 | 21 | 22 | 6 | 19503 | |  | 35067249 |
| P62806 | Histone H4 OS=Mus musculus OX=10090 GN=Hist1h4a PE=1 SV=2 | 11.4 | 42 | 30 | 5 | 1498704 | | 2854513 | 10054650 |
| O55234 | Proteasome subunit beta type-5 OS=Mus musculus OX=10090 GN=Psmb5 PE=1 SV=3 | 28.5 | 19 | 38 | 5 | 6095872 | | 337234 | 7612902 |
| P68510 | 14-3-3 protein eta OS=Mus musculus OX=10090 GN=Ywhah PE=1 SV=2 | 28.2 | 32 | 43 | 4 | 2693242 | | 1336656 | 4502209 |
| Q9R1P1 | Proteasome subunit beta type-3 OS=Mus musculus OX=10090 GN=Psmb3 PE=1 SV=1 | 22.9 | 27 | 19 | 4 | 1171811 | | 72113 | 2198688 |
| Q03734 | Serine protease inhibitor A3M OS=Mus musculus OX=10090 GN=Serpina3m PE=1 SV=2 | 47 | 11 | 71 | 3 | 150588867 | | 366370814 | 304461037 |
| Q8BG05 | Heterogeneous nuclear ribonucleoprotein A3 OS=Mus musculus OX=10090 GN=Hnrnpa3 PE=1 SV=1 | 39.6 | 20 | 17 | 5 | 1885422 | | 11403 | 1826051 |
| P35505 | Fumarylacetoacetase OS=Mus musculus OX=10090 GN=Fah PE=1 SV=2 | 46.1 | 19 | 20 | 5 | 604629 | | 482348 | 63393364 |
| Q93092 | Transaldolase OS=Mus musculus OX=10090 GN=Taldo1 PE=1 SV=2 | 37.4 | 24 | 14 | 7 | 1714197 | | 285072 | 22719748 |
| Q61171 | Peroxiredoxin-2 OS=Mus musculus OX=10090 GN=Prdx2 PE=1 SV=3 | 21.8 | 43 | 85 | 7 | 91416855 | | 10274003 | 26251625 |
| Q02357 | Ankyrin-1 OS=Mus musculus OX=10090 GN=Ank1 PE=1 SV=2 | 204.1 | 5 | 20 | 7 | 545600 | | 340379 | 529675 |
| P11352 | Glutathione peroxidase 1 OS=Mus musculus OX=10090 GN=Gpx1 PE=1 SV=2 | 22.3 | 42 | 80 | 8 | 280019976 | | 41707020 | 344280280 |
| O35945 | Aldehyde dehydrogenase. cytosolic 1 OS=Mus musculus OX=10090 GN=Aldh1a7 PE=1 SV=1 | 54.6 | 20 | 14 | 2 |  | |  | 2221209 |
| Q9D0M1 | Phosphoribosyl pyrophosphate synthase-associated protein 1 OS=Mus musculus OX=10090 GN=Prpsap1 PE=1 SV=1 | 39.4 | 34 | 15 | 8 | 442466 | | 15667 | 94713 |
| Q9QYC0 | Alpha-adducin OS=Mus musculus OX=10090 GN=Add1 PE=1 SV=2 | 80.6 | 18 | 14 | 7 | 277322 | | 57725 | 4520164 |
| A6X935 | Inter alpha-trypsin inhibitor. heavy chain 4 OS=Mus musculus OX=10090 GN=Itih4 PE=1 SV=2 | 104.6 | 7 | 72 | 6 | 33784960 | | 13084444 | 50671943 |
| P21550 | Beta-enolase OS=Mus musculus OX=10090 GN=Eno3 PE=1 SV=3 | 47 | 19 | 10 | 2 | 560536 | | 1332840 | 710456 |
| Q99K85 | Phosphoserine aminotransferase OS=Mus musculus OX=10090 GN=Psat1 PE=1 SV=1 | 40.4 | 18 | 7 | 5 | 206385 | |  | 27010634 |
| Q61646 | Haptoglobin OS=Mus musculus OX=10090 GN=Hp PE=1 SV=1 | 38.7 | 15 | 87 | 6 | 313982857 | | 428927798 | 303647422 |
| Q8CG14 | Complement C1s-A subcomponent OS=Mus musculus OX=10090 GN=C1sa PE=2 SV=2 | 76.8 | 6 | 16 | 4 | 987182 | | 4801290 | 5471704 |
| P70349 | Histidine triad nucleotide-binding protein 1 OS=Mus musculus OX=10090 GN=Hint1 PE=1 SV=3 | 13.8 | 71 | 23 | 5 | 46938 | |  | 9391600 |
| Q99KK7 | Dipeptidyl peptidase 3 OS=Mus musculus OX=10090 GN=Dpp3 PE=1 SV=2 | 82.8 | 14 | 12 | 5 | 674749 | | 201839 | 5326373 |
| P09103 | Protein disulfide-isomerase OS=Mus musculus OX=10090 GN=P4hb PE=1 SV=2 | 57 | 21 | 20 | 7 | 682247 | | 9843 | 12536393 |
| Q8VDK1 | Deaminated glutathione amidase OS=Mus musculus OX=10090 GN=Nit1 PE=1 SV=2 | 35.7 | 32 | 10 | 6 | 80228 | |  | 7912396 |
| Q9Z2U0 | Proteasome subunit alpha type-7 OS=Mus musculus OX=10090 GN=Psma7 PE=1 SV=1 | 27.8 | 29 | 27 | 6 | 5954977 | | 209429 | 4768515 |
| P53657 | Pyruvate kinase PKLR OS=Mus musculus OX=10090 GN=Pklr PE=1 SV=1 | 62.3 | 18 | 16 | 6 | 2641126 | | 151543 | 3139243 |
| Q8CC86 | Nicotinate phosphoribosyltransferase OS=Mus musculus OX=10090 GN=Naprt PE=1 SV=1 | 58.2 | 20 | 26 | 7 | 814268 | | 501436 | 19795818 |
| Q9R1P3 | Proteasome subunit beta type-2 OS=Mus musculus OX=10090 GN=Psmb2 PE=1 SV=1 | 22.9 | 24 | 25 | 5 | 4210133 | | 150890 | 3090617 |
| Q922U2 | Keratin. type II cytoskeletal 5 OS=Mus musculus OX=10090 GN=Krt5 PE=1 SV=1 | 61.7 | 9 | 49 | 3 | 25244 | | 390124 | 1034554 |
| P02535 | Keratin. type I cytoskeletal 10 OS=Mus musculus OX=10090 GN=Krt10 PE=1 SV=3 | 57.7 | 10 | 58 | 4 | 11583940 | | 17652971 | 13379291 |
| A2ASQ1 | Agrin OS=Mus musculus OX=10090 GN=Agrn PE=1 SV=1 | 207.4 | 5 | 11 | 6 | 65421 | | 2976 | 275414 |
| P70168 | Importin subunit beta-1 OS=Mus musculus OX=10090 GN=Kpnb1 PE=1 SV=2 | 97.1 | 10 | 18 | 6 | 1251627 | | 9540 | 582743 |
| O35350 | Calpain-1 catalytic subunit OS=Mus musculus OX=10090 GN=Capn1 PE=1 SV=1 | 82.1 | 13 | 20 | 7 | 765635 | |  | 792151 |
| Q9DC11 | Plexin domain-containing protein 2 OS=Mus musculus OX=10090 GN=Plxdc2 PE=1 SV=1 | 59.6 | 13 | 14 | 4 | 827830 | | 941203 | 2113395 |
| Q7TQ62 | Podocan OS=Mus musculus OX=10090 GN=Podn PE=2 SV=1 | 68.7 | 16 | 10 | 7 |  | | 25595 | 3427046 |
| Q6P069 | Sorcin OS=Mus musculus OX=10090 GN=Sri PE=1 SV=1 | 21.6 | 38 | 31 | 7 | 4635087 | | 574720 | 2477799 |
| P11087 | Collagen alpha-1(I) chain OS=Mus musculus OX=10090 GN=Col1a1 PE=1 SV=4 | 137.9 | 7 | 13 | 7 | 575113 | | 4768898 | 128431 |
| P23492 | Purine nucleoside phosphorylase OS=Mus musculus OX=10090 GN=Pnp PE=1 SV=2 | 32.3 | 16 | 16 | 4 | 14480668 | | 4396682 | 7910023 |
| P08730 | Keratin. type I cytoskeletal 13 OS=Mus musculus OX=10090 GN=Krt13 PE=1 SV=2 | 47.7 | 9 | 38 | 1 |  | |  | 541771 |
| P98064 | Mannan-binding lectin serine protease 1 OS=Mus musculus OX=10090 GN=Masp1 PE=1 SV=2 | 79.9 | 13 | 28 | 7 | 1492927 | | 2390312 | 2293001 |
| P21981 | Protein-glutamine gamma-glutamyltransferase 2 OS=Mus musculus OX=10090 GN=Tgm2 PE=1 SV=4 | 77 | 12 | 9 | 6 | 628006 | | 581164 | 1346065 |
| Q60692 | Proteasome subunit beta type-6 OS=Mus musculus OX=10090 GN=Psmb6 PE=1 SV=3 | 25.4 | 16 | 23 | 4 | 3392805 | | 55741 | 3171091 |
| P68040 | Receptor of activated protein C kinase 1 OS=Mus musculus OX=10090 GN=Rack1 PE=1 SV=3 | 35.1 | 23 | 17 | 4 | 109263 | | 25280 | 1758106 |
| Q61765 | Keratin. type I cuticular Ha1 OS=Mus musculus OX=10090 GN=Krt31 PE=1 SV=2 | 47.1 | 20 | 9 | 5 | 10176308 | |  | 91970 |
| Q8JZV9 | 3-hydroxybutyrate dehydrogenase type 2 OS=Mus musculus OX=10090 GN=Bdh2 PE=1 SV=1 | 26.7 | 32 | 5 | 5 |  | |  | 12984328 |
| Q61730 | Interleukin-1 receptor accessory protein OS=Mus musculus OX=10090 GN=Il1rap PE=1 SV=1 | 65.7 | 11 | 27 | 6 | 14067403 | | 13549932 | 13095364 |
| P21180 | Complement C2 OS=Mus musculus OX=10090 GN=C2 PE=1 SV=2 | 84.7 | 8 | 32 | 5 | 11594890 | | 11029803 | 12163815 |
| P97298 | Pigment epithelium-derived factor OS=Mus musculus OX=10090 GN=Serpinf1 PE=1 SV=2 | 46.2 | 21 | 10 | 8 | 2069322 | | 788202 | 17546009 |
| P07309 | Transthyretin OS=Mus musculus OX=10090 GN=Ttr PE=1 SV=1 | 15.8 | 42 | 62 | 3 | 49955905 | | 42631038 | 32455275 |
| Q6IRU2 | Tropomyosin alpha-4 chain OS=Mus musculus OX=10090 GN=Tpm4 PE=1 SV=3 | 28.5 | 17 | 16 | 4 | 4217918 | | 479962 | 8076805 |
| Q60994 | Adiponectin OS=Mus musculus OX=10090 GN=Adipoq PE=1 SV=2 | 26.8 | 12 | 53 | 3 | 14249931 | | 9525595 | 10010802 |
| P16675 | Lysosomal protective protein OS=Mus musculus OX=10090 GN=Ctsa PE=1 SV=1 | 53.8 | 15 | 13 | 5 | 1191589 | | 548250 | 4525786 |
| P31254 | Ubiquitin-like modifier-activating enzyme 1 Y OS=Mus musculus OX=10090 GN=Uba1y PE=1 SV=2 | 118 | 6 | 20 | 1 | 1726001 | | 200280 | 3061217 |
| P50446 | Keratin. type II cytoskeletal 6A OS=Mus musculus OX=10090 GN=Krt6a PE=1 SV=3 | 59.3 | 8 | 48 | 3 |  | | 513623 | 944059 |
| Q9JHR7 | Insulin-degrading enzyme OS=Mus musculus OX=10090 GN=Ide PE=1 SV=1 | 117.7 | 13 | 14 | 9 | 47360498 | | 17180097 | 259888738 |
| Q78PY7 | Staphylococcal nuclease domain-containing protein 1 OS=Mus musculus OX=10090 GN=Snd1 PE=1 SV=1 | 102 | 12 | 8 | 6 |  | |  | 2055555 |
| Q62179 | Semaphorin-4B OS=Mus musculus OX=10090 GN=Sema4b PE=1 SV=2 | 91.3 | 9 | 9 | 5 | 1028619 | | 78453 | 7618354 |
| Q60737 | Casein kinase II subunit alpha OS=Mus musculus OX=10090 GN=Csnk2a1 PE=1 SV=2 | 45.1 | 26 | 8 | 7 |  | |  | 1248667 |
| Q8BU25 | Inactive serine protease PAMR1 OS=Mus musculus OX=10090 GN=Pamr1 PE=2 SV=3 | 80.3 | 11 | 6 | 4 | 95316 | | 931642 | 1452492 |
| O88533 | Aromatic-L-amino-acid decarboxylase OS=Mus musculus OX=10090 GN=Ddc PE=1 SV=1 | 53.8 | 11 | 8 | 3 |  | | 841781 | 10591601 |
| P47968 | Ribose-5-phosphate isomerase OS=Mus musculus OX=10090 GN=Rpia PE=1 SV=2 | 32.4 | 29 | 7 | 6 | 12062279 | | 1049980 | 665139 |
| P11499 | Heat shock protein HSP 90-beta OS=Mus musculus OX=10090 GN=Hsp90ab1 PE=1 SV=3 | 83.2 | 10 | 11 | 6 |  | |  | 1154825 |
| P12023 | Amyloid-beta A4 protein OS=Mus musculus OX=10090 GN=App PE=1 SV=3 | 86.7 | 10 | 16 | 5 | 1255913 | | 194876 | 1620317 |
| P04104 | Keratin. type II cytoskeletal 1 OS=Mus musculus OX=10090 GN=Krt1 PE=1 SV=4 | 65.6 | 5 | 87 | 2 | 14771410 | | 16782029 | 14065848 |
| Q9JHH6 | Carboxypeptidase B2 OS=Mus musculus OX=10090 GN=Cpb2 PE=1 SV=1 | 48.8 | 11 | 27 | 6 | 22569033 | | 27814391 | 13827748 |
| Q62165 | Dystroglycan OS=Mus musculus OX=10090 GN=Dag1 PE=1 SV=4 | 96.8 | 8 | 10 | 5 |  | |  | 2181408 |
| P39876 | Metalloproteinase inhibitor 3 OS=Mus musculus OX=10090 GN=Timp3 PE=1 SV=1 | 24.2 | 29 | 20 | 5 | 819244 | | 702071 | 4791379 |
| Q9ET66 | Peptidase inhibitor 16 OS=Mus musculus OX=10090 GN=Pi16 PE=1 SV=2 | 53.6 | 11 | 16 | 3 | 708350 | | 1139706 | 1313704 |
| P06728 | Apolipoprotein A-IV OS=Mus musculus OX=10090 GN=Apoa4 PE=1 SV=3 | 45 | 9 | 41 | 3 | 567391284 | | 506034410 | 401597209 |
| P13634 | Carbonic anhydrase 1 OS=Mus musculus OX=10090 GN=Ca1 PE=1 SV=4 | 28.3 | 20 | 13 | 3 | 792964 | | 104180 | 18062 |
| P16331 | Phenylalanine-4-hydroxylase OS=Mus musculus OX=10090 GN=Pah PE=1 SV=4 | 51.9 | 20 | 9 | 6 |  | |  | 9477998 |
| Q91V64 | Isochorismatase domain-containing protein 1 OS=Mus musculus OX=10090 GN=Isoc1 PE=1 SV=1 | 32 | 28 | 6 | 5 |  | | 27404 | 5674837 |
| Q8R0Y6 | Cytosolic 10-formyltetrahydrofolate dehydrogenase OS=Mus musculus OX=10090 GN=Aldh1l1 PE=1 SV=1 | 98.6 | 8 | 6 | 5 |  | | 10836 | 300146 |
| Q8K182 | Complement component C8 alpha chain OS=Mus musculus OX=10090 GN=C8a PE=1 SV=1 | 66 | 5 | 13 | 2 | 1185698 | | 529100 | 4587665 |
| Q99JW2 | Aminoacylase-1 OS=Mus musculus OX=10090 GN=Acy1 PE=1 SV=1 | 45.8 | 17 | 12 | 5 | 95514 | | 80619 | 28603707 |
| Q3U0B3 | Dehydrogenase/reductase SDR family member 11 OS=Mus musculus OX=10090 GN=Dhrs11 PE=1 SV=1 | 28.3 | 24 | 13 | 5 | 1023837 | | 218275 | 142609 |
| Q9DB29 | Isoamyl acetate-hydrolyzing esterase 1 homolog OS=Mus musculus OX=10090 GN=Iah1 PE=1 SV=1 | 28 | 18 | 23 | 3 | 567422 | | 143035 | 12735101 |
| Q64726 | Zinc-alpha-2-glycoprotein OS=Mus musculus OX=10090 GN=Azgp1 PE=1 SV=2 | 35.3 | 20 | 21 | 5 | 27379401 | | 25274767 | 17755696 |
| Q91WP0 | Mannan-binding lectin serine protease 2 OS=Mus musculus OX=10090 GN=Masp2 PE=1 SV=1 | 75.5 | 8 | 12 | 4 | 77182136 | | 86836134 | 79242374 |
| P29621 | Serine protease inhibitor A3C OS=Mus musculus OX=10090 GN=Serpina3c PE=2 SV=1 | 46.7 | 6 | 37 | 1 | 99635811 | | 205509033 | 89105680 |
| P55264 | Adenosine kinase OS=Mus musculus OX=10090 GN=Adk PE=1 SV=2 | 40.1 | 23 | 6 | 5 | 487473 | | 129262 | 3297287 |
| Q9QWL7 | Keratin. type I cytoskeletal 17 OS=Mus musculus OX=10090 GN=Krt17 PE=1 SV=3 | 48.1 | 9 | 17 | 1 |  | |  |  |
| P61458 | Pterin-4-alpha-carbinolamine dehydratase OS=Mus musculus OX=10090 GN=Pcbd1 PE=1 SV=2 | 12 | 63 | 12 | 5 | 56735 | |  | 5988821 |
| Q9JMA1 | Ubiquitin carboxyl-terminal hydrolase 14 OS=Mus musculus OX=10090 GN=Usp14 PE=1 SV=3 | 56 | 15 | 5 | 4 | 101795 | | 46499 | 1854963 |
| P09411 | Phosphoglycerate kinase 1 OS=Mus musculus OX=10090 GN=Pgk1 PE=1 SV=4 | 44.5 | 20 | 18 | 6 | 15990 | | 15772 | 6470731 |
| Q8C0M9 | Isoaspartyl peptidase/L-asparaginase OS=Mus musculus OX=10090 GN=Asrgl1 PE=1 SV=1 | 33.9 | 21 | 6 | 4 |  | |  | 9252819 |
| P51855 | Glutathione synthetase OS=Mus musculus OX=10090 GN=Gss PE=1 SV=1 | 52.2 | 11 | 7 | 4 | 58606 | |  | 15995796 |
| O88569 | Heterogeneous nuclear ribonucleoproteins A2/B1 OS=Mus musculus OX=10090 GN=Hnrnpa2b1 PE=1 SV=2 | 37.4 | 14 | 13 | 5 | 50764 | | 32031 | 2862390 |
| Q8CG76 | Aflatoxin B1 aldehyde reductase member 2 OS=Mus musculus OX=10090 GN=Akr7a2 PE=1 SV=3 | 40.6 | 7 | 25 | 2 | 3229833 | | 116940 | 23432912 |
| P60843 | Eukaryotic initiation factor 4A-I OS=Mus musculus OX=10090 GN=Eif4a1 PE=1 SV=1 | 46.1 | 13 | 6 | 4 |  | | 37360 | 2567814 |
| Q8CB27 | Ubiquitin thioesterase OTU1 OS=Mus musculus OX=10090 GN=Yod1 PE=1 SV=1 | 37.5 | 15 | 11 | 4 | 369597 | |  |  |
| P01864 | Ig gamma-2A chain C region secreted form OS=Mus musculus OX=10090 PE=1 SV=1 | 36.6 | 13 | 50 | 1 | 18135507 | | 15751500 | 24287277 |
| O35381 | Acidic leucine-rich nuclear phosphoprotein 32 family member A OS=Mus musculus OX=10090 GN=Anp32a PE=1 SV=1 | 28.5 | 15 | 4 | 3 |  | |  | 10389681 |
| Q8BFY9 | Transportin-1 OS=Mus musculus OX=10090 GN=Tnpo1 PE=1 SV=2 | 102.3 | 7 | 5 | 5 | 115485 | | 198889 | 307095 |
| A2AJL3 | FGGY carbohydrate kinase domain-containing protein OS=Mus musculus OX=10090 GN=Fggy PE=1 SV=1 | 60.3 | 15 | 10 | 4 |  | |  | 1204971 |
| Q99KP3 | Lambda-crystallin homolog OS=Mus musculus OX=10090 GN=Cryl1 PE=1 SV=3 | 35.2 | 22 | 14 | 5 |  | |  | 46019275 |
| Q8CG03 | cGMP-specific 3'.5'-cyclic phosphodiesterase OS=Mus musculus OX=10090 GN=Pde5a PE=1 SV=2 | 98.3 | 7 | 10 | 5 | 854425 | | 2090417 | 3706895 |
| Q9CRC9 | Glucosamine-6-phosphate isomerase 2 OS=Mus musculus OX=10090 GN=Gnpda2 PE=1 SV=1 | 31.1 | 25 | 10 | 2 | 173631 | |  | 50892 |
| Q8VHY0 | Chondroitin sulfate proteoglycan 4 OS=Mus musculus OX=10090 GN=Cspg4 PE=1 SV=3 | 252.2 | 4 | 10 | 7 | 6888 | |  | 598512 |
| O08677 | Kininogen-1 OS=Mus musculus OX=10090 GN=Kng1 PE=1 SV=1 | 73.1 | 7 | 26 | 4 | 67922582 | | 66088619 | 81282718 |
| P40124 | Adenylyl cyclase-associated protein 1 OS=Mus musculus OX=10090 GN=Cap1 PE=1 SV=4 | 51.5 | 4 | 12 | 1 | 142690 | |  | 366067 |
| O88342 | WD repeat-containing protein 1 OS=Mus musculus OX=10090 GN=Wdr1 PE=1 SV=3 | 66.4 | 11 | 6 | 3 | 205347 | |  | 4221790 |
| P09528 | Ferritin heavy chain OS=Mus musculus OX=10090 GN=Fth1 PE=1 SV=2 | 21.1 | 24 | 25 | 4 | 5374799 | | 585492 | 7382749 |
| Q9WVJ3 | Carboxypeptidase Q OS=Mus musculus OX=10090 GN=Cpq PE=1 SV=1 | 51.8 | 12 | 24 | 4 | 13082046 | | 11033539 | 9709079 |
| Q05909 | Receptor-type tyrosine-protein phosphatase gamma OS=Mus musculus OX=10090 GN=Ptprg PE=1 SV=1 | 161.1 | 3 | 10 | 3 | 72682 | | 44530 | 853043 |
| Q6NXH9 | Keratin. type II cytoskeletal 73 OS=Mus musculus OX=10090 GN=Krt73 PE=1 SV=1 | 58.9 | 8 | 46 | 1 |  | |  | 73857 |
| P70663 | SPARC-like protein 1 OS=Mus musculus OX=10090 GN=Sparcl1 PE=1 SV=3 | 72.2 | 10 | 7 | 5 | 14566 | |  | 619374 |
| P63005 | Platelet-activating factor acetylhydrolase IB subunit alpha OS=Mus musculus OX=10090 GN=Pafah1b1 PE=1 SV=2 | 46.6 | 14 | 11 | 4 | 640979 | | 18485 | 2997886 |
| Q63836 | Selenium-binding protein 2 OS=Mus musculus OX=10090 GN=Selenbp2 PE=1 SV=2 | 52.6 | 15 | 13 | 5 | 12039535 | |  | 6802183 |
| O35988 | Syndecan-4 OS=Mus musculus OX=10090 GN=Sdc4 PE=1 SV=1 | 21.5 | 15 | 27 | 3 | 13830648 | | 94440 | 19229397 |
| P97290 | Plasma protease C1 inhibitor OS=Mus musculus OX=10090 GN=Serping1 PE=1 SV=3 | 55.5 | 6 | 43 | 3 | 52855334 | | 76820935 | 63435456 |
| Q6IFX2 | Keratin. type I cytoskeletal 42 OS=Mus musculus OX=10090 GN=Krt42 PE=1 SV=1 | 50.1 | 7 | 11 | 1 |  | |  | 826646 |
| Q8VCC2 | Liver carboxylesterase 1 OS=Mus musculus OX=10090 GN=Ces1 PE=1 SV=1 | 62.6 | 12 | 29 | 3 | 78844875 | | 51178064 | 48800753 |
| Q9CXF0 | Kynureninase OS=Mus musculus OX=10090 GN=Kynu PE=1 SV=3 | 52.3 | 13 | 5 | 4 | 107554 | | 91229 | 5141224 |
| P50431 | Serine hydroxymethyltransferase. cytosolic OS=Mus musculus OX=10090 GN=Shmt1 PE=1 SV=3 | 52.6 | 11 | 4 | 4 |  | |  | 3461630 |
| P01865 | Ig gamma-2A chain C region. membrane-bound form OS=Mus musculus OX=10090 GN=Igh-1a PE=1 SV=3 | 43.9 | 8 | 45 | 1 | 564098 | | 339358 | 624325 |
| Q9D646 | Keratin. type I cuticular Ha4 OS=Mus musculus OX=10090 GN=Krt34 PE=2 SV=1 | 44.5 | 11 | 5 | 1 | 3982407 | | 48375 |  |
| P15327 | Bisphosphoglycerate mutase OS=Mus musculus OX=10090 GN=Bpgm PE=1 SV=2 | 30 | 21 | 12 | 5 | 32904655 | | 4076802 | 11446325 |
| P24549 | Retinal dehydrogenase 1 OS=Mus musculus OX=10090 GN=Aldh1a1 PE=1 SV=5 | 54.4 | 13 | 8 | 1 |  | |  | 333335 |
| P62827 | GTP-binding nuclear protein Ran OS=Mus musculus OX=10090 GN=Ran PE=1 SV=3 | 24.4 | 31 | 23 | 5 | 2390772 | | 696652 | 2745906 |
| Q8BVI4 | Dihydropteridine reductase OS=Mus musculus OX=10090 GN=Qdpr PE=1 SV=2 | 25.6 | 18 | 6 | 3 | 1840196 | |  | 27396648 |
| Q99LS3 | Phosphoserine phosphatase OS=Mus musculus OX=10090 GN=Psph PE=1 SV=1 | 25.1 | 17 | 4 | 3 | 1008531 | |  | 6240684 |
| Q78JT3 | 3-hydroxyanthranilate 3.4-dioxygenase OS=Mus musculus OX=10090 GN=Haao PE=1 SV=1 | 32.8 | 19 | 13 | 4 | 5903 | |  | 2990934 |
| Q61129 | Complement factor I OS=Mus musculus OX=10090 GN=Cfi PE=1 SV=3 | 67.2 | 10 | 31 | 4 | 20783687 | | 55111705 | 72855637 |
| Q91ZJ5 | UTP--glucose-1-phosphate uridylyltransferase OS=Mus musculus OX=10090 GN=Ugp2 PE=1 SV=3 | 56.9 | 13 | 6 | 5 | 411905 | | 137904 | 926122 |
| Q91V92 | ATP-citrate synthase OS=Mus musculus OX=10090 GN=Acly PE=1 SV=1 | 119.7 | 5 | 7 | 4 | 6910 | |  | 5749 |
| O70570 | Polymeric immunoglobulin receptor OS=Mus musculus OX=10090 GN=Pigr PE=1 SV=1 | 84.9 | 6 | 35 | 4 | 6780908 | | 14755167 | 11241423 |
| P47754 | F-actin-capping protein subunit alpha-2 OS=Mus musculus OX=10090 GN=Capza2 PE=1 SV=3 | 32.9 | 29 | 6 | 4 | 20029 | |  | 956986 |
| P08113 | Endoplasmin OS=Mus musculus OX=10090 GN=Hsp90b1 PE=1 SV=2 | 92.4 | 6 | 8 | 3 | 264266 | | 115939 | 804282 |
| P15105 | Glutamine synthetase OS=Mus musculus OX=10090 GN=Glul PE=1 SV=6 | 42.1 | 10 | 6 | 2 | 267593 | | 14252 | 1323242 |
| Q8R121 | Protein Z-dependent protease inhibitor OS=Mus musculus OX=10090 GN=Serpina10 PE=1 SV=1 | 51.8 | 7 | 44 | 5 | 16874732 | | 21106570 | 21992205 |
| P54726 | UV excision repair protein RAD23 homolog A OS=Mus musculus OX=10090 GN=Rad23a PE=1 SV=2 | 39.7 | 33 | 18 | 4 | 4293941 | | 309216 | 3372242 |
| Q64523 | Histone H2A type 2-C OS=Mus musculus OX=10090 GN=Hist2h2ac PE=1 SV=3 | 14 | 22 | 17 | 2 | 167614 | | 60297 | 37568 |
| Q80W22 | Threonine synthase-like 2 OS=Mus musculus OX=10090 GN=Thnsl2 PE=1 SV=1 | 54.2 | 11 | 9 | 3 | 670433 | | 791230 | 4256590 |
| P97372 | Proteasome activator complex subunit 2 OS=Mus musculus OX=10090 GN=Psme2 PE=1 SV=4 | 27 | 21 | 3 | 3 |  | |  | 868210 |
| Q9CZT5 | Vasorin OS=Mus musculus OX=10090 GN=Vasn PE=2 SV=2 | 72.2 | 10 | 13 | 4 | 980170 | | 338624 | 2575064 |
| Q80W65 | Proprotein convertase subtilisin/kexin type 9 OS=Mus musculus OX=10090 GN=Pcsk9 PE=1 SV=2 | 74.8 | 6 | 9 | 3 |  | | 193413 | 113412 |
| Q07968 | Coagulation factor XIII B chain OS=Mus musculus OX=10090 GN=F13b PE=1 SV=2 | 76.1 | 7 | 19 | 4 | 7862073 | | 7155129 | 7121756 |
| Q08761 | Vitamin K-dependent protein S OS=Mus musculus OX=10090 GN=Pros1 PE=2 SV=1 | 74.9 | 7 | 18 | 4 | 997806 | | 423004 | 4508839 |
| P35700 | Peroxiredoxin-1 OS=Mus musculus OX=10090 GN=Prdx1 PE=1 SV=1 | 22.2 | 32 | 37 | 5 | 36935288 | | 16475301 | 46379043 |
| P01942 | Hemoglobin subunit alpha OS=Mus musculus OX=10090 GN=Hba PE=1 SV=2 | 15.1 | 21 | 117 | 3 | 906438591 | | 221385816 | 648208927 |
| P13595 | Neural cell adhesion molecule 1 OS=Mus musculus OX=10090 GN=Ncam1 PE=1 SV=3 | 119.4 | 8 | 11 | 5 | 865177 | | 1268808 | 808833 |
| Q9DBB9 | Carboxypeptidase N subunit 2 OS=Mus musculus OX=10090 GN=Cpn2 PE=1 SV=2 | 60.4 | 5 | 27 | 3 | 9375970 | | 14016351 | 15935623 |
| P23953 | Carboxylesterase 1C OS=Mus musculus OX=10090 GN=Ces1c PE=1 SV=4 | 61 | 6 | 21 | 2 | 39547272 | | 37066030 | 26152732 |
| Q9QYB8 | Beta-adducin OS=Mus musculus OX=10090 GN=Add2 PE=1 SV=4 | 80.6 | 6 | 8 | 3 | 454182 | | 170447 | 1933550 |
| P07744 | Keratin. type II cytoskeletal 4 OS=Mus musculus OX=10090 GN=Krt4 PE=1 SV=2 | 56.2 | 6 | 26 | 2 | 141767844 | | 183156482 | 933902 |
| Q9R0P3 | S-formylglutathione hydrolase OS=Mus musculus OX=10090 GN=Esd PE=1 SV=1 | 31.3 | 19 | 11 | 3 | 110640 | | 678167 | 438478 |
| P15116 | Cadherin-2 OS=Mus musculus OX=10090 GN=Cdh2 PE=1 SV=2 | 99.7 | 9 | 4 | 3 | 45578 | | 65447 | 564016 |
| P39061 | Collagen alpha-1(XVIII) chain OS=Mus musculus OX=10090 GN=Col18a1 PE=1 SV=4 | 182.1 | 3 | 14 | 5 | 335745 | | 433880 | 1183567 |
| P14106 | Complement C1q subcomponent subunit B OS=Mus musculus OX=10090 GN=C1qb PE=1 SV=2 | 26.7 | 11 | 10 | 2 | 396714 | |  | 1026831 |
| A6H6E2 | Multimerin-2 OS=Mus musculus OX=10090 GN=Mmrn2 PE=1 SV=1 | 105.1 | 3 | 3 | 2 |  | |  | 918021 |
| P26443 | Glutamate dehydrogenase 1. mitochondrial OS=Mus musculus OX=10090 GN=Glud1 PE=1 SV=1 | 61.3 | 8 | 6 | 4 | 698074 | | 494844 | 793522 |
| P70194 | C-type lectin domain family 4 member F OS=Mus musculus OX=10090 GN=Clec4f PE=1 SV=1 | 61.2 | 5 | 5 | 2 | 249131 | |  |  |
| Q99J99 | 3-mercaptopyruvate sulfurtransferase OS=Mus musculus OX=10090 GN=Mpst PE=1 SV=4 | 33.1 | 13 | 9 | 3 | 385683 | | 153673 | 72722 |
| Q6WVG3 | BTB/POZ domain-containing protein KCTD12 OS=Mus musculus OX=10090 GN=Kctd12 PE=1 SV=1 | 35.9 | 12 | 11 | 3 | 263827 | | 107719 | 340282 |
| O70362 | Phosphatidylinositol-glycan-specific phospholipase D OS=Mus musculus OX=10090 GN=Gpld1 PE=1 SV=1 | 93.2 | 7 | 9 | 4 | 840685 | | 323781 | 1506908 |
| Q8VED5 | Keratin. type II cytoskeletal 79 OS=Mus musculus OX=10090 GN=Krt79 PE=1 SV=2 | 57.5 | 6 | 37 | 1 | 1854691 | | 2581105 | 1851534 |
| Q9CXA2 | Trans-L-3-hydroxyproline dehydratase OS=Mus musculus OX=10090 GN=L3hypdh PE=1 SV=1 | 37.8 | 16 | 9 | 4 |  | |  | 1148245 |
| Q9D939 | Sulfotransferase 1C2 OS=Mus musculus OX=10090 GN=Sult1c2 PE=1 SV=1 | 34.9 | 16 | 10 | 4 | 348265 | | 193246 | 16677444 |
| P62984 | Ubiquitin-60S ribosomal protein L40 OS=Mus musculus OX=10090 GN=Uba52 PE=1 SV=2 | 14.7 | 27 | 39 | 3 | 19106808 | | 675930 | 23895603 |
| O08742 | Platelet glycoprotein V OS=Mus musculus OX=10090 GN=Gp5 PE=1 SV=1 | 63.4 | 3 | 5 | 1 | 938980 | | 438514 | 955815 |
| Q61772 | Ephrin type-A receptor 7 OS=Mus musculus OX=10090 GN=Epha7 PE=1 SV=2 | 111.8 | 3 | 2 | 2 |  | | 20144 | 543274 |
| P10126 | Elongation factor 1-alpha 1 OS=Mus musculus OX=10090 GN=Eef1a1 PE=1 SV=3 | 50.1 | 14 | 9 | 3 | 6419 | | 116724 | 119015 |
| Q9CPU0 | Lactoylglutathione lyase OS=Mus musculus OX=10090 GN=Glo1 PE=1 SV=3 | 20.8 | 34 | 9 | 4 | 17151948 | | 2217952 | 16825714 |
| P26040 | Ezrin OS=Mus musculus OX=10090 GN=Ezr PE=1 SV=3 | 69.4 | 6 | 5 | 4 |  | |  | 4647165 |
| P97822 | Acidic leucine-rich nuclear phosphoprotein 32 family member E OS=Mus musculus OX=10090 GN=Anp32e PE=1 SV=2 | 29.6 | 13 | 5 | 2 |  | |  | 449684 |
| Q64449 | C-type mannose receptor 2 OS=Mus musculus OX=10090 GN=Mrc2 PE=1 SV=3 | 167 | 3 | 4 | 3 | 876418 | |  | 81254 |
| Q80X90 | Filamin-B OS=Mus musculus OX=10090 GN=Flnb PE=1 SV=3 | 277.7 | 2 | 5 | 3 |  | |  | 75246 |
| Q8R2Y2 | Cell surface glycoprotein MUC18 OS=Mus musculus OX=10090 GN=Mcam PE=1 SV=1 | 71.5 | 6 | 7 | 3 | 320844 | |  | 2409847 |
| Q07456 | Protein AMBP OS=Mus musculus OX=10090 GN=Ambp PE=1 SV=2 | 39 | 4 | 45 | 1 | 56699963 | | 75709675 | 70317331 |
| O35206 | Collagen alpha-1(XV) chain OS=Mus musculus OX=10090 GN=Col15a1 PE=1 SV=2 | 140.4 | 2 | 3 | 2 | 12083764 | | 8336392 | 236860 |
| P10493 | Nidogen-1 OS=Mus musculus OX=10090 GN=Nid1 PE=1 SV=2 | 136.5 | 4 | 8 | 4 | 221180 | | 122149 | 354310 |
| Q07797 | Galectin-3-binding protein OS=Mus musculus OX=10090 GN=Lgals3bp PE=1 SV=1 | 64.5 | 9 | 11 | 4 | 1365281 | | 830111 | 704811 |
| P10639 | Thioredoxin OS=Mus musculus OX=10090 GN=Txn PE=1 SV=3 | 11.7 | 32 | 17 | 3 | 17070379 | | 2850192 | 19140804 |
| P26262 | Plasma kallikrein OS=Mus musculus OX=10090 GN=Klkb1 PE=1 SV=2 | 71.3 | 7 | 7 | 3 | 11662723 | | 4444424 | 2880049 |
| P17742 | Peptidyl-prolyl cis-trans isomerase A OS=Mus musculus OX=10090 GN=Ppia PE=1 SV=2 | 18 | 30 | 5 | 3 | 700677 | | 603544 | 697298 |
| P16015 | Carbonic anhydrase 3 OS=Mus musculus OX=10090 GN=Ca3 PE=1 SV=3 | 29.3 | 14 | 11 | 3 |  | |  | 126720 |
| P47753 | F-actin-capping protein subunit alpha-1 OS=Mus musculus OX=10090 GN=Capza1 PE=1 SV=4 | 32.9 | 16 | 4 | 2 | 383593 | | 56824 | 2218544 |
| P07310 | Creatine kinase M-type OS=Mus musculus OX=10090 GN=Ckm PE=1 SV=1 | 43 | 9 | 2 | 2 |  | |  |  |
| Q3U1J4 | DNA damage-binding protein 1 OS=Mus musculus OX=10090 GN=Ddb1 PE=1 SV=2 | 126.8 | 5 | 7 | 4 | 56085 | |  | 730382 |
| Q9R098 | Hepatocyte growth factor activator OS=Mus musculus OX=10090 GN=Hgfac PE=1 SV=1 | 70.5 | 5 | 13 | 2 | 4265892 | | 5565040 | 3806794 |
| P49222 | Erythrocyte membrane protein band 4.2 OS=Mus musculus OX=10090 GN=Epb42 PE=1 SV=3 | 76.7 | 7 | 6 | 3 | 62766 | |  | 444690 |
| O70165 | Ficolin-1 OS=Mus musculus OX=10090 GN=Fcn1 PE=1 SV=1 | 36.3 | 5 | 9 | 1 |  | | 124183 | 788227 |
| P24452 | Macrophage-capping protein OS=Mus musculus OX=10090 GN=Capg PE=1 SV=2 | 39.2 | 8 | 2 | 2 | 161213 | | 18472 | 106862 |
| Q02257 | Junction plakoglobin OS=Mus musculus OX=10090 GN=Jup PE=1 SV=3 | 81.7 | 6 | 4 | 4 |  | | 166600 |  |
| Q61508 | Extracellular matrix protein 1 OS=Mus musculus OX=10090 GN=Ecm1 PE=1 SV=2 | 62.8 | 5 | 4 | 2 | 608680 | | 452628 | 994129 |
| Q9R069 | Basal cell adhesion molecule OS=Mus musculus OX=10090 GN=Bcam PE=1 SV=1 | 67.6 | 6 | 4 | 2 | 148663 | | 59827 | 540280 |
| Q9D5J6 | Sedoheptulokinase OS=Mus musculus OX=10090 GN=Shpk PE=1 SV=1 | 51.3 | 8 | 5 | 3 | 45905 | | 124338 | 1343427 |
| Q9ET22 | Dipeptidyl peptidase 2 OS=Mus musculus OX=10090 GN=Dpp7 PE=1 SV=2 | 56.2 | 7 | 6 | 3 | 1108922 | | 96996 | 1665123 |
| Q9D1A2 | Cytosolic non-specific dipeptidase OS=Mus musculus OX=10090 GN=Cndp2 PE=1 SV=1 | 52.7 | 13 | 6 | 4 | 56555 | | 88210 | 1547282 |
| Q8CIF4 | Biotinidase OS=Mus musculus OX=10090 GN=Btd PE=1 SV=2 | 58.1 | 3 | 5 | 1 | 656233 | | 528628 | 965712 |
| P54822 | Adenylosuccinate lyase OS=Mus musculus OX=10090 GN=Adsl PE=1 SV=2 | 54.8 | 11 | 13 | 3 | 563915 | |  | 291651 |
| P00493 | Hypoxanthine-guanine phosphoribosyltransferase OS=Mus musculus OX=10090 GN=Hprt1 PE=1 SV=3 | 24.6 | 18 | 6 | 3 | 9841550 | |  | 4247785 |
| Q91X17 | Uromodulin OS=Mus musculus OX=10090 GN=Umod PE=1 SV=1 | 70.8 | 5 | 17 | 3 | 31289484 | | 3756595 | 31751302 |
| P98156 | Very low-density lipoprotein receptor OS=Mus musculus OX=10090 GN=Vldlr PE=1 SV=1 | 96.3 | 4 | 3 | 2 | 27101 | |  | 89141 |
| Q91V76 | Ester hydrolase C11orf54 homolog OS=Mus musculus OX=10090 PE=1 SV=1 | 35 | 12 | 13 | 3 | 62001 | | 292686 | 7301477 |
| P28653 | Biglycan OS=Mus musculus OX=10090 GN=Bgn PE=1 SV=1 | 41.6 | 15 | 8 | 4 | 26893 | |  | 707800 |
| P10107 | Annexin A1 OS=Mus musculus OX=10090 GN=Anxa1 PE=1 SV=2 | 38.7 | 10 | 3 | 3 |  | |  | 193079 |
| Q91V98 | Endosialin OS=Mus musculus OX=10090 GN=Cd248 PE=1 SV=1 | 81.8 | 8 | 7 | 3 | 168133 | | 34322 | 236121 |
| P28654 | Decorin OS=Mus musculus OX=10090 GN=Dcn PE=1 SV=1 | 39.8 | 6 | 10 | 2 | 451496 | |  | 3132872 |
| Q8BKG3 | Inactive tyrosine-protein kinase 7 OS=Mus musculus OX=10090 GN=Ptk7 PE=1 SV=1 | 117.5 | 2 | 2 | 2 | 602885 | | 111476 | 139690 |
| Q76MZ3 | Serine/threonine-protein phosphatase 2A 65 kDa regulatory subunit A alpha isoform OS=Mus musculus OX=10090 GN=Ppp2r1a PE=1 SV=3 | 65.3 | 8 | 12 | 3 | 529635 | |  | 1044487 |
| Q9CS42 | Ribose-phosphate pyrophosphokinase 2 OS=Mus musculus OX=10090 GN=Prps2 PE=1 SV=4 | 34.8 | 14 | 8 | 3 | 508416 | | 18793 | 148122 |
| Q64176 | Carboxylesterase 1E OS=Mus musculus OX=10090 GN=Ces1e PE=1 SV=1 | 61.5 | 4 | 18 | 1 | 60166169 | | 59351798 | 39898675 |
| Q9D358 | Low molecular weight phosphotyrosine protein phosphatase OS=Mus musculus OX=10090 GN=Acp1 PE=1 SV=3 | 18.2 | 17 | 11 | 2 | 1250958 | | 834177 | 846766 |
| Q9EQU5 | Protein SET OS=Mus musculus OX=10090 GN=Set PE=1 SV=1 | 33.4 | 15 | 2 | 2 |  | |  | 599119 |
| Q8C255 | Dipeptidase 2 OS=Mus musculus OX=10090 GN=Dpep2 PE=1 SV=1 | 52.6 | 8 | 7 | 3 | 382100 | | 96160 | 764552 |
| P52480 | Pyruvate kinase PKM OS=Mus musculus OX=10090 GN=Pkm PE=1 SV=4 | 57.8 | 7 | 7 | 2 |  | |  |  |
| P97371 | Proteasome activator complex subunit 1 OS=Mus musculus OX=10090 GN=Psme1 PE=1 SV=2 | 28.7 | 16 | 3 | 3 | 401410 | | 97481 | 1888822 |
| Q9R0Q7 | Prostaglandin E synthase 3 OS=Mus musculus OX=10090 GN=Ptges3 PE=1 SV=1 | 18.7 | 26 | 4 | 3 |  | | 2634534 | 6884537 |
| Q9CQW3 | Vitamin K-dependent protein Z OS=Mus musculus OX=10090 GN=Proz PE=1 SV=1 | 44.3 | 5 | 4 | 1 | 241901 | | 41297 | 114535 |
| P13745 | Glutathione S-transferase A1 OS=Mus musculus OX=10090 GN=Gsta1 PE=1 SV=2 | 25.6 | 17 | 8 | 3 |  | |  | 17059105 |
| O54890 | Integrin beta-3 OS=Mus musculus OX=10090 GN=Itgb3 PE=1 SV=2 | 86.7 | 5 | 3 | 3 | 75619 | |  |  |
| O89112 | Glutathione S-transferase LANCL1 OS=Mus musculus OX=10090 GN=Lancl1 PE=1 SV=1 | 45.3 | 9 | 12 | 3 | 547126 | | 264488 | 1188453 |
| P70333 | Heterogeneous nuclear ribonucleoprotein H2 OS=Mus musculus OX=10090 GN=Hnrnph2 PE=1 SV=1 | 49.2 | 6 | 11 | 2 | 87318 | | 8860 | 320161 |
| P97333 | Neuropilin-1 OS=Mus musculus OX=10090 GN=Nrp1 PE=1 SV=2 | 102.9 | 4 | 2 | 2 |  | |  |  |
| P61202 | COP9 signalosome complex subunit 2 OS=Mus musculus OX=10090 GN=Cops2 PE=1 SV=1 | 51.6 | 9 | 4 | 3 | 17149 | |  | 256281 |
| Q8BH00 | 2-aminomuconic semialdehyde dehydrogenase OS=Mus musculus OX=10090 GN=Aldh8a1 PE=1 SV=1 | 53.6 | 6 | 7 | 2 |  | | 20860 | 331314 |
| P18760 | Cofilin-1 OS=Mus musculus OX=10090 GN=Cfl1 PE=1 SV=3 | 18.5 | 15 | 5 | 2 |  | |  | 205654 |
| Q8VCS0 | N-acetylmuramoyl-L-alanine amidase OS=Mus musculus OX=10090 GN=Pglyrp2 PE=1 SV=1 | 57.7 | 3 | 10 | 1 | 3373712 | | 11520546 | 12265837 |
| P47757 | F-actin-capping protein subunit beta OS=Mus musculus OX=10090 GN=Capzb PE=1 SV=3 | 31.3 | 21 | 5 | 4 |  | |  | 1213160 |
| O88545 | COP9 signalosome complex subunit 6 OS=Mus musculus OX=10090 GN=Cops6 PE=1 SV=1 | 35.9 | 11 | 3 | 3 |  | |  | 67656 |
| O35864 | COP9 signalosome complex subunit 5 OS=Mus musculus OX=10090 GN=Cops5 PE=1 SV=3 | 37.5 | 15 | 4 | 4 | 55385 | |  | 299303 |
| Q8CIZ8 | von Willebrand factor OS=Mus musculus OX=10090 GN=Vwf PE=1 SV=2 | 309.1 | 1 | 4 | 2 | 700052 | | 666572 | 1672985 |
| Q9Z2L7 | Cytokine receptor-like factor 3 OS=Mus musculus OX=10090 GN=Crlf3 PE=1 SV=1 | 49.5 | 9 | 2 | 2 |  | | 27558 | 40353 |
| P28063 | Proteasome subunit beta type-8 OS=Mus musculus OX=10090 GN=Psmb8 PE=1 SV=2 | 30.2 | 9 | 4 | 3 | 232547 | |  | 148400 |
| P54728 | UV excision repair protein RAD23 homolog B OS=Mus musculus OX=10090 GN=Rad23b PE=1 SV=2 | 43.5 | 7 | 2 | 2 | 189791 | |  | 387184 |
| Q19LI2 | Alpha-1B-glycoprotein OS=Mus musculus OX=10090 GN=A1bg PE=1 SV=1 | 56.5 | 4 | 97 | 3 | 500823056 | | 396406670 | 208547972 |
| P34884 | Macrophage migration inhibitory factor OS=Mus musculus OX=10090 GN=Mif PE=1 SV=2 | 12.5 | 17 | 8 | 2 | 151906 | | 77067 | 2438700 |
| Q91XE4 | N-acyl-aromatic-L-amino acid amidohydrolase (carboxylate-forming) OS=Mus musculus OX=10090 GN=Acy3 PE=1 SV=1 | 35.3 | 9 | 5 | 2 |  | |  | 10946039 |
| Q9CQM9 | Glutaredoxin-3 OS=Mus musculus OX=10090 GN=Glrx3 PE=1 SV=1 | 37.8 | 11 | 7 | 3 |  | | 16869 | 1580347 |
| O70591 | Prefoldin subunit 2 OS=Mus musculus OX=10090 GN=Pfdn2 PE=1 SV=2 | 16.5 | 17 | 3 | 2 | 1687254 | | 1012844 | 1403930 |
| Q6IME9 | Keratin. type II cytoskeletal 72 OS=Mus musculus OX=10090 GN=Krt72 PE=3 SV=1 | 56.7 | 4 | 28 | 1 | 198635 | | 485292 | 479031 |
| Q9WU28 | Prefoldin subunit 5 OS=Mus musculus OX=10090 GN=Pfdn5 PE=1 SV=1 | 17.3 | 21 | 4 | 2 | 148308 | | 23384 | 741443 |
| Q64433 | 10 kDa heat shock protein. mitochondrial OS=Mus musculus OX=10090 GN=Hspe1 PE=1 SV=2 | 11 | 25 | 2 | 2 |  | |  | 2711110 |
| P62715 | Serine/threonine-protein phosphatase 2A catalytic subunit beta isoform OS=Mus musculus OX=10090 GN=Ppp2cb PE=1 SV=1 | 35.6 | 8 | 7 | 2 | 145947 | | 31483 | 25636 |
| P97326 | Cadherin-6 OS=Mus musculus OX=10090 GN=Cdh6 PE=1 SV=2 | 88.3 | 6 | 5 | 3 |  | |  | 1591044 |
| P02104 | Hemoglobin subunit epsilon-Y2 OS=Mus musculus OX=10090 GN=Hbb-y PE=1 SV=2 | 16.1 | 12 | 61 | 1 | 665209 | | 469260 | 459801 |
| P02469 | Laminin subunit beta-1 OS=Mus musculus OX=10090 GN=Lamb1 PE=1 SV=3 | 197 | 2 | 5 | 3 | 124897 | | 82024 | 297165 |
| P25444 | 40S ribosomal protein S2 OS=Mus musculus OX=10090 GN=Rps2 PE=1 SV=3 | 31.2 | 9 | 5 | 2 |  | |  | 97494 |
| P67871 | Casein kinase II subunit beta OS=Mus musculus OX=10090 GN=Csnk2b PE=1 SV=1 | 24.9 | 12 | 2 | 2 |  | |  | 434692 |
| Q62468 | Villin-1 OS=Mus musculus OX=10090 GN=Vil1 PE=1 SV=3 | 92.7 | 5 | 3 | 3 |  | |  | 1611185 |
| Q9QZZ6 | Dermatopontin OS=Mus musculus OX=10090 GN=Dpt PE=1 SV=1 | 24 | 12 | 6 | 2 | 754116 | | 542009 | 309451 |
| Q9JHQ5 | Leucine zipper transcription factor-like protein 1 OS=Mus musculus OX=10090 GN=Lztfl1 PE=1 SV=1 | 34.8 | 9 | 2 | 2 |  | |  | 821345 |
| O54983 | Ketimine reductase mu-crystallin OS=Mus musculus OX=10090 GN=Crym PE=1 SV=1 | 33.5 | 12 | 7 | 2 |  | |  | 139027 |
| P40336 | Vacuolar protein sorting-associated protein 26A OS=Mus musculus OX=10090 GN=Vps26a PE=1 SV=1 | 38.1 | 9 | 2 | 2 |  | |  | 170893 |
| P52430 | Serum paraoxonase/arylesterase 1 OS=Mus musculus OX=10090 GN=Pon1 PE=1 SV=2 | 39.5 | 3 | 15 | 1 | 3263233 | | 1006906 | 7001164 |
| P70296 | Phosphatidylethanolamine-binding protein 1 OS=Mus musculus OX=10090 GN=Pebp1 PE=1 SV=3 | 20.8 | 20 | 9 | 2 |  | | 5897 | 3128712 |
| P46664 | Adenylosuccinate synthetase isozyme 2 OS=Mus musculus OX=10090 GN=Adss PE=1 SV=2 | 50 | 6 | 2 | 2 | 200515 | | 273297 | 1021512 |
| O88947 | Coagulation factor X OS=Mus musculus OX=10090 GN=F10 PE=1 SV=1 | 54 | 6 | 10 | 3 | 7895350 | | 456801 | 6590597 |
| Q9EST5 | Acidic leucine-rich nuclear phosphoprotein 32 family member B OS=Mus musculus OX=10090 GN=Anp32b PE=1 SV=1 | 31.1 | 11 | 3 | 2 |  | |  | 218639 |
| Q6A4J8 | Ubiquitin carboxyl-terminal hydrolase 7 OS=Mus musculus OX=10090 GN=Usp7 PE=1 SV=1 | 128.4 | 5 | 3 | 3 | 266142 | | 295756 | 230545 |
| P13707 | Glycerol-3-phosphate dehydrogenase [NAD(+)]. cytoplasmic OS=Mus musculus OX=10090 GN=Gpd1 PE=1 SV=3 | 37.5 | 11 | 7 | 3 |  | |  | 1085269 |
| Q64442 | Sorbitol dehydrogenase OS=Mus musculus OX=10090 GN=Sord PE=1 SV=3 | 38.2 | 9 | 10 | 3 | 3038434 | |  | 1751656 |
| O88543 | COP9 signalosome complex subunit 3 OS=Mus musculus OX=10090 GN=Cops3 PE=1 SV=3 | 47.8 | 9 | 2 | 2 |  | |  |  |
| P48193 | Protein 4.1 OS=Mus musculus OX=10090 GN=Epb41 PE=1 SV=2 | 95.9 | 5 | 11 | 3 | 7687705 | | 550658 | 1742581 |
| Q8BK48 | Pyrethroid hydrolase Ces2e OS=Mus musculus OX=10090 GN=Ces2e PE=1 SV=1 | 62.3 | 6 | 3 | 3 | 1963255 | |  | 1028548 |
| Q9JKF6 | Nectin-1 OS=Mus musculus OX=10090 GN=Nectin1 PE=1 SV=3 | 57 | 5 | 3 | 3 |  | |  | 3009760 |
| P07356 | Annexin A2 OS=Mus musculus OX=10090 GN=Anxa2 PE=1 SV=2 | 38.7 | 7 | 2 | 2 |  | |  | 302708 |
| Q8CAY6 | Acetyl-CoA acetyltransferase. cytosolic OS=Mus musculus OX=10090 GN=Acat2 PE=1 SV=2 | 41.3 | 11 | 3 | 2 |  | | 28608 | 471491 |
| O88783 | Coagulation factor V OS=Mus musculus OX=10090 GN=F5 PE=1 SV=1 | 247.1 | 2 | 3 | 3 | 62606 | |  | 145849 |
| Q8R5H1 | Ubiquitin carboxyl-terminal hydrolase 15 OS=Mus musculus OX=10090 GN=Usp15 PE=1 SV=1 | 112.3 | 3 | 4 | 3 |  | |  | 137715 |
| Q6P1B1 | Xaa-Pro aminopeptidase 1 OS=Mus musculus OX=10090 GN=Xpnpep1 PE=1 SV=1 | 69.5 | 4 | 3 | 2 |  | |  | 1807001 |
| Q9CWS0 | N(G).N(G)-dimethylarginine dimethylaminohydrolase 1 OS=Mus musculus OX=10090 GN=Ddah1 PE=1 SV=3 | 31.4 | 14 | 3 | 3 |  | |  | 3745174 |
| P30416 | Peptidyl-prolyl cis-trans isomerase FKBP4 OS=Mus musculus OX=10090 GN=Fkbp4 PE=1 SV=5 | 51.5 | 6 | 2 | 2 |  | |  | 619891 |
| Q3TTY5 | Keratin. type II cytoskeletal 2 epidermal OS=Mus musculus OX=10090 GN=Krt2 PE=1 SV=1 | 70.9 | 3 | 19 | 1 | 83236 | | 527570 | 503744 |
| Q9CWM4 | Prefoldin subunit 1 OS=Mus musculus OX=10090 GN=Pfdn1 PE=1 SV=1 | 14.2 | 23 | 4 | 3 |  | |  | 1009767 |
| Q9QZ08 | N-acetyl-D-glucosamine kinase OS=Mus musculus OX=10090 GN=Nagk PE=1 SV=3 | 37.2 | 9 | 2 | 2 |  | |  | 1752827 |
| P16056 | Hepatocyte growth factor receptor OS=Mus musculus OX=10090 GN=Met PE=1 SV=1 | 153.5 | 2 | 2 | 2 |  | |  | 468129 |
| Q923D2 | Flavin reductase (NADPH) OS=Mus musculus OX=10090 GN=Blvrb PE=1 SV=3 | 22.2 | 10 | 2 | 1 | 60856 | | 21321 | 157935 |
| Q9Z1T2 | Thrombospondin-4 OS=Mus musculus OX=10090 GN=Thbs4 PE=1 SV=1 | 106.3 | 3 | 8 | 2 | 177518 | | 425673 | 56894 |
| P08249 | Malate dehydrogenase. mitochondrial OS=Mus musculus OX=10090 GN=Mdh2 PE=1 SV=3 | 35.6 | 6 | 3 | 1 | 94783 | |  | 25941 |
| P29758 | Ornithine aminotransferase. mitochondrial OS=Mus musculus OX=10090 GN=Oat PE=1 SV=1 | 48.3 | 5 | 3 | 2 |  | |  | 1193032 |
| Q9QZF2 | Glypican-1 OS=Mus musculus OX=10090 GN=Gpc1 PE=1 SV=1 | 61.3 | 9 | 3 | 3 | 11926 | |  | 264016 |
| P34022 | Ran-specific GTPase-activating protein OS=Mus musculus OX=10090 GN=Ranbp1 PE=1 SV=2 | 23.6 | 11 | 5 | 2 | 5899920 | |  | 2057821 |
| O35685 | Nuclear migration protein nudC OS=Mus musculus OX=10090 GN=Nudc PE=1 SV=1 | 38.3 | 5 | 3 | 1 | 20397 | |  | 66414 |
| Q8BGQ7 | Alanine--tRNA ligase. cytoplasmic OS=Mus musculus OX=10090 GN=Aars PE=1 SV=1 | 106.8 | 2 | 1 | 1 |  | |  | 55180 |
| Q60864 | Stress-induced-phosphoprotein 1 OS=Mus musculus OX=10090 GN=Stip1 PE=1 SV=1 | 62.5 | 4 | 2 | 2 |  | |  | 356254 |
| Q8BK64 | Activator of 90 kDa heat shock protein ATPase homolog 1 OS=Mus musculus OX=10090 GN=Ahsa1 PE=1 SV=2 | 38.1 | 7 | 1 | 1 |  | |  |  |
| Q61739 | Integrin alpha-6 OS=Mus musculus OX=10090 GN=Itga6 PE=1 SV=3 | 122.1 | 2 | 3 | 2 |  | |  | 50469 |
| Q9JLJ2 | 4-trimethylaminobutyraldehyde dehydrogenase OS=Mus musculus OX=10090 GN=Aldh9a1 PE=1 SV=1 | 53.5 | 4 | 5 | 2 | 246232 | | 70474 | 1100757 |
| Q9Z2T6 | Keratin. type II cuticular Hb5 OS=Mus musculus OX=10090 GN=Krt85 PE=1 SV=2 | 55.7 | 5 | 4 | 2 | 7175675 | | 234305 |  |
| Q8VC97 | Beta-ureidopropionase OS=Mus musculus OX=10090 GN=Upb1 PE=1 SV=1 | 43.9 | 5 | 1 | 1 |  | |  | 1924591 |
| Q8BH61 | Coagulation factor XIII A chain OS=Mus musculus OX=10090 GN=F13a1 PE=1 SV=3 | 83.2 | 4 | 4 | 2 |  | |  | 586510 |
| Q3UM45 | Protein phosphatase 1 regulatory subunit 7 OS=Mus musculus OX=10090 GN=Ppp1r7 PE=1 SV=2 | 41.3 | 5 | 1 | 1 |  | |  |  |
| P62702 | 40S ribosomal protein S4. X isoform OS=Mus musculus OX=10090 GN=Rps4x PE=1 SV=2 | 29.6 | 7 | 3 | 2 |  | |  | 83286 |
| Q60675 | Laminin subunit alpha-2 OS=Mus musculus OX=10090 GN=Lama2 PE=1 SV=2 | 343.6 | 1 | 5 | 2 |  | | 15781 | 57826 |
| Q8R1K4 | 5-phosphohydroxy-L-lysine phospho-lyase OS=Mus musculus OX=10090 GN=Phykpl PE=1 SV=1 | 51.9 | 3 | 1 | 1 |  | |  | 321960 |
| Q62219 | Transforming growth factor beta-1-induced transcript 1 protein OS=Mus musculus OX=10090 GN=Tgfb1i1 PE=1 SV=2 | 50.1 | 5 | 5 | 2 | 5361 | | 16643 | 83435 |
| Q920A5 | Retinoid-inducible serine carboxypeptidase OS=Mus musculus OX=10090 GN=Scpep1 PE=1 SV=2 | 50.9 | 3 | 3 | 1 | 131452 | |  | 345388 |
| P00329 | Alcohol dehydrogenase 1 OS=Mus musculus OX=10090 GN=Adh1 PE=1 SV=2 | 39.7 | 5 | 8 | 2 | 222235 | |  | 4338555 |
| Q8CBR6 | Tsukushin OS=Mus musculus OX=10090 GN=Tsku PE=2 SV=2 | 38.3 | 4 | 4 | 1 | 24875 | | 21863 | 35097 |
| Q64475 | Histone H2B type 1-B OS=Mus musculus OX=10090 GN=Hist1h2bb PE=1 SV=3 | 13.9 | 12 | 1 | 1 |  | | 113347 |  |
| P35980 | 60S ribosomal protein L18 OS=Mus musculus OX=10090 GN=Rpl18 PE=1 SV=3 | 21.6 | 7 | 2 | 1 |  | |  | 47344 |
| O08638 | Myosin-11 OS=Mus musculus OX=10090 GN=Myh11 PE=1 SV=1 | 226.9 | 1 | 2 | 1 |  | |  | 100229 |
| P01630 | Ig kappa chain V-II region 7S34.1 OS=Mus musculus OX=10090 PE=1 SV=1 | 12.5 | 10 | 6 | 1 | 4227995 | | 3770341 | 2329479 |
| Q9Z2L6 | Multiple inositol polyphosphate phosphatase 1 OS=Mus musculus OX=10090 GN=Minpp1 PE=1 SV=3 | 54.5 | 3 | 3 | 1 |  | | 291332 | 615081 |
| P49312 | Heterogeneous nuclear ribonucleoprotein A1 OS=Mus musculus OX=10090 GN=Hnrnpa1 PE=1 SV=2 | 34.2 | 5 | 3 | 1 |  | |  | 293772 |
| P50516 | V-type proton ATPase catalytic subunit A OS=Mus musculus OX=10090 GN=Atp6v1a PE=1 SV=2 | 68.3 | 3 | 1 | 1 |  | |  | 272523 |
| Q501J6 | Probable ATP-dependent RNA helicase DDX17 OS=Mus musculus OX=10090 GN=Ddx17 PE=1 SV=1 | 72.4 | 4 | 3 | 2 |  | |  | 41194 |
| Q99KI0 | Aconitate hydratase. mitochondrial OS=Mus musculus OX=10090 GN=Aco2 PE=1 SV=1 | 85.4 | 5 | 2 | 2 |  | | 2060681 | 18542406 |
| Q9CZ30 | Obg-like ATPase 1 OS=Mus musculus OX=10090 GN=Ola1 PE=1 SV=1 | 44.7 | 4 | 1 | 1 | 64034 | |  |  |
| P18528 | Ig heavy chain V region 6.96 OS=Mus musculus OX=10090 PE=4 SV=1 | 11 | 19 | 2 | 1 |  | |  | 1210510 |
| P01869 | Ig gamma-1 chain C region. membrane-bound form OS=Mus musculus OX=10090 GN=Ighg1 PE=1 SV=2 | 43.4 | 11 | 37 | 2 | 232891731 | | 96779566 | 129013040 |
| P48774 | Glutathione S-transferase Mu 5 OS=Mus musculus OX=10090 GN=Gstm5 PE=1 SV=1 | 26.6 | 7 | 1 | 1 |  | |  |  |
| Q60605 | Myosin light polypeptide 6 OS=Mus musculus OX=10090 GN=Myl6 PE=1 SV=3 | 16.9 | 11 | 4 | 1 | 26834 | |  | 307771 |
| Q8C166 | Copine-1 OS=Mus musculus OX=10090 GN=Cpne1 PE=1 SV=1 | 58.8 | 3 | 4 | 2 | 27828 | |  | 243180 |
| Q9CQN4 | Sclerostin domain-containing protein 1 OS=Mus musculus OX=10090 GN=Sostdc1 PE=1 SV=1 | 23.2 | 7 | 2 | 1 |  | | 28931 | 696186 |
| E9Q557 | Desmoplakin OS=Mus musculus OX=10090 GN=Dsp PE=1 SV=1 | 332.7 | 0 | 1 | 1 |  | |  |  |
| Q3TNA1 | Xylulose kinase OS=Mus musculus OX=10090 GN=Xylb PE=1 SV=1 | 59.5 | 5 | 4 | 1 |  | | 242682 | 168754 |
| Q5FWK3 | Rho GTPase-activating protein 1 OS=Mus musculus OX=10090 GN=Arhgap1 PE=1 SV=1 | 50.4 | 3 | 1 | 1 |  | |  |  |
| P70695 | Fructose-1.6-bisphosphatase isozyme 2 OS=Mus musculus OX=10090 GN=Fbp2 PE=1 SV=2 | 36.9 | 3 | 4 | 1 | 40131 | | 9737 | 4556127 |
| P09581 | Macrophage colony-stimulating factor 1 receptor OS=Mus musculus OX=10090 GN=Csf1r PE=1 SV=3 | 109.1 | 1 | 3 | 1 | 838323 | | 817704 | 634901 |
| P06909 | Complement factor H OS=Mus musculus OX=10090 GN=Cfh PE=1 SV=2 | 139 | 2 | 7 | 2 | 5112849 | | 1902671 | 4272423 |
| P62245 | 40S ribosomal protein S15a OS=Mus musculus OX=10090 GN=Rps15a PE=1 SV=2 | 14.8 | 13 | 3 | 2 |  | |  | 170689 |
| Q99L13 | 3-hydroxyisobutyrate dehydrogenase. mitochondrial OS=Mus musculus OX=10090 GN=Hibadh PE=1 SV=1 | 35.4 | 5 | 1 | 1 |  | |  | 241383 |
| P01631 | Ig kappa chain V-II region 26-10 OS=Mus musculus OX=10090 PE=1 SV=1 | 12.3 | 12 | 22 | 1 | 169640713 | | 97837396 | 93734668 |
| Q61233 | Plastin-2 OS=Mus musculus OX=10090 GN=Lcp1 PE=1 SV=4 | 70.1 | 5 | 4 | 1 |  | |  |  |
| Q6NVD0 | FRAS1-related extracellular matrix protein 2 OS=Mus musculus OX=10090 GN=Frem2 PE=1 SV=2 | 350.4 | 0 | 1 | 1 |  | |  |  |
| Q70E20 | Sushi. nidogen and EGF-like domain-containing protein 1 OS=Mus musculus OX=10090 GN=Sned1 PE=2 SV=2 | 151.5 | 1 | 2 | 2 |  | |  | 175455 |
| P14148 | 60S ribosomal protein L7 OS=Mus musculus OX=10090 GN=Rpl7 PE=1 SV=2 | 31.4 | 7 | 9 | 1 | 19553 | | 2663 | 139746 |
| Q9WUH7 | Semaphorin-4G OS=Mus musculus OX=10090 GN=Sema4g PE=1 SV=1 | 92.3 | 6 | 2 | 2 |  | |  |  |
| P61979 | Heterogeneous nuclear ribonucleoprotein K OS=Mus musculus OX=10090 GN=Hnrnpk PE=1 SV=1 | 50.9 | 3 | 2 | 1 | 11292 | |  |  |
| Q9D892 | Inosine triphosphate pyrophosphatase OS=Mus musculus OX=10090 GN=Itpa PE=1 SV=2 | 21.9 | 9 | 2 | 1 |  | |  | 960329 |
| P63038 | 60 kDa heat shock protein. mitochondrial OS=Mus musculus OX=10090 GN=Hspd1 PE=1 SV=1 | 60.9 | 4 | 4 | 1 |  | |  | 49050 |
| Q922R8 | Protein disulfide-isomerase A6 OS=Mus musculus OX=10090 GN=Pdia6 PE=1 SV=3 | 48.1 | 5 | 2 | 1 |  | |  |  |
| O70325 | Phospholipid hydroperoxide glutathione peroxidase OS=Mus musculus OX=10090 GN=Gpx4 PE=1 SV=4 | 22.2 | 13 | 3 | 2 | 72947 | |  | 353471 |
| O35522 | Proteasome subunit beta type-9 OS=Mus musculus bactrianus OX=35531 GN=Psmb9 PE=1 SV=1 | 23.4 | 7 | 3 | 1 | 46046 | |  | 72589 |
| Q9DBV4 | Matrix remodeling-associated protein 8 OS=Mus musculus OX=10090 GN=Mxra8 PE=1 SV=1 | 49.7 | 3 | 1 | 1 |  | |  | 540855 |
| P17897 | Lysozyme C-1 OS=Mus musculus OX=10090 GN=Lyz1 PE=1 SV=1 | 16.8 | 8 | 1 | 1 |  | |  | 136728 |
| Q99L20 | Glutathione S-transferase theta-3 OS=Mus musculus OX=10090 GN=Gstt3 PE=1 SV=1 | 27.4 | 6 | 5 | 2 | 31466 | |  | 1910278 |
| Q4KML4 | Costars family protein ABRACL OS=Mus musculus OX=10090 GN=Abracl PE=1 SV=1 | 9 | 16 | 3 | 1 |  | |  | 124054 |
| Q9WV02 | RNA-binding motif protein. X chromosome OS=Mus musculus OX=10090 GN=Rbmx PE=1 SV=1 | 42.3 | 4 | 2 | 1 |  | |  | 170190 |
| P01636 | Ig kappa chain V-V region MOPC 149 OS=Mus musculus OX=10090 PE=1 SV=1 | 12 | 12 | 3 | 1 | 8413762 | | 3793634 | 5169878 |
| P51655 | Glypican-4 OS=Mus musculus OX=10090 GN=Gpc4 PE=1 SV=2 | 62.5 | 3 | 1 | 1 |  | |  | 10530 |
| P62889 | 60S ribosomal protein L30 OS=Mus musculus OX=10090 GN=Rpl30 PE=1 SV=2 | 12.8 | 27 | 3 | 2 | 326936 | |  | 117500 |
| Q7TSK7 | ADAMTS-like protein 2 OS=Mus musculus OX=10090 GN=Adamtsl2 PE=2 SV=1 | 105.6 | 2 | 2 | 1 |  | |  | 4768 |
| P58019 | CD59B glycoprotein OS=Mus musculus OX=10090 GN=Cd59b PE=2 SV=2 | 14.2 | 9 | 2 | 1 |  | |  |  |
| P18525 | Ig heavy chain V region 5-84 OS=Mus musculus OX=10090 PE=1 SV=1 | 12.9 | 14 | 1 | 1 | 88462 | |  |  |
| Q8K1I3 | Secreted phosphoprotein 24 OS=Mus musculus OX=10090 GN=Spp2 PE=1 SV=2 | 23.1 | 11 | 4 | 2 | 72166 | | 148757 | 185909 |
| P14131 | 40S ribosomal protein S16 OS=Mus musculus OX=10090 GN=Rps16 PE=1 SV=4 | 16.4 | 15 | 4 | 2 | 124302 | | 17223 | 61807 |
| P20826 | Kit ligand OS=Mus musculus OX=10090 GN=Kitlg PE=1 SV=1 | 30.6 | 6 | 198 | 1 | 64071125 | | 82827889 | 51458666 |
| O89103 | Complement component C1q receptor OS=Mus musculus OX=10090 GN=Cd93 PE=1 SV=1 | 69.3 | 2 | 4 | 1 | 233500 | | 162514 | 1430636 |
| Q07563 | Collagen alpha-1(XVII) chain OS=Mus musculus OX=10090 GN=Col17a1 PE=1 SV=3 | 147.9 | 1 | 1 | 1 |  | |  |  |
| Q99K41 | EMILIN-1 OS=Mus musculus OX=10090 GN=Emilin1 PE=1 SV=1 | 107.5 | 3 | 2 | 2 |  | |  | 84400 |
| P18524 | Ig heavy chain V region RF OS=Mus musculus OX=10090 PE=1 SV=1 | 12.9 | 9 | 8 | 1 | 5604598 | | 2870215 | 1447263 |
| P48678 | Prelamin-A/C OS=Mus musculus OX=10090 GN=Lmna PE=1 SV=2 | 74.2 | 2 | 1 | 1 | 20740 | |  |  |
| Q9EQ80 | NIF3-like protein 1 OS=Mus musculus OX=10090 GN=Nif3l1 PE=1 SV=4 | 41.7 | 3 | 2 | 1 | 311717 | | 189983 | 611237 |
| P01635 | Ig kappa chain V-V region K2 (Fragment) OS=Mus musculus OX=10090 PE=1 SV=1 | 12.6 | 11 | 18 | 1 | 68793861 | | 47299753 | 38743461 |
| P04919 | Band 3 anion transport protein OS=Mus musculus OX=10090 GN=Slc4a1 PE=1 SV=1 | 103.1 | 2 | 4 | 2 | 183589 | | 249281 | 861477 |
| P62301 | 40S ribosomal protein S13 OS=Mus musculus OX=10090 GN=Rps13 PE=1 SV=2 | 17.2 | 11 | 3 | 2 | 30642 | |  | 1663115 |
| P62855 | 40S ribosomal protein S26 OS=Mus musculus OX=10090 GN=Rps26 PE=1 SV=3 | 13 | 21 | 3 | 2 |  | |  | 238067 |
| P06151 | L-lactate dehydrogenase A chain OS=Mus musculus OX=10090 GN=Ldha PE=1 SV=3 | 36.5 | 6 | 5 | 1 | 19128 | | 20509 | 75342 |
| Q8BTY1 | Kynurenine--oxoglutarate transaminase 1 OS=Mus musculus OX=10090 GN=Kyat1 PE=1 SV=1 | 47.5 | 6 | 2 | 1 |  | |  | 108423 |
| Q9QXC1 | Fetuin-B OS=Mus musculus OX=10090 GN=Fetub PE=1 SV=1 | 42.7 | 3 | 4 | 2 | 231833 | | 873495 | 1715410 |
| Q61527 | Receptor tyrosine-protein kinase erbB-4 OS=Mus musculus OX=10090 GN=Erbb4 PE=1 SV=5 | 146.8 | 2 | 1 | 1 |  | |  |  |
| P62897 | Cytochrome c. somatic OS=Mus musculus OX=10090 GN=Cycs PE=1 SV=2 | 11.6 | 10 | 2 | 1 |  | |  |  |
| P46467 | Vacuolar protein sorting-associated protein 4B OS=Mus musculus OX=10090 GN=Vps4b PE=1 SV=2 | 49.4 | 5 | 1 | 1 |  | |  | 64237 |
| Q9Z1N5 | Spliceosome RNA helicase Ddx39b OS=Mus musculus OX=10090 GN=Ddx39b PE=1 SV=1 | 49 | 2 | 1 | 1 |  | |  | 521047 |
| Q91Y47 | Coagulation factor XI OS=Mus musculus OX=10090 GN=F11 PE=2 SV=2 | 69.7 | 2 | 3 | 1 | 719901 | | 167442 | 202723 |
| P35918 | Vascular endothelial growth factor receptor 2 OS=Mus musculus OX=10090 GN=Kdr PE=1 SV=1 | 152.4 | 1 | 1 | 1 |  | | 62910 | 215827 |
| P54731 | FAS-associated factor 1 OS=Mus musculus OX=10090 GN=Faf1 PE=1 SV=2 | 73.8 | 2 | 1 | 1 |  | |  | 23239 |
| Q80W21 | Glutathione S-transferase Mu 7 OS=Mus musculus OX=10090 GN=Gstm7 PE=1 SV=1 | 25.7 | 6 | 2 | 1 |  | |  |  |
| Q8BG32 | 26S proteasome non-ATPase regulatory subunit 11 OS=Mus musculus OX=10090 GN=Psmd11 PE=1 SV=3 | 47.4 | 3 | 3 | 1 |  | |  | 20015 |
| P56480 | ATP synthase subunit beta. mitochondrial OS=Mus musculus OX=10090 GN=Atp5f1b PE=1 SV=2 | 56.3 | 6 | 4 | 2 | 1693752 | | 282082 | 7457004 |
| P01629 | Ig kappa chain V-II region 2S1.3 OS=Mus musculus OX=10090 PE=1 SV=1 | 12.2 | 12 | 8 | 1 | 3875988 | | 1264574 | 1294561 |
| Q06335 | Amyloid-like protein 2 OS=Mus musculus OX=10090 GN=Aplp2 PE=1 SV=4 | 80.4 | 3 | 1 | 1 |  | |  | 24021 |
| P30115 | Glutathione S-transferase A3 OS=Mus musculus OX=10090 GN=Gsta3 PE=1 SV=2 | 25.3 | 7 | 5 | 1 |  | |  | 746367 |
| P01791 | Ig heavy chain V region HPCM6 OS=Mus musculus OX=10090 PE=1 SV=1 | 13.9 | 9 | 1 | 1 | 2336569 | |  |  |
| P97449 | Aminopeptidase N OS=Mus musculus OX=10090 GN=Anpep PE=1 SV=4 | 109.6 | 1 | 1 | 1 |  | | 86652 | 181913 |
| Q3SXD3 | HD domain-containing protein 2 OS=Mus musculus OX=10090 GN=Hddc2 PE=1 SV=1 | 22.7 | 9 | 1 | 1 | 333581 | |  |  |
| P29699 | Alpha-2-HS-glycoprotein OS=Mus musculus OX=10090 GN=Ahsg PE=1 SV=1 | 37.3 | 6 | 1 | 1 |  | |  |  |
| Q7TSV4 | Phosphoglucomutase-2 OS=Mus musculus OX=10090 GN=Pgm2 PE=1 SV=1 | 68.7 | 3 | 3 | 1 | 413990 | |  | 303148 |
| Q8CIG8 | Protein arginine N-methyltransferase 5 OS=Mus musculus OX=10090 GN=Prmt5 PE=1 SV=3 | 72.6 | 2 | 1 | 1 |  | |  |  |
| Q61990 | Poly(rC)-binding protein 2 OS=Mus musculus OX=10090 GN=Pcbp2 PE=1 SV=1 | 38.2 | 3 | 1 | 1 |  | |  |  |
| P50543 | Protein S100-A11 OS=Mus musculus OX=10090 GN=S100a11 PE=1 SV=1 | 11.1 | 11 | 1 | 1 |  | | 16561 |  |
| Q9R0N0 | Galactokinase OS=Mus musculus OX=10090 GN=Galk1 PE=1 SV=2 | 42.3 | 3 | 2 | 1 | 42500 | |  |  |
| Q8R2K1 | Fucose mutarotase OS=Mus musculus OX=10090 GN=Fuom PE=1 SV=1 | 16.8 | 16 | 2 | 1 |  | | 24923 | 358240 |
| P04202 | Transforming growth factor beta-1 proprotein OS=Mus musculus OX=10090 GN=Tgfb1 PE=1 SV=1 | 44.3 | 3 | 1 | 1 |  | |  | 77231 |
| Q7TQI3 | Ubiquitin thioesterase OTUB1 OS=Mus musculus OX=10090 GN=Otub1 PE=1 SV=2 | 31.3 | 7 | 1 | 1 |  | | 20516 | 258517 |
| P61205 | ADP-ribosylation factor 3 OS=Mus musculus OX=10090 GN=Arf3 PE=2 SV=2 | 20.6 | 6 | 1 | 1 |  | |  |  |
| Q9D0J8 | Parathymosin OS=Mus musculus OX=10090 GN=Ptms PE=1 SV=3 | 11.4 | 11 | 3 | 1 | 199634 | |  | 20375795 |
| O09131 | Glutathione S-transferase omega-1 OS=Mus musculus OX=10090 GN=Gsto1 PE=1 SV=2 | 27.5 | 10 | 6 | 2 | 1223237 | | 89058 | 5460776 |
| O08795 | Glucosidase 2 subunit beta OS=Mus musculus OX=10090 GN=Prkcsh PE=1 SV=1 | 58.8 | 2 | 3 | 1 | 406242 | |  | 418392 |
| E9PVD3 | Protocadherin-16 OS=Mus musculus OX=10090 GN=Dchs1 PE=1 SV=1 | 346.2 | 0 | 1 | 1 |  | |  | 73767 |
| Q60866 | Phosphotriesterase-related protein OS=Mus musculus OX=10090 GN=Pter PE=1 SV=1 | 39.2 | 3 | 2 | 1 |  | |  | 869919 |
| P56959 | RNA-binding protein FUS OS=Mus musculus OX=10090 GN=Fus PE=1 SV=1 | 52.6 | 3 | 3 | 1 | 1410694 | | 1821649 | 2154676 |
| P62717 | 60S ribosomal protein L18a OS=Mus musculus OX=10090 GN=Rpl18a PE=1 SV=1 | 20.7 | 7 | 1 | 1 |  | |  |  |
| Q8VCN5 | Cystathionine gamma-lyase OS=Mus musculus OX=10090 GN=Cth PE=1 SV=1 | 43.5 | 3 | 2 | 1 |  | |  | 235090 |
| P01800 | Ig heavy chain V-III region T957 OS=Mus musculus OX=10090 PE=1 SV=2 | 12.7 | 8 | 5 | 1 | 4369922 | | 1473920 | 2459169 |
| Q91W89 | Alpha-mannosidase 2C1 OS=Mus musculus OX=10090 GN=Man2c1 PE=1 SV=1 | 115.6 | 1 | 1 | 1 | 34307 | |  |  |
| P70195 | Proteasome subunit beta type-7 OS=Mus musculus OX=10090 GN=Psmb7 PE=1 SV=1 | 29.9 | 4 | 5 | 1 | 991574 | |  | 988870 |
| P01642 | Ig kappa chain V-V region L7 (Fragment) OS=Mus musculus OX=10090 GN=Gm10881 PE=1 SV=1 | 12.6 | 10 | 5 | 1 | 2189523 | | 1077300 | 755689 |
| Q9JLI6 | Selenocysteine lyase OS=Mus musculus OX=10090 GN=Scly PE=1 SV=1 | 47.1 | 3 | 1 | 1 |  | |  |  |
| P15379 | CD44 antigen OS=Mus musculus OX=10090 GN=Cd44 PE=1 SV=3 | 85.6 | 1 | 2 | 1 | 151328 | |  |  |
| Q7TMD7 | Desmoglein-4 OS=Mus musculus OX=10090 GN=Dsg4 PE=1 SV=1 | 114.4 | 2 | 2 | 1 |  | | 53359 |  |
| P08122 | Collagen alpha-2(IV) chain OS=Mus musculus OX=10090 GN=Col4a2 PE=1 SV=4 | 167.2 | 1 | 2 | 1 | 336541 | | 37321 | 21587 |
| A6H5Y3 | Methionine synthase OS=Mus musculus OX=10090 GN=Mtr PE=1 SV=1 | 139 | 2 | 1 | 1 |  | |  |  |
| Q9D2V7 | Coronin-7 OS=Mus musculus OX=10090 GN=Coro7 PE=1 SV=2 | 100.7 | 3 | 1 | 1 |  | |  | 83665 |
| Q9WVM8 | Kynurenine/alpha-aminoadipate aminotransferase. mitochondrial OS=Mus musculus OX=10090 GN=Aadat PE=1 SV=1 | 47.6 | 2 | 1 | 1 |  | |  | 867033 |
| Q64514 | Tripeptidyl-peptidase 2 OS=Mus musculus OX=10090 GN=Tpp2 PE=1 SV=3 | 139.8 | 1 | 1 | 1 | 252172 | |  | 126665 |
| P62962 | Profilin-1 OS=Mus musculus OX=10090 GN=Pfn1 PE=1 SV=2 | 14.9 | 10 | 2 | 1 |  | |  |  |
| Q9WU78 | Programmed cell death 6-interacting protein OS=Mus musculus OX=10090 GN=Pdcd6ip PE=1 SV=3 | 96 | 1 | 1 | 1 |  | |  | 238879 |
| Q8BJD1 | Inter-alpha-trypsin inhibitor heavy chain H5 OS=Mus musculus OX=10090 GN=Itih5 PE=1 SV=1 | 106.7 | 1 | 2 | 1 |  | | 291858 | 65718 |
| Q9CR86 | Calcium-regulated heat stable protein 1 OS=Mus musculus OX=10090 GN=Carhsp1 PE=1 SV=1 | 16.1 | 11 | 3 | 1 | 145649 | |  | 708879 |
| P20152 | Vimentin OS=Mus musculus OX=10090 GN=Vim PE=1 SV=3 | 53.7 | 2 | 1 | 1 |  | |  |  |
| P01741 | Ig heavy chain V region OS=Mus musculus OX=10090 PE=1 SV=1 | 12.5 | 11 | 1 | 1 | 395170 | | 371434 |  |
| P56400 | Platelet glycoprotein Ib beta chain OS=Mus musculus OX=10090 GN=Gp1bb PE=1 SV=1 | 21.7 | 5 | 2 | 1 | 66825 | |  | 136931 |
| P97314 | Cysteine and glycine-rich protein 2 OS=Mus musculus OX=10090 GN=Csrp2 PE=1 SV=3 | 20.9 | 8 | 2 | 1 |  | |  | 43536 |
| Q8VCG4 | Complement component C8 gamma chain OS=Mus musculus OX=10090 GN=C8g PE=1 SV=1 | 22.5 | 5 | 4 | 1 | 823289 | | 109371 | 864201 |
| Q9QUM0 | Integrin alpha-IIb OS=Mus musculus OX=10090 GN=Itga2b PE=1 SV=2 | 112.6 | 1 | 3 | 1 | 121657 | | 10434 | 33625 |
| Q60590 | Alpha-1-acid glycoprotein 1 OS=Mus musculus OX=10090 GN=Orm1 PE=1 SV=1 | 23.9 | 4 | 6 | 1 | 32026894 | | 14486414 | 26516250 |
| P84750 | Ig kappa chain V region Mem5 (Fragment) OS=Mus musculus OX=10090 PE=1 SV=1 | 13.2 | 8 | 3 | 1 | 4984876 | | 3194472 | 4320016 |
| P05017 | Insulin-like growth factor I OS=Mus musculus OX=10090 GN=Igf1 PE=1 SV=2 | 17.1 | 10 | 1 | 1 | 48969 | | 76131 | 226861 |
| Q9QYK5 | Heparan-sulfate 6-O-sulfotransferase 1 OS=Mus musculus OX=10090 GN=Hs6st1 PE=1 SV=4 | 48.3 | 3 | 2 | 1 |  | |  | 595990 |
| O08808 | Protein diaphanous homolog 1 OS=Mus musculus OX=10090 GN=Diaph1 PE=1 SV=1 | 139.3 | 2 | 2 | 1 |  | |  | 72425 |
| P12246 | Serum amyloid P-component OS=Mus musculus OX=10090 GN=Apcs PE=1 SV=2 | 26.2 | 4 | 6 | 1 | 25444949 | | 24837717 | 20261728 |
| Q91YE6 | Importin-9 OS=Mus musculus OX=10090 GN=Ipo9 PE=1 SV=3 | 116 | 2 | 1 | 1 |  | |  | 21852 |
| Q9Z2V4 | Phosphoenolpyruvate carboxykinase. cytosolic [GTP] OS=Mus musculus OX=10090 GN=Pck1 PE=1 SV=1 | 69.3 | 1 | 2 | 1 |  | |  | 351933 |
| Q60598 | Src substrate cortactin OS=Mus musculus OX=10090 GN=Cttn PE=1 SV=2 | 61.2 | 2 | 1 | 1 |  | |  | 200205 |
| Q01339 | Beta-2-glycoprotein 1 OS=Mus musculus OX=10090 GN=Apoh PE=1 SV=1 | 38.6 | 3 | 4 | 1 | 26239873 | | 24939594 | 24610687 |
| P01657 | Ig kappa chain V-III region PC 2413 OS=Mus musculus OX=10090 PE=1 SV=1 | 11.9 | 8 | 5 | 1 | 23993388 | | 12046153 | 10332566 |
| Q64323 | N-acetylglucosaminyl-phosphatidylinositol biosynthetic protein OS=Mus musculus OX=10090 GN=Piga PE=2 SV=1 | 54.4 | 1 | 6 | 1 | 7482220 | | 5804141 | 17345013 |
| P08030 | Adenine phosphoribosyltransferase OS=Mus musculus OX=10090 GN=Aprt PE=1 SV=2 | 19.7 | 11 | 1 | 1 |  | |  |  |
| Q9CZ44 | NSFL1 cofactor p47 OS=Mus musculus OX=10090 GN=Nsfl1c PE=1 SV=1 | 40.7 | 4 | 1 | 1 |  | |  |  |
| Q02105 | Complement C1q subcomponent subunit C OS=Mus musculus OX=10090 GN=C1qc PE=1 SV=2 | 26 | 4 | 1 | 1 | 165147 | | 41167 | 132235 |
| Q71KU9 | Fibrinogen-like protein 1 OS=Mus musculus OX=10090 GN=Fgl1 PE=1 SV=2 | 36.4 | 3 | 2 | 1 |  | | 77143 | 50571 |
| Q06806 | Tyrosine-protein kinase receptor Tie-1 OS=Mus musculus OX=10090 GN=Tie1 PE=1 SV=3 | 124.5 | 2 | 1 | 1 |  | |  |  |
| P01887 | Beta-2-microglobulin OS=Mus musculus OX=10090 GN=B2m PE=1 SV=2 | 13.8 | 8 | 1 | 1 | 3622052 | |  | 100309 |
| O88456 | Calpain small subunit 1 OS=Mus musculus OX=10090 GN=Capns1 PE=1 SV=1 | 28.4 | 4 | 3 | 1 | 2063835 | | 402930 | 1197581 |
| P33587 | Vitamin K-dependent protein C OS=Mus musculus OX=10090 GN=Proc PE=1 SV=2 | 51.8 | 2 | 1 | 1 |  | |  | 193306 |
| Q8BG07 | Phospholipase D4 OS=Mus musculus OX=10090 GN=Pld4 PE=1 SV=1 | 56.1 | 1 | 1 | 1 | 714202 | |  |  |
| P61759 | Prefoldin subunit 3 OS=Mus musculus OX=10090 GN=Vbp1 PE=1 SV=2 | 22.4 | 5 | 1 | 1 |  | |  | 116687 |
| P70697 | Uroporphyrinogen decarboxylase OS=Mus musculus OX=10090 GN=Urod PE=1 SV=2 | 40.7 | 3 | 1 | 1 |  | |  |  |
| P28474 | Alcohol dehydrogenase class-3 OS=Mus musculus OX=10090 GN=Adh5 PE=1 SV=3 | 39.5 | 7 | 2 | 1 |  | |  | 1007101 |
| Q9CWK8 | Sorting nexin-2 OS=Mus musculus OX=10090 GN=Snx2 PE=1 SV=2 | 58.4 | 2 | 1 | 1 |  | |  | 88412 |
| P84244 | Histone H3.3 OS=Mus musculus OX=10090 GN=H3f3a PE=1 SV=2 | 15.3 | 5 | 6 | 1 | 215538 | | 566762 | 3249001 |
| Q8BKC5 | Importin-5 OS=Mus musculus OX=10090 GN=Ipo5 PE=1 SV=3 | 123.5 | 2 | 1 | 1 | 20432 | |  | 50793 |
| P03958 | Adenosine deaminase OS=Mus musculus OX=10090 GN=Ada PE=1 SV=3 | 40 | 3 | 1 | 1 | 190822 | | 353393 | 751114 |
| Q8C1A5 | Thimet oligopeptidase OS=Mus musculus OX=10090 GN=Thop1 PE=1 SV=1 | 78 | 1 | 1 | 1 |  | |  | 601135 |
| P09055 | Integrin beta-1 OS=Mus musculus OX=10090 GN=Itgb1 PE=1 SV=1 | 88.2 | 2 | 1 | 1 |  | |  |  |
| Q8BHC0 | Lymphatic vessel endothelial hyaluronic acid receptor 1 OS=Mus musculus OX=10090 GN=Lyve1 PE=1 SV=1 | 34.6 | 3 | 4 | 1 | 1022452 | | 220550 | 1374981 |
| Q03958 | Prefoldin subunit 6 OS=Mus musculus OX=10090 GN=Pfdn6 PE=1 SV=1 | 14.4 | 6 | 1 | 1 |  | |  | 791404 |
| Q9WVJ2 | 26S proteasome non-ATPase regulatory subunit 13 OS=Mus musculus OX=10090 GN=Psmd13 PE=1 SV=1 | 42.8 | 3 | 1 | 1 | 148974 | |  |  |
| Q8K010 | 5-oxoprolinase OS=Mus musculus OX=10090 GN=Oplah PE=1 SV=1 | 137.5 | 1 | 1 | 1 |  | |  | 59687 |
| Q3V1G4 | Olfactomedin-like protein 2B OS=Mus musculus OX=10090 GN=Olfml2b PE=1 SV=2 | 83.5 | 1 | 1 | 1 |  | |  |  |
| Q69Z23 | Dynein heavy chain 17. axonemal OS=Mus musculus OX=10090 GN=Dnah17 PE=1 SV=2 | 511.3 | 0 | 23 | 1 | 25569023 | | 24154816 | 35618996 |
| Q91YR9 | Prostaglandin reductase 1 OS=Mus musculus OX=10090 GN=Ptgr1 PE=1 SV=2 | 35.5 | 3 | 1 | 1 |  | |  | 836353 |
| Q01149 | Collagen alpha-2(I) chain OS=Mus musculus OX=10090 GN=Col1a2 PE=1 SV=2 | 129.5 | 1 | 1 | 1 |  | | 800233 |  |
| P11627 | Neural cell adhesion molecule L1 OS=Mus musculus OX=10090 GN=L1cam PE=1 SV=1 | 140.9 | 1 | 2 | 1 |  | |  |  |
| Q9JHU9 | Inositol-3-phosphate synthase 1 OS=Mus musculus OX=10090 GN=Isyna1 PE=1 SV=1 | 60.9 | 1 | 1 | 1 | 63643 | | 19875 |  |
| Q8C0E2 | Vacuolar protein sorting-associated protein 26B OS=Mus musculus OX=10090 GN=Vps26b PE=1 SV=1 | 39.1 | 5 | 1 | 1 |  | |  |  |
| P0DP26 | Calmodulin-1 OS=Mus musculus OX=10090 GN=Calm1 PE=1 SV=1 | 16.8 | 11 | 1 | 1 |  | | 42621 | 42456 |
| Q62415 | Apoptosis-stimulating of p53 protein 1 OS=Mus musculus OX=10090 GN=Ppp1r13b PE=1 SV=2 | 119.1 | 1 | 1 | 1 | 57263 | |  |  |
| O54833 | Casein kinase II subunit alpha' OS=Mus musculus OX=10090 GN=Csnk2a2 PE=1 SV=1 | 41.2 | 3 | 1 | 1 |  | |  | 91919 |
| Q5XPI3 | E3 ubiquitin-protein ligase RNF123 OS=Mus musculus OX=10090 GN=Rnf123 PE=1 SV=1 | 148.6 | 2 | 1 | 1 | 63414 | | 25555 | 152976 |
| Q9Z1R3 | Apolipoprotein M OS=Mus musculus OX=10090 GN=Apom PE=1 SV=1 | 21.3 | 4 | 3 | 1 | 1124095 | |  | 287283 |
| Q60847 | Collagen alpha-1(XII) chain OS=Mus musculus OX=10090 GN=Col12a1 PE=2 SV=3 | 340 | 0 | 1 | 1 | 8135 | |  | 43878 |
| P11859 | Angiotensinogen OS=Mus musculus OX=10090 GN=Agt PE=1 SV=1 | 52 | 2 | 9 | 1 | 14630460 | | 21089796 | 15993891 |
| P03987 | Ig gamma-3 chain C region OS=Mus musculus OX=10090 PE=1 SV=2 | 43.9 | 3 | 6 | 1 | 52210199 | | 62089016 | 53000404 |
| P11680 | Properdin OS=Mus musculus OX=10090 GN=Cfp PE=2 SV=2 | 50.3 | 2 | 10 | 1 | 4870119 | | 5627892 | 7761821 |
| Q3U133 | Zinc finger protein 746 OS=Mus musculus OX=10090 GN=Znf746 PE=1 SV=3 | 69.8 | 1 | 2 | 1 |  | |  | 204449 |
| O35887 | Calumenin OS=Mus musculus OX=10090 GN=Calu PE=1 SV=1 | 37 | 5 | 1 | 1 |  | |  | 379885 |
| P97807 | Fumarate hydratase. mitochondrial OS=Mus musculus OX=10090 GN=Fh PE=1 SV=3 | 54.3 | 2 | 1 | 1 |  | |  | 856896 |
| Q9R171 | Cerebellin-1 OS=Mus musculus OX=10090 GN=Cbln1 PE=1 SV=1 | 21.1 | 4 | 1 | 1 | 223651 | | 37036 | 195141 |
| Q99LX0 | Protein/nucleic acid deglycase DJ-1 OS=Mus musculus OX=10090 GN=Park7 PE=1 SV=1 | 20 | 4 | 1 | 1 |  | |  | 461901 |
| Q9JII5 | DAZ-associated protein 1 OS=Mus musculus OX=10090 GN=Dazap1 PE=1 SV=2 | 43.2 | 4 | 2 | 1 | 179493 | |  | 143908 |
| Q6A078 | Centrosomal protein of 290 kDa OS=Mus musculus OX=10090 GN=Cep290 PE=1 SV=2 | 288.9 | 1 | 1 | 1 |  | |  | 611622 |
| P50396 | Rab GDP dissociation inhibitor alpha OS=Mus musculus OX=10090 GN=Gdi1 PE=1 SV=3 | 50.5 | 4 | 1 | 1 |  | |  |  |
| Q64318 | Zinc finger E-box-binding homeobox 1 OS=Mus musculus OX=10090 GN=Zeb1 PE=1 SV=1 | 122.4 | 1 | 1 | 1 | 128751 | | 188024 | 405754 |
| Q99LD4 | COP9 signalosome complex subunit 1 OS=Mus musculus OX=10090 GN=Gps1 PE=1 SV=1 | 53.4 | 2 | 1 | 1 |  | |  | 53165 |
| Q9R008 | Mevalonate kinase OS=Mus musculus OX=10090 GN=Mvk PE=1 SV=1 | 41.9 | 2 | 15 | 1 | 2682395 | | 5534544 | 10909956 |
| A2AKB4 | FERM and PDZ domain-containing protein 1 OS=Mus musculus OX=10090 GN=Frmpd1 PE=1 SV=1 | 169.1 | 1 | 1 | 1 | 47938 | | 25541 | 51144 |
| D3YZI9 | PiggyBac transposable element-derived protein 5 OS=Mus musculus OX=10090 GN=Pgbd5 PE=1 SV=1 | 58.3 | 2 | 4 | 1 | 1660549 | | 2748183 | 3848728 |
| Q6NSR8 | Probable aminopeptidase NPEPL1 OS=Mus musculus OX=10090 GN=Npepl1 PE=1 SV=1 | 55.9 | 2 | 1 | 1 | 4461830 | |  |  |
| A2ASS6 | Titin OS=Mus musculus OX=10090 GN=Ttn PE=1 SV=1 | 3904.1 | 0 | 4 | 1 |  | |  |  |
| P06328 | Ig heavy chain V region 1-72 OS=Mus musculus OX=10090 GN=Ighv1-72 PE=1 SV=2 | 12.9 | 6 | 3 | 1 | 1692754 | | 997284 | 1169651 |
| A3KGB4 | TBC1 domain family member 8B OS=Mus musculus OX=10090 GN=Tbc1d8b PE=1 SV=1 | 127.8 | 1 | 1 | 1 |  | |  |  |
| P35951 | Low-density lipoprotein receptor OS=Mus musculus OX=10090 GN=Ldlr PE=1 SV=2 | 94.9 | 2 | 1 | 1 | 7221 | | 39420 | 14280935 |
| Q99M74 | Keratin. type II cuticular Hb2 OS=Mus musculus OX=10090 GN=Krt82 PE=1 SV=2 | 57.1 | 2 | 3 | 1 | 4654568 | | 1168672 | 10779596 |
| Q9QZ88 | Vacuolar protein sorting-associated protein 29 OS=Mus musculus OX=10090 GN=Vps29 PE=1 SV=1 | 20.5 | 5 | 1 | 1 |  | |  | 108082 |
| Q62356 | Follistatin-related protein 1 OS=Mus musculus OX=10090 GN=Fstl1 PE=1 SV=2 | 34.5 | 5 | 1 | 1 |  | |  | 38639 |
| E9Q1U1 | Coiled-coil domain-containing protein 171 OS=Mus musculus OX=10090 GN=Ccdc171 PE=2 SV=1 | 152.2 | 1 | 1 | 1 | 335159 | | 404310 |  |
| Q62348 | Translin OS=Mus musculus OX=10090 GN=Tsn PE=1 SV=1 | 26.2 | 4 | 1 | 1 |  | |  | 163214 |
| P50294 | Arylamine N-acetyltransferase 1 OS=Mus musculus OX=10090 GN=Nat1 PE=1 SV=1 | 33.7 | 5 | 1 | 1 |  | |  |  |
| P01831 | Thy-1 membrane glycoprotein OS=Mus musculus OX=10090 GN=Thy1 PE=1 SV=1 | 18.1 | 9 | 2 | 1 | 67909 | | 101371 | 498003 |
| Q3UMC0 | ATPase family protein 2 homolog OS=Mus musculus OX=10090 GN=Spata5 PE=1 SV=2 | 97.2 | 2 | 1 | 1 | 70094 | | 50923 | 113649 |
| Q9DCG6 | Phenazine biosynthesis-like domain-containing protein 1 OS=Mus musculus OX=10090 GN=Pbld1 PE=1 SV=2 | 32 | 2 | 2 | 1 |  | |  | 355592 |
| O55111 | Desmoglein-2 OS=Mus musculus OX=10090 GN=Dsg2 PE=1 SV=3 | 122.3 | 1 | 1 | 1 | 83192 | | 79228 | 278956 |
| Q9D0W5 | Peptidyl-prolyl cis-trans isomerase-like 1 OS=Mus musculus OX=10090 GN=Ppil1 PE=1 SV=1 | 18.2 | 10 | 1 | 1 |  | |  |  |
| Q8CIE0 | Serpin A11 OS=Mus musculus OX=10090 GN=Serpina11 PE=2 SV=1 | 47.2 | 3 | 1 | 1 |  | |  | 489862 |
| Q640N1 | Adipocyte enhancer-binding protein 1 OS=Mus musculus OX=10090 GN=Aebp1 PE=1 SV=1 | 128.3 | 1 | 1 | 1 |  | |  | 182041 |
| P01592 | Immunoglobulin J chain OS=Mus musculus OX=10090 GN=Jchain PE=1 SV=4 | 18 | 5 | 5 | 1 | 4086682 | | 2407511 | 6620906 |
| Q91W43 | Glycine dehydrogenase (decarboxylating). mitochondrial OS=Mus musculus OX=10090 GN=Gldc PE=1 SV=1 | 113.2 | 4 | 1 | 1 |  | |  |  |
| Q99JY9 | Actin-related protein 3 OS=Mus musculus OX=10090 GN=Actr3 PE=1 SV=3 | 47.3 | 2 | 1 | 1 | 163374 | |  | 227038 |
| A3KMP2 | Tetratricopeptide repeat protein 38 OS=Mus musculus OX=10090 GN=Ttc38 PE=1 SV=2 | 52.2 | 2 | 1 | 1 |  | |  | 716408 |
| D3YYU8 | Obscurin-like protein 1 OS=Mus musculus OX=10090 GN=Obsl1 PE=1 SV=1 | 197.8 | 1 | 2 | 1 | 83668 | | 189178 | 483561 |
| O35286 | Pre-mRNA-splicing factor ATP-dependent RNA helicase DHX15 OS=Mus musculus OX=10090 GN=Dhx15 PE=1 SV=2 | 90.9 | 2 | 1 | 1 |  | |  | 10416 |
| O70468 | Myosin-binding protein C. cardiac-type OS=Mus musculus OX=10090 GN=Mybpc3 PE=1 SV=1 | 140.5 | 1 | 4 | 1 | 12286466 | | 1290056 | 7286634 |
| Q99P69 | Kinetochore protein Nuf2 OS=Mus musculus OX=10090 GN=Nuf2 PE=1 SV=2 | 54.6 | 4 | 3 | 1 | 5171029 | | 4221761 | 8339384 |
| P29268 | CCN family member 2 OS=Mus musculus OX=10090 GN=Ccn2 PE=2 SV=3 | 37.8 | 5 | 1 | 1 | 54355 | |  | 18802 |
| Q8BGT1 | Leucine-rich repeat transmembrane protein FLRT3 OS=Mus musculus OX=10090 GN=Flrt3 PE=1 SV=1 | 72.8 | 1 | 1 | 1 |  | |  | 182583 |
| P97364 | Selenide. water dikinase 2 OS=Mus musculus OX=10090 GN=Sephs2 PE=1 SV=3 | 47.8 | 4 | 1 | 1 |  | |  | 2216857 |
| O55128 | Histone deacetylase complex subunit SAP18 OS=Mus musculus OX=10090 GN=Sap18 PE=1 SV=1 | 17.6 | 5 | 3 | 1 | 147683814 | | 178508094 | 4719085 |
| Q45VK7 | Cytoplasmic dynein 2 heavy chain 1 OS=Mus musculus OX=10090 GN=Dync2h1 PE=1 SV=1 | 492 | 0 | 2 | 1 |  | | 1178523 | 15805282 |
| P48760 | Folylpolyglutamate synthase. mitochondrial OS=Mus musculus OX=10090 GN=Fpgs PE=1 SV=3 | 64.9 | 2 | 1 | 1 | 81728 | | 116395 | 160339 |
| Q00724 | Retinol-binding protein 4 OS=Mus musculus OX=10090 GN=Rbp4 PE=1 SV=2 | 23.2 | 4 | 1 | 1 |  | |  |  |

**Supplementary appendix 3.**

**Supplementary appendix 3. Complementary methods.**

**Size exclusion high performance liquid chromatography**

*Analytical size exclusion:* 20 μl of the individual eluates were diluted in 25 μl of running buffer (0.1 M Na_2_SO_4_. 0.05 M Na_2_HPO_4_•2H_2_O. 0.05 M Na_2_H_2_PO_4_•2H_2_O) and 1 μl pooled plasma was diluted in 99 μl running buffer; 20 μl was injected in the HPLC system using a Gilson 234 autoinjector (Gilson Inc.. Middleton. WI)). The proteins were separated on a 4.6 mm ID × 30.0 cm TSKgel Super SW 2000 and a 4.6 mm ID × 30.0 cm TSKgel Super SW 3000 column coupled in series (Tosoh Biosciences. Stuttgart. Germany) and detected at UV 220 nm (UVD170. Dionex) with an eluent flow rate of 0.35 ml/min (Ultimate 3000 RS pump. Dionex. Sunnyvale. CA. USA). A pool of 1 mg protein was generated for each eluate treatment (HS. HO. NS) with equal contribution of >50 kDa proteins from the replicates based on the area under the UV220-curve. The large volumes (about 7ml) were concentrated using a 3 KDa Amicon filter (Millipore. Billerica. MA) and buffer exchanged to Ringers solution to a final volume of about 125 μl.

*Preparative size exclusion:* The pooled samples (60 μl of 125) were fractionated using the same HPLC system as described above with a 7.8 mm ID × 30.0 cm TSKgel G2000SWXL column and a 7.8 mm ID × 30.0 cm TSKgel G3000SWXL column coupled in series. A flow rate of 0.5 ml/min was applied and 1 ml fractions collected using a fraction collector (Gilson).

**Sample preparation of SEC fractions for proteomics and mass spectrometry**

The six preparative SEC HPLC fractions from the pooled samples were exchanged into 100 mM ammonium bicarbonate and concentrated to a final volume of 25 μl using Amicon 50KDa cut-off filters (Millipore). The samples were then denatured by addition of 25 μl trifluoro-ethanol (Sigma-Aldrich T-8132) and 2.5 μl dithiothreitol (Sigma-Aldrich D-5545) at 90°C for 45 min. reduced and alkylated with iodoacetamide. then digested at 37°C overnight by trypsin (Sigma-Aldrich T6763. protease-to-protein ratio of 1:35) in accordance with the protocol provided by Agilent Technologies. Santa Clara. CA. USA (http://www.chem.agilent.com. part number USHUPO3). The tryptic peptide samples were desalted by C-18 spin columns (Pepclean C-18. 89870. 158 Pierce. Rockford. IL) . evaporated in a sped-vac concentrator (Eppendorf) and reconstituted in 100 μl 0.1 % trifluoracetic acid (TFA) prior to MS analysis.

**Mass spectrometry analysis**

The 21 trypsinized samples (7 SEC fractions from NS. HS and HO pools) were analyzed at the Proteomics Unit at University of Bergen (PROBE) using an Orbitrap Velos Pro mass spectrometer (Thermo Scientific). The sample (0.5 µg of peptides) was loaded onto a pre-column (Dionex. Acclaim PepMap Nano Trap column. C18. 75 µm i.d. x 2 cm. 3 µm) followed by separation on the analytical column (Dionex. Acclaim PepMap100 RSLCnano column. 75 µm x 15 cm. C18. 2 µm) using a Dionex Ultimate NCS-3500RS LC system (Sunnyvale. CA) coupled online to an LTQ Orbitrap Velos Pro mass spectrometer (Thermo Scientific. Waltham. MA). A 90 min LC-method using a gradient composition of mobile phase A (0.1% FA / 2% ACN) and mobile phase B (0.1% FA / 90% ACN) was applied. After 8.5 min with sample loading at 2% B. the gradient ramped from 8 to 38% B over 61.5 min. then from 38 to 90% B over 3 min. and stayed at 90% B for 5 min. The column was finally conditioned with 12 min of 5 % B. The peptides were continuously eluted using a nanopump flow rate of 280 nL/min and ionized in the electrospray (1800 V. 260 degrees C) as they were sprayed into the Orbitrap instrument. The Orbitrap was operated in the DDA-mode (data-dependent-acquisition) to automatically switch between full scan MS and MS/MS acquisition. Instrument control was through Tune 2.6.0 and Xcalibur 2.1. Survey full scan MS spectra (from 300-2.000 m/z) were acquired with resolution. R = 60.000 at 400 m/z (after accumulation to a target value of 1e6 in the linear ion trap with maximum allowed ion accumulation time of 500 ms). The 20 most intense eluting peptides above an ion threshold of 1000 counts. and charge states of +2 or higher. were sequentially isolated to a target value of 1e4 and fragmented in the high-pressure linear ion trap by low-energy CID (collision-induced-dissociation) with normalized collision energy of 35% and wideband-activation enabled. The maximum allowed accumulation time for CID was 200ms. the isolation width maintained at 2Da. activation q = 0.25. and the activation time was 10ms. The resulting fragment ions were scanned out in the ion trap at normal scan rate. and recorded with the secondary electron multipliers. One MS/MS spectrum of a precursor mass was allowed before dynamic exclusion for 30s. Lock-mass internal calibration was not enabled.

**Protein identification and label-free quantitative proteomics**

The Orbitrap data files were analyzed using the label-free MS1 feature based quantitative proteomics software Progenesis LC-MS (Nonlinear Dynamics Ltd. Newcastle. UK) as ‘fractionated samples’ as recommended by the provider. The seven fractions were analyzed as separate experiments (fraction wise) before the experiments were combined in the end of the workflow. For the respective experiment. the LC-MS runs (raw files) for the three conditions (NS. HS. HO) were imported and aligned based on the m/z and retention time. The MS1 peaks with charges +2 to +7 were picked. Progenesis uses the volume of these peaks for quantification. The MS1 peaks with corresponding MSMS spectra were exported as mgf files and searched to identify peptides and proteins using proteome discoverer with Mascot (Thermo Scientific) matching experimental data against the. Rattus norwegicus and Mus musculus Swiss-Prot protein databases. The relative protein abundances reported are based on the sum of normalized abundances of unique peptides corrected for variance in the global intensities of all features.
